# Impact of Procedures and Human-Animal Interactions during Transport and Slaughter on Animal Welfare of Pigs: A Systematic Literature Review

**DOI:** 10.3390/ani12233391

**Published:** 2022-12-02

**Authors:** Rudi Isbrandt, Mechthild Wiegard, Diana Meemken, Nina Langkabel

**Affiliations:** 1 Institute of Food Safety and Food Hygiene, Working Group Meat Hygiene, Department of Veterinary Medicine, Freie Universität Berlin, 14163 Berlin, Germany; 2 Institute of Animal Welfare, Animal Behavior and Laboratory Animal Science, Department of Veterinary Medicine, Freie Universität Berlin, 14163 Berlin, Germany

**Keywords:** fattening pig, handling, indicator, management

## Abstract

**Simple Summary:**

Animal welfare during the transport and slaughter of pigs is in the interests of consumers and producers. All procedures and human-animal interactions on the day of slaughter are potentially stressful for the pigs. Good handling practices—calm handling and a handling set to the individual needs of the pigs—and management of the animals provide the maximum animal welfare at slaughter, but only by avoiding or minimizing any further external stressors in addition to the transport and the slaughter itself. By conducting a systematic literature review, we wanted to find out which procedures and human-animal interactions were found by researchers to have an impact on welfare during pig transport and slaughter. Direct human-animal contact and the management of transport and slaughter procedures were identified as major influencing factors. As the animal welfare-sensitive areas of stunning, control of stunning, and bleeding are highly important, personnel should be trained regularly to ensure good practices and animal welfare. Deficient handling and procedures in the transport/slaughter processes can be critically scrutinized and corrected only when personnel are sufficiently educated. Knowledge of best handling practices is necessary to reflect on our own and other personnel’s behaviour and to maintain awareness of animal welfare. Animal suffering caused by humans is not acceptable.

**Abstract:**

Animal welfare is a high social and political priority and is enshrined in European and national legislation. This systematic literature review (funded by the German Ministry of Food and Agriculture; grant no. 2817806A18) was conducted to find animal and management-based indicators that could be influenced by changes in pig handling and management procedures on the day of slaughter and, therefore, will directly result in improved animal welfare. For this systematic literature review, following the PRISMA guidelines, we conducted a structured literature search using the databases PubMed^®^, Web of Science^TM^, and LIVIVO with set search terms and their combinations in German and English. Only peer-reviewed original articles from European countries from 2009 until 2022 that dealt with welfare during transport and/or slaughter of fattening pigs and offered potential measurable indicators on the basis of the animal or management, and either directly or indirectly recommended handling practices were included. We used the literature management system EndNote^TM^, and after duplicate removal, a total of 1099 records were found for further analysis. After analysis and discussion in the review team, which consisted of three experts in total, 105 articles underwent full-text screening. Finally, 39 articles were included in the results for this systematic literature review. According to this systematic literature review, the following procedures have a positive impact on the animal welfare of pigs on the day of slaughter. Calm—rather than rough—handling of pigs was the most influential factor. This can be achieved by using animal-friendly driving aids. Loud noise should be avoided in general or reduced as far as possible. Transport time is not always influenceable, but shorter transport duration with stocking density as stated by the European regulations as a maximum will reduce stress in pigs. Our review showed that there are differing scientific results about lairage duration, although this should be set in relation to prior stress and transport time. Knowledge of good stunning parameters, such as gas concentration, exposure time to the gas, recommended amperage, and current flow duration is essential. If electrical stunning is performed, the correct attachment of electrodes is crucial. The control of stunning effectiveness is extremely important to avoid suffering and pain, with the proviso that it is assessed on the basis of multiple parameters. The corneal reflex, regular gasping, and the up-righting reflex could be assessed together. Certificates of competence and continuing educational programmes for the personnel form the basis of animal-friendly handling and a stressless slaughter routine. It can be assumed that the results of this systematic literature review reflect the state of current research of pig welfare during transport and slaughter in the European Union, even though it must be assumed that not all relevant points were covered by the review, which can be seen as a limitation at the same time because important non-European or older publications could not be included.

## 1. Introduction

Farmed animals are slaughtered for food production. The fact that animals are allowed to be slaughtered and under which conditions is already regulated in the German Animal Welfare Act §4a [1]. The day of slaughter, including transport and procedures at the abattoir, is stressful for pigs [2]. As a consequence, it is imperative that animal welfare-focused treatment continues during the whole life of farmed animals [3]. Deficiencies in the day-to-day practice during animal transport and slaughter are known [3]. The aim of animal welfare-friendly slaughter is to reduce the pain, stress, and suffering of the animals during the slaughter procedure [4]. Therefore, the personnel have to be well-educated, well-paid, and work under appropriate conditions [3]. A survey in Germany showed that for 13.1% of the consumers questioned the issue of animal welfare was meaningless, a quarter were indifferent to it, and in addition 15.2% believed that there is nothing wrong with the way in which farmed animals are kept today [5]. This shows that a large part of the population accepts the practices of livestock farming and also the fact of slaughtering an animal intended for human consumption.

The Treaty of Lisbon recognized animals as sentient beings, and the European member states agreed to protect them from pain and suffering [6]. When dealing with the slaughter of farm animals, European legal principles form the basis for animal welfare and the interaction of people with the animals, e.g., producers on the farm, transporters and personnel at the abattoir. To achieve this, European regulations regarding animal welfare during transport and slaughter are set, laying down specific requirements for all animal species. The Council Regulation (EC) No 1/2005 on the protection of animals during transport and related operations and its amending directives, lay down general conditions for the transport of animals, transport planning obligations, transporters, training courses, and the certificate of competence [7]. In the annex, definitions of fitness for transport and transport practices, regulations for watering, feeding, journey time, resting periods, and space allowance for the different species are described [7]. The Council Regulation (EC) No 1099/2009 on the protection of animals at the time of killing sets out the general requirements for killing and related operations, stunning methods and control of stunning, and handling and restraining operations at abattoirs, explains the concepts and tasks of the animal welfare officer and regulates the certificate of competence for stunning and killing animals [8]. In Germany, there are additional animal welfare-related regulations in force specifying the European requirements set in Regg. (EC) No 1/2005 and No 1099/2009 and defining possible punishments after misconduct, such as verbal warnings, monetary fines, withdrawal of certificates, and even imprisonment [9,10].

To uncover and mitigate deficits in animal welfare, researchers are increasingly focused on indicators to identify steps for possible improvements along the whole production chain. Measurable indicators regarding animal welfare can be resource-based (e.g., number of drinkers, size of laying surface, ventilation system), animal-based (e.g., body condition, lesions on skin, lameness, blood parameters), and management-based (e.g., stocking density, ventilation and hygiene management, competence of certificate) [11]. 

We conducted a systematic literature review (SLR) on pig-welfare-related issues and indicators. The first aim was to identify management-based indicators and to determine how handling practices could be changed to improve animal welfare. Secondly, we investigated how animal-based indicators could be used to measure and improve animal welfare.

## 2. Materials and Methods

Out of the joint research project “eSchulTS^2^” (development of target group-specific e-learning modules to improve animal welfare during the transport and slaughter of cattle and pigs) we conducted a SLR following the PRISMA (Preferred Reporting Items for Systematic Reviews and Meta-Analyses) guidelines [12] Supplementary Material (S1). The following search terms and combinations were set in German and English, covering the processes of pig transport and pig slaughter:

(pig OR swine) AND (animal welfare OR welfare) AND (transport)

(Schwein OR Mastschwein) AND (Tierwohl OR Tierschutz) AND (Lebendtiertransport OR Tiertransport OR Viehtransport OR Transport)

(pig OR swine) AND (animal welfare OR welfare) AND (slaughter OR slaughterhouse OR abattoir OR lairage OR bleeding OR stunning)

(Schwein OR Mastschwein) AND (Tierwohl OR Tierschutz) AND (Schlachtung OR Schlachthaus OR Schlachtbetrieb OR schlachten OR Schlachthof OR Tötung OR Betäubung OR Entblutung OR Wartestall).

The inclusion and exclusion criteria are listed in Table 1. The authors have set the search period starting in 2009, as the relevant Council Regulation (EC) No 1099/2009 [8] was adopted in that year. Consequently, the place of publication was also narrowed down to countries that apply European law.

**Figure 1:**
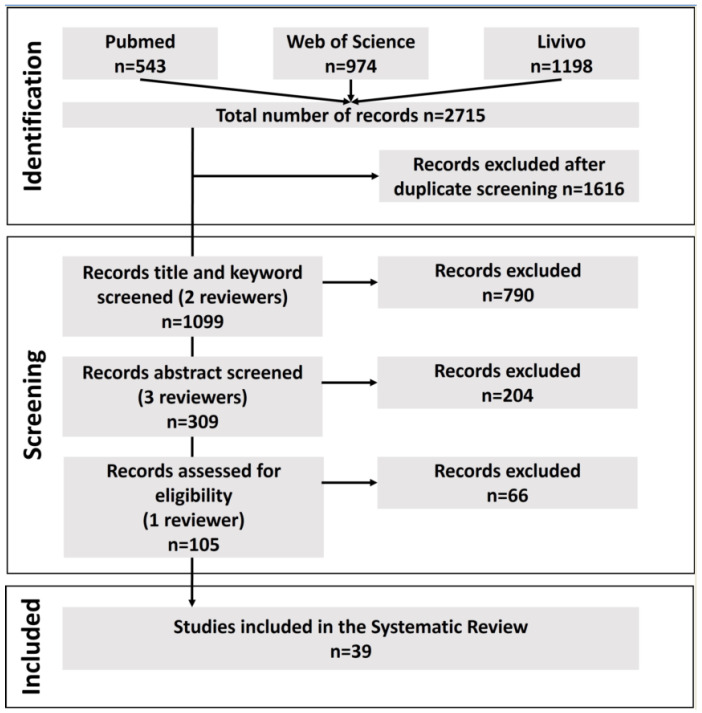
Record extraction and review process for the literature search during the systematic literature review to find impacts of procedures and human-animal interactions during transport and slaughter on animal welfare of pigs in accordance with the PRISMA (Preferred Reporting Items for Systematic Reviews and Meta-Analyses) guidelines [12].

The databases PubMed^®^, Web of Science^TM^ and LIVIVO were used and 2715 literature records on animal welfare during the transport and slaughter of pigs were found. The last date of searches in all databases was 28 January, 2022. More information on the search and filters used for the databases can be found in the Supplementary Material (S2). The literature management system Endnote^®^ was used to collect all records found in all databases and to eliminate duplicates. In a second step, remaining duplicates were removed by hand, resulting in 1099 records, and the hitlist was exported as an Excel^®^ file. After this, the review team started with the review process. The team consisted of three reviewers in total. Two researchers from the Institute of Food Safety and Food Hygiene, Working Group Meat Hygiene—one meat hygiene professor and one pre-doctoral veterinarian—and one researcher from the Institute of Animal Welfare, Animal Behaviour and Laboratory Animal Science—a post-doctoral veterinarian—to ensure overlapping fields of expertise and appropriate review results. Firstly, two reviewers screened the titles and keywords for relevance (see Figure 1). Altogether, 790 publications did not fit in the inclusion criteria for title or keywords. Next, 309 publications underwent abstract screening by all three reviewers for relevance and the inclusion and exclusion criteria. If just two out of three reviewers judged a publication suitable with regard to the inclusion and exclusion criteria, all three reviewers discussed whether to include the publication or not, in order to find an overall agreement. If only one reviewer considered a publication as suitable, it was excluded from full-text reading. After assessment and discussions, 105 articles were included in the full-text screen by one of the reviewers in close consultation with the others. Finally, 39 records were included in the SLR. A list of all records and a list of the included publications is available as Supplementary Materials (S3 and S4). The process of screening and reviewing the records found is shown in Figure 1. 

## 3. Results

The search resulted in 1099 unique publications in all three databases. After reviewing and full-text screening 105 studies, a total of 39 publications were included in the results of this SLR (Figure 1).

### 3.1. General Aspects of Human-Animal Interaction and Management Factors

Some generally applicable statements for handling procedures of pigs during transport and slaughter were found in the publications. Vitali et al. [13] concluded that pre-slaughter conditions and pig handling can result in negative social pig behaviour. Some authors made statements about good or gentle/calm handling contrary to bad or rough handling, e.g., when using an electric prod [14,15,16]. Cortisol was measured as a stress marker and its levels increased in pig saliva and blood on the day of slaughter, which can be associated with handling procedures on that day [14,15]. Contrary, Dokmanovic et al. [16] determined that blood cortisol did not differ between pigs exposed to gentle or rough handling. However, they also stated that blood lactate was significantly higher after rough handling.

Vermeulen et al. [17] showed that stress on the day of slaughter can influence the pH 30 min post-mortem (pH_30min_), and that low pH_30min_ indicated a risk of PSE development, especially when handling procedures were categorised as deficient and critical handling occurred close to the timepoint of stunning. Moreover, using the pH_30min_ or pH_60min_ ratio as an indicator, significantly lower values were seen after the use of electric prods or rough handling [16,18], and likewise, significantly higher values of pH_30min_ were measured after gentle handling [16]. Brandt et al. [19] showed that stress on the day of slaughter, especially events in the race—the way directly to the stunner—can influence the pH_22h_ significantly and, therefore, could be a good welfare indicator. In contrast, Dokmanovic et al. [16] did not find significant differences in the pH_24h_ with respect to gentle or rough handling. It was also shown that abrupt changes of stressful and stressless procedures on the day of slaughter can lead to a higher incidence of PSE meat [17].

Some authors showed that the loading and handling events, such as driving to move by personnel had a significant effect on increasing the heart rate [2,20].

Dalmau et al. [21] identified lower percentages of pigs vocalising if automatic gates were used compared to electric prods. Another study showed that after the use of electric prods, around one fifth of the pigs showed behaviours such as slipping, falling, high-pitched vocalisation, or jumping on the pigs in front [16]. Dalmau et al. [21] reported that the personnel who handle the animals can be a main risk factor for the pigs’ general fear, expressed as reluctance to move. When handling pigs, according to von Wenzlawowicz et al. [22], behavioural principles should always be taken into account and implemented in day-to-day work. Consequently, animals should not be driven actively, but more attracted and, therefore, encouraged to walk [22]. 

No significant difference in the meat colour after gentle or rough handling was seen, whereas significantly higher meat temperatures were found after rough handling [16].

To improve the process steps prior to slaughter, handling should be adapted to daily conditions and not follow a rigid scheme because this would not ensure the improvement of animal welfare [23]. The adjustment of the slaughter line speed and number of abattoir personnel are important factors [24]. Moreover, results of inspections, e.g., animal welfare protocols, carried out by official veterinarians or by the food business operator at the abattoir, should be taken into consideration [21]. Dalmau et al. [21] showed that deficiencies in slaughter line speed or slaughter capacity could be identified after such audits, and as a result, positive investments for animal welfare, such as automated doors, were made. Addressing these points and adjusting them, if necessary, could result in minimised use of electric prods. Valkova et al. [25] recorded injuries of animals during the slaughter process and they stated that a low frequency of traumatic injuries, with 0.003% for finishing pigs, indicates good conditions on farms and during the day of slaughter.

### 3.2. Loading and Transport

Regarding pig transport, there were different potential factors and procedures influencing animal welfare [2] and, indirectly, the meat quality [26,27].

#### 3.2.1. Handling, Management and Factors Associated with the Truck Driver

Brandt et al. [28] showed that the driving of pigs during loading occurred in nearly 50% of handling interventions.

Weather is a factor that cannot be influenced by the personnel directly, but among other factors, appropriate ventilation or cooling devices should be functional in extremely high outdoor temperatures and need to be checked by the drivers regularly [29]. In general, Brandt et al. [28] found a significant negative correlation between blood plasma glucose concentration after assessing an animal welfare index based on the Welfare Quality^®^ protocol determined during transportation. 

Drivers can induce stress in pigs during different transport steps, because they are also present during loading and unloading [27]. The evaluation of truck drivers and their behaviour by using meat quality parameters is challenging as these parameters were influenced by many factors [27]. It was shown that low average vehicle speed resulted in higher blood cortisol and creatine kinase (CK) levels, showing that pigs were more stressed, even after relatively short transport durations of up to 90 min [30]. One reason for this could be using highways instead of rough roads, which are travelled at higher speeds, creating less stress for the pigs [30]. Rough driving can be responsible for higher skin lesion rates and damages [31]. Gerritzen et al. [20] reported that during transportation the heart rate of pigs increased at the end of a break, and more frequent and longer fights between pigs occurred after breaks. Driessen et al. [31] concluded that among other factors, the driving style can be improved by education in training programs.

Garcia-Diez and Coelho [32] noticed a significant reduction in death during pig transportation and rejection due to ante-mortem inspection when drivers and transporters had obtained compulsory certification. Negligent driving was noticed by Nannoni et al. [33] as the reason for the death of 32 pigs during one transportation. 

#### 3.2.2. Stocking Density during Transport

Gerritzen et al. [20] investigated the influence of stocking density during transport on the animal welfare of pigs, measuring different blood parameters and the heart rate. They could not show significant correlations and concluded that the absence of significant connection of heart rate and blood parameters with stocking density indicates that pigs potentially can adapt to the journey conditions when they are transported with more space of 179 kg/m^2^ [20]. Gerritzen et al. [20] noticed a slight boost of heart rate in pigs transported under low stocking density conditions immediately before and after departure and the lowest heart rates during the main part of the journey and the break. After the break, the heart rate again increased [20]. 

In addition, it was found that the body temperature in pigs transported with a normal stocking density of 235 kg/m^2^ was higher than that of pigs transported in low stocking density conditions [20]. The measured body temperature tended to increase during the break and after arrival [20].

Lower stocking density during transport was related to higher meat pH_48h_ [27]. Moreover, higher pH_30min_ values could result if the stocking density ranged from 196 to 256 kg/m^2^ [17,18]. Another study determined that stocking densities of 333 kg/m^2^ or higher resulted in significantly lower pH_45min_ and a significantly higher incidence of PSE meat, whereas densities of 200 kg/m^2^ or lower resulted in significantly higher pH_45min_ and a higher incidence of dark, firm, and dry (DFD) meat [34]. Vermeulen et al. [17] concluded that a non-optimal stocking density can result in welfare issues and, accordingly, increases the risk of PSE meat development.

Taking the carcass temperature into account, a significant interaction with stocking density was described and higher stocking density led to higher carcass temperature measured 45 min post-slaughter [34]. In agreement with this, Driessen et al. [27] observed that lower stocking density was significantly correlated with a lower carcass temperature. 

Measuring carcass skin lesions produced contradictory results: low stocking densities with 200 kg life weight on at least 1 m^2^ were significantly correlated with higher skin lesion scores [34], whereas others did not find any influence of stocking density on the amount or intensity of skin damages [31]. Comparing moderate, i.e., a common stocking density of 235 kg/m^2^ and low stocking density of 179 kg/m^2^, significantly more fighting was observed during journeys with low stocking density [20]. Pigs that were transported in common stocking density up to a 2 h duration showed less activity [20]. More animals were sitting at the beginning of transportation, whereas with enough space and the lower stocking density, pigs laid down, which indicates a benefit in pig welfare during transport [20]. After a pause for the driver, more frequent and longer fights were observed in pigs transported with normal stocking density [20].

Taking pre-slaughter losses—numbers of dead pigs before slaughter—in a retrospective study into account, Nannoni et al. [33] could show significantly higher losses when pigs were transported with lower stocking density.

#### 3.2.3. Transport Duration

Regarding the impact of transport duration on the development of PSE meat, transports of under 3 h—compared with up to 7 h [23]—resulted in less acceptable meat conditions, such as low pH, lower colour index, and higher drip loss [23,27].

Body injuries in pigs can originate on the farm, during transport or at slaughter [25], and findings show that there is space for the improvement of the treatment of pigs during transport [35]. Driessen et al. [31] could not find any relationship between skin lesions and the transport duration. 

Nannoni et al. [33] could show that after transport durations of 90 min or more pre-slaughter losses were significantly higher, which is in accordance with Vitali et al. [29] who concluded that for heavy slaughter pigs the risk of dying increases significantly for transport durations over 2 h.

Sardi et al. [30] showed that pigs that were transported a slightly longer distance had lower blood levels of cortisol and CK.

### 3.3. Unloading

An expert panel determined that the unloading process was highly relevant regarding the animal welfare of pigs [2], and by measuring the saliva cortisol levels, the time of arrival at the abattoir was classified as a medium stressor for pigs [14,36]. Additionally, the CK concentration in the sticking blood plasma was correlated to the handling and behaviour of pigs during the unloading process [19]. Driessen et al. [27] measured unloading durations of pigs at abattoirs, which included large deviations but showed no effect on the observed meat quality—pH_45min_, carcass temperature, pH_48h_, meat colour—parameters. Nonetheless, the heart rate increased while pigs arrived, were unloaded, and went into lairage [20], with a significantly higher heart rate measured during the unloading process [2].

Differences existed between the dead-on-arrival rate—death during transportation—and the dead-in-pen rate—death during lairage [29,32]. Losses before animals were slaughtered often occurred during transport [33], which resulted in a higher dead-on-arrival rate [29,32]. The dead-on-arrival rate can be measured during unloading. One of many influencing factors for differences in the number of dead pigs could be inadequate ventilation during transport due to the truck’s lack of movement or the installed fans being insufficient [29]. Therefore, a transport should be classified “ok” if no pigs arrived dead at the abattoir, which is associated with lower risks of developing PSE meat [17], and there is a loading density between 190–244 kg/m^2^ [17].

The use of electric prods and forcing pigs to move during the unloading procedure were found to be the main human-animal interventions at this step [24,28]. In the study of Sardi et al. [30], the group of higher stressed pigs was connected with more handling, which in itself tended to provoke more physical reactions, such as falling or slipping during unloading. Dalmau et al. [37] observed that slipping and falling occurred to nearly 66% of pigs. The authors identified management problems as a reason for the pigs’ fear, expressed by turning back and reluctance to move, in European pig abattoirs [38]. 

### 3.4. Lairage and Duration of Lairage

The aim of lairage is to give the animals the chance to recover from potential previous stressors [39,40] and was scored as very important; therefore, lairage parameters, e.g., skin damage and duration of lairage, should be established in animal welfare measurements [2]. Additionally, lairage conditions can have a big impact on meat quality [27], for this reason, lairage is implemented in abattoirs [39]. 

Brandt et al. [19] measured plasma CK concentrations, but did not find an obvious connection with the way in which pigs were handled during lairage. A higher temperature of the showering water, which can be controlled by the personnel, significantly influenced the pH_30min_ of muscle in a positive way [18]. Vermeulen et al. [17] demonstrated that there is the potential for pigs to recover during lairage, as pH_30min_ was higher if all steps except transport were assessed as being adequate. Pigs can appear relaxed if they are lying down, although this can also be an indicator of exhaustion, and it was not possible for the authors themselves to differentiate between these two causes by observation alone [19]. Lairage was the step with the second highest death rate, after death during transportation, with animal losses in lairage of around 39% [33].

If the lairage pen is overcrowded, pigs can show mounting behaviour, such as jumping with forelimbs or the sternum on the back of the another pig in front, the results of which can be seen after slaughter as lesion-like scratch marks on the caudal carcass [31]. Vitali et al. [29] concluded that good ventilation during lairage and more space for the pigs allow good air circulation and can support the animals´ regulation of body temperature, minimising deaths in the lairage pens, especially in the summer.

#### 3.4.1. Blood and Saliva Parameters

Lairage duration with respect to pigs’ blood parameters has been widely discussed and researched. Several studies investigated the effect of different stress parameters measured in blood—such as CK, blood lactate, haptoglobin, C-reactive protein, ratio of neutrophiles:lymphocytes—or in saliva—such as cortisol, salivary alpha-amylase, total esterase activity, butyrylcholinesterase, lactate dehydrogenase, and oxytocin [14,15,16,36,39,40]. Contradictory results were observed for the different time durations in lairage that were compared in these studies, but, nonetheless, an increase in blood parameters indicating stress was most frequently observed when lairage time was longer. In saliva, the measured cortisol level was lower with shorter lairage durations of up to 2 h and increased when the lairage duration was lengthened [14]. Rey-Salgueiro et al. [14] concluded that this indicates high stress in pigs during longer lairage periods, even though it is assumed that longer lairage should help the pigs to recover. A doubling of both haptoglobin and C-reactive protein in the sticking blood after 3 h of lairage was seen, compared to 12 h of lairage, and could be used as a parameter to indicate early stress in pigs [40]. 

#### 3.4.2. Meat Quality Parameters 

Whereas some studies did not find a difference in meat quality parameters, such as pH, drip loss, and cooking loss of pigs after short or long lairage [15,16,18], and similar incidences of PSE meat after short and long lairage [15], other studies found a higher risk of lower pH_45min_ and resulting PSE meat after lairage of up to 2.7 h [16,34]. Contrarily, Driessen et al. [27] observed better meat quality in their study in which lairage duration ranged from 2 min up to 165 min after longer lairage. In contrast Garcia-Celdran et al. [40] found a significantly higher pH_45min_ after 3 h lairage—compared with 12 h lairage—and another study found a significant increase in pH_45min_ after lairage for more than 17 h [34]. 

Vermeulen et al. [17] reported that a closer time span between pre-slaughter handling and the stunning procedure could be a critical point for a higher incidence of PSE meat. The same was seen for higher rates of juiciness, tenderness, palatability, drip loss, and shear value when pigs were slaughtered immediately or lairage was less than 2 h [15,16,23]. Rey-Salgueiro et al. [14] and Mantis et al. [23] came to the conclusion that 2–4 h lairage time can be appropriate if managing the pigs does not produce stress. As found in other studies, longer lairage of 14–22 h resulted in better meat quality [16,39], and overnight lairage could also be applied, as it was observed, to result in the pigs being more quiet during stunning and showing fewer muscle spasms and ruptured capillaries as a result [41].

Investigating the incidence of PSE meat regarding the sex of the pigs, no differences were seen, as after short lairage there was a low risk of PSE meat developing from both female and male pigs [39].

#### 3.4.3. Skin Lesions

Skin lesions or injuries can be used as animal welfare indicators in pigs. Panella-Riera et al. [42] did not find an interaction between lesions on skin and lairage times. However, after lairage times of above 14 h, other authors observed significantly higher skin lesion scores or more blemishes [16,34,39]. Long lairage led to higher numbers of lesions on the middle of the carcass and the ham [31], and it was observed that overnight lairage was the reason for significantly more scratches on the front [41] or lesions at the tail of pigs [13]. 

### 3.5. Race to the Stunner

On their way to the stunning point, pigs can show turning back behaviour, which can be the result of constructional deficiencies in the abattoir, but also personnel-behavioural deficiencies while handling the animals [21] can be a cause. One reason for many animals being moved with electric prods, with the result of vocalising pigs, was bad management that led to long waiting times in the race and unsteady animal flow to the slaughter room [24]. 

Brandt et al. [19] concluded that lactate concentration in exsanguination blood plasma could be a good indicator for welfare-relevant events directly before stunning, because they found interactions to slipping, falling, and pigs being moved by gate in the race. 

The ambient-sound noise level when pigs are on their way to the stunner had a significant negative effect on meat pH_30min_ [18]. Vermeulen et al. [17] concluded that the last stages of handling, in the race to the stunner and, especially when electric prods were used, the ambient noise and a bad quality of stun influenced the pH_30min_ significantly and led to a higher incidence of PSE meat.

### 3.6. Ambient Noise

Different studies showed that the sound level on the day of slaughter at different processing steps should not exceed 85 dB [17,43], and, therefore, a recommended maximum threshold of 85 dB for all pre-slaughter steps was given by Vermeulen et al. [43]. 

Moreover, higher noise level correlated with higher heart rates and a lower pH_30min_ and, therefore, higher incidence of PSE meat [43]. The influence of noise on meat pH was also mentioned by other authors [17,18]. In addition, noise changes had an impact and significantly correlated with the incidence of PSE meat [26]. 

The background noise level could also be an influencing factor for significantly increased saliva cortisol, as hypothesised by Rey-Salgueiro et al. [14].

### 3.7. Mixing of Animals

Mixing is the process of regrouping pigs and it is performed during transport to have groups of pigs with a similar size and weight, and it is common practice to divide each truck into different compartments [27], but it can be seen as a critical control point [31]. 

Mixing could be one factor for more observed fighting and mounting behaviour [44], as well as higher stress parameters, such as cortisol levels [14,30,44]. In addition to that, no significant differences in average blood cortisol concentrations were found between different group compositions—mixed males, unmixed males, and males mixed with females—whereas in pigs that were involved in mounting behaviour cortisol levels were higher [44]. Additionally, mixing pigs on the farm—prior to transport—or in the lairage did not influence pH_45min_ or pH_48h_ significantly [45]. 

In general, if no mixing of pigs is performed fewer skin lesions may result [38], whereas higher skin lesion scores can be associated with mixing [46]. When pigs were not mixed while loading or in lairage, lower skin lesion scores were observed [45], whereas higher skin lesion scores occurred on the carcasses after mixing before transport [31,45], during loading, and in lairage [45]. Conversely, van Staaveren et al. [44] did not find a significant interaction between mixing and skin lesion scores for the different groups in the study. Nonetheless, mixing intact males can increase mounting behaviour, aggression, and fighting events significantly [20,44], and, consequently, more carcass lesions occurred on these mixed pigs. However, such damages are not always visible on the skin [44] and, therefore, cannot always be evaluated after scalding. On the other hand, the lesion scores for the ears were significantly higher for mixed male groups because they showed significantly more ear-, flank-, and tail-directed behaviours [44]. 

The quality of meat [27,45] can be improved if pigs are not mixed [45].

According to Driessen et al. [31], the undesirable management method of mixing pigs during the day of slaughter should be addressed during animal-welfare training programmes.

### 3.8. Stunning Procedure

As shown by van de Perre et al. [18], a positive effect on meat pH_30min_ was seen if the stunning procedure was effective, e.g., after usage of gas concentrations above 80%. This was in accordance with Vermeulen et al. [17], who found a significant influence of better stunning quality on both the pH_30min_—higher values when the stun quality is better—and lower risk of PSE meat development.

#### 3.8.1. Electrical Stunning

Stress-free handling of pigs before electrical stunning should be the aim, and the following data show that a positive effect occurs on the correct manual placement of the electrodes [22]. In order to perform a stunning procedure in accordance with animal welfare standards, the position of the electrical tongs is essential and could be an assessment criterion, together with the contact of the electrodes at the end of the tongs and the electrical parameters used [22,24]. Consequently, dirty electrodes must be cleaned by the personnel [47]. Wrong electrode placement can lead to pigs vocalising and seems to mainly cause a higher percentage of pigs with signs of consciousness [24]. As noticed in a study, no vocalisation during electrical stunning was observed if the tong position was correct [48]. Regardless of the method of electrical stunning, more pigs regained consciousness with wrong electrode positioning and/or electrical parameters [22].

After head-only electrical stunning of pigs, more deficient stunning operations were observed in comparison to other methods, such as simultaneous head and heart cycle or automatic electrical stunning [22]. However, when an additional heart cycle was applied, pigs showed fewer signs of regaining consciousness [48]. Von Wenzlawowicz et al. [22] observed effective stunning after amperage application from 8 to 18 s and after short stun-to-stick intervals with a maximum of 10 s [22]. Two studies showed that the duration of the application of the current flow had no effect on stunning efficiency [47,49], whereas after the application of higher amperage, the pigs remained unconscious for longer, and, therefore, stunning effectiveness was higher [49].

Comparing manual electrical stunning to automatic electrical stunning with three electrodes for simultaneous head and heart cycle, the latter stunning system was more effective and resulted in fewer stunning failures [22]. Van de Perre et al. [18] also have shown an influence on pH. Stunning with three electrodes resulted in significantly lower pH_30min_ than using two electrodes or performing manual electrical stunning [18].

In summary, a single parameter of a minimum amperage is not enough in the sense of improving animal welfare during stunning, and 1.3 A, prescribed by legislation, did not significantly influence the stunning effectiveness [47]. Therefore, the stunning parameters have to be clearly communicated in the operating manual and have to be set individually after tests in each abattoir to ensure the effective stunning of pigs [47]. The amperage must not be reduced to improve carcass quality because improper stunning can result [22].

#### 3.8.2. CO_2_-Stunning

Stunning with carbon dioxide (CO_2_-stunning) is seen as an effective stunning method for pigs [22]. Effective stunning has positive effects on meat pH_30min_, which was observed when concentrations of CO_2_ above 80% were used [18]. Problems in the implementation of CO_2_-stunning could be a result of incorrect settings, such as gas concentration under 85%, gas temperature, time of exposure to the gas concentration under 130 s [22,50], and inappropriate slaughter speed or loading density of the gondolas, which should not exceed the recommended loading density during transport by European legislation [22].

The fact that even after long exposure to a high CO_2_ concentration some pigs regained consciousness during bleeding highlights the fact that a good back-up stunning method is required [22].

### 3.9. Control of Stunning Effectiveness

It is extremely important to ensure pig welfare during slaughter by continuously checking the efficiency of stunning [50]. For the control of stunning efficiency, different parameters can be used, such as observations of regular gasping or kicking, opening or closing of the eyelids, and control of the corneal reflex [24,38,50]. However, the stunning method has to be taken into account, because—after electrical—movements of limbs can be observed [50]. The control of only one parameter—for example, the corneal reflex—was advised against [50]. Several studies showed that a positive corneal reflex was often associated with regular breathing, blinking, pupillary reflex, and nystagmus [24,38,48,50]. Other signs of consciousness, such as pain reaction on the nose after pinching the nasal septum, vocalisation, and attempts to upright were also observed in various percentages [24,38,48]. After electrical stunning, Nodari et al. [48] observed that 35% of conscious animals after stunning showed tongue movements. Therefore, they highlighted that this parameter could receive more attention for the assessment of consciousness [48]. Additionally, it should be noted that vocalisation is the last appearing sign when pigs regain consciousness [21]. Atkinson et al. [50] developed a protocol with different risk levels and signs of inadequate stunning. The observation time could be of importance because Stocchi et al. [24] observed the signs in the first two thirds of the bleeding process, but only in two pigs directly after incision. This was also taken into account in the criteria that were summarised by von Wenzlawowicz et al. [22] for effective electrical and CO_2_-stunning. The stunning effectiveness should be checked between stunning and sticking, and again 30–60 s after sticking [22]. It could be seen as misbehaviour if animals are not monitored for stunning effectiveness directly after stunning and also during bleeding [22].

### 3.10. Sticking and Bleeding

Instant sticking is a technique that can minimise the risk of pigs regaining consciousness after stunning [50]. Inefficient bleeding can be the result of inappropriately small sticking incisions or an insufficient blood flow [22], and poor sticking practice can result in pigs regaining consciousness [50]. After CO_2_-stunning, late or insufficient bleeding resulted if too few employees were on the line or if they had too little work experience [22]. Atkinson et al. [50] mentioned that the stun-to-stick interval was significantly prolonged if the number of animals in the CO_2_-stunner increased, and the authors concluded that this led to significantly increased incidences of corneal reflex, nystagmus, and rhythmic breathing, all indicators of regained consciousness [50]. Similarly, another study found that significantly fewer pigs remained unconscious when using a longer stun-to-stick interval [49]. Head-only electrical stunning was effective if the sticking incision was made within the following first 10 s [22]. Végh et al. [49] concluded that after head-only electrical stunning, sticking should take place within the first 32 s, because they did not find differences in the percentages of pigs that remained unconscious. After a bleeding time of 254 s (+/−32 s), all pigs had relaxed muscles and no breathing was observed [24].

## 4. Discussion

### 4.1. Handling of Pigs and Factors Associated with the Truck Driver

Animal welfare-friendly handling of animals intended for human consumption should be a supreme principle. Animal-friendly driving methods to force the animals to move should always be used first [8]. Pigs can also be handled with a board, driving flag [51,52,53] or a paddle [52,53]. Finally, they also can be moved by using hands, but it is important not to make any noise throughout the whole driving process [51]. As shown in one study, nearly a third of finishing pigs were handled roughly, which shows that there is vast room for improvement [16]. In the EU, it is forbidden to hit or kick pigs, to produce avoidable pain or suffering on sensitive body parts, to lift pigs by their head, ears and legs, and to use prods or pointed objects (Council Regulation (EC) No 1099/2009 Annex III) [8]. The use of electric prods is regulated in the Council Regulation (EC) No 1099/2009, which states that electric prods can be used only on adult pigs if they refuse to move even if they are physically able to and have enough space in the front so movement is possible. The electric stimulus must not take longer than one second and is only allowed to be applied on the hindlimbs. The use of the electric prod is a means of last choice [8]. The German regulation on the protection of animals during slaughter [9] specifies that in pig abattoirs electric prods can be used for animals aged four months and older but only if animals will not walk along a single file chute shortly before stunning or directly before fixation for stunning [9]. The specification of age, therefore, makes the electric prod potentially usable for standard finishing pigs, with the special restriction of the location within the abattoir. Time pressure can be a negative factor that induces bad handling and the use of unsuitable driving objects, which should be removed [54]. The best method to move pigs forward is to drive them in small groups and to use their natural curiosity and herd movement [52]. Training of the personnel in good handling practices should help avoiding rough pig handling [53].

A certification of competence is obligatory for everyone who transports [7] or handles animals at the abattoir [8] and ensures a standardised level of knowledge among all personnel who are in contact with pigs. During transportation, a good driving style is important so that the pigs can maintain their body positions, and stress is reduced to the minimum [54]. Training programmes for personnel can minimise the use of electric prods [54]. In the event of rough or aggressive handling, personnel should undergo further training according to the instructions of the supervisor or should be excluded from working in the areas with live animals [54].

### 4.2. Ambient Noise

A quiet environment has positive effects on pig handling and pig welfare, because the animals are less stressed [51]. A maximum threshold could be 85 dB [43], as presented in the results section of this SLR. All unnecessary noises during the handling of pigs should be avoided [54]. Eliminating sources of loud noise if possible [53] and not shouting at the animals could be first interventions [51,53]. Van de Perre [55] also mentioned, that controlling the ambient noise could be possible if abattoirs were built with sound-isolating material.

### 4.3. Parameters in Blood, Saliva and Meat

Cortisol is a widely used stress marker for pigs [56]. Pre-slaughter stress can result in higher blood cortisol levels in pigs [57]. When measuring cortisol, it is important to take account of the time at which the stressor influenced the pig [58]. Becker et al. [59] showed that after different stimuli—e.g., new environment, electrical stimulation, heat stress—the serum cortisol levels of pigs peaked after 0.5–4.3 h. Other studies on snare restraining pigs and on transportation yielded the same results, meaning that shortly after the beginning of the stress and also after short transportation, cortisol levels increased [60,61,62]. In contrast, Brown et al. [63] observed significantly higher blood cortisol after a transport duration of 16 h (compared to 8 and 24 h) and no lairage, and a significant decrease of cortisol after journeys of 16 h and 24 h—compared to the control group. Piñeiro et al. [64] did not observe changes after transport times of 24 h or 48 h in pigs. On the one hand, this shows that short-term stress can result in high concentrations of cortisol, but on the other hand, this confirms that it is hard to trace back a high level of cortisol to a specific stressor. This makes it difficult to interpret blood cortisol as a general stress indicator because of the individual conditions pigs are exposed to on the day of slaughter.

Another stress parameter is blood lactate concentration, which could be easily measured with a handheld meter as shown for cows [65]. In pigs, blood lactate increased after the animals were snare restrained and decreased afterwards [60]. However, the increase, peak, and decrease—and the timing of all three—after the animal is exposed to multiple stressors, as occurs during the day of slaughter, could be quite different from the values measured in simpler studies. Many other stress parameters have been used in experimental studies, but these are not yet applicable at the abattoir during daily work, e.g., transport stress increased the number of polymorphic neutrophils and lymphocytes in blood [62]. Cerón et al. [56] discussed more stress parameters that are measurable in the pigs’ saliva, such as salivary alpha-amylase, chromogranin-a, total esterase activity, oxytocin, and immunoglobulin A. The focus on saliva samples could be a good approach under abattoir conditions in the search for new solutions to measuring stress in pigs.

Grandin [66] in her review said that stress directly before slaughter produces meat with a lower pH and, therefore, increases the incidence of PSE meat. Longer lasting stress produced darker coloured meat, and short or acute stress lighter meat [66]. Together with measuring the pH, the colour of the meat can also be a stress indicator, and while it is not that sensitive, it is connected to the meat pH. Negative influences on the meat pH are caused by poor acidification due to, for example, intense muscle activity shortly before death or after fatigue, strong excitement, and sick animals [67]. Therefore, calm handling prior to stunning is important, and stunning should cause as few muscle spasms and movements as possible [67].

### 4.4. Stocking Density during Transport

Stocking density during transport is regulated by Regulation (EC) No 1/2005 [7]. Pigs with live weight “around” 100 kg are allowed to be transported with a density of 235 kg/m^2^ [7]. In Germany, finishing pigs have a weight of about 110–120 kg, and, therefore, it is not clear how to transport these heavier finishing pigs in accordance with the European legislation. The national regulation in Germany enables groups of 15 fattening pigs with a weight of more than 70 kg each to be transported in one compartment [10]. More specific rules are also legislated for space allowance. If pigs of 110 or 120 kg are loaded, the transport density is 220 kg/m^2^ and 218 kg/m^2^, respectively, but if pigs of more than 120 kg are loaded 0.7 m^2^ for each pig must be provided [10]. This means more space for individual pigs, so, for example, with 130 kg pigs the transport density would be 185 kg/m^2^. More space on the truck can reduce the risk of heat stress in pigs, especially if they have no body contact [54]. This is in contrast to the results of this SLR, as more fights and body injuries were observed in transports with lower stocking densities [20,34]. Standing pigs or a sternal lying position for pigs with a body weight of 110–120 kg is possible if the transport density is about 250 kg/m^2^ [54]. Additional to this finding, the results regarding measured pH values in the studies above allow the assumption that the transport density as required by the European legislation could be appropriate. In contrast, semi-recumbent lying is only possible with more space allowance (about 180 kg/m^2^) [54]. This underlines the importance of calculating and checking the transport density for every batch of animals and transport procedure.

### 4.5. Transport Duration

Despite careful route planning, transport duration is a factor that is not always influenceable by drivers because of weather influences, traffic jams, and potential contracts between farmers or transport companies and abattoirs. Transport duration should be as short as possible, as can be read in Regulation (EC) No 1/2005 (Art. 3) [7]. Long-term transport of animals with a duration over 8 h should be reduced to a minimum [7]. Bozzo et al. [68] compared 3 h journeys with 11 h journeys, with regard to the measured stress hormones and in consensus with the results of this SLR, they stated that after a long transport time the animals should be allowed to stay longer in lairage. The unloading process should start as soon as possible after arriving at the abattoir [53].

### 4.6. Lairage and Duration of Lairage 

Stocking density during lairage in combination with good ventilation seem to influence the “dead-in-pen rate” and, therefore, animal protection [29]. The appropriate lairage time can be difficult to determine as contradictory results in this SLR were found, and, thus, should be individual for the batches and abattoirs [15,23]. The influence on meat quality was also considered when defining the time for lairage. Meat quality indicators can indirectly be traced back and, therefore, can be retrospective welfare indicators. In concordance to the results of this SLR [16,34,40], Čobanović et al. [69] showed that a higher proportion of PSE meat resulted after short lairage under 1 h and a higher proportion of DFD meat after overnight lairage. Some authors did not find differences in meat quality or similar incidences for developing PSE [15,16,18], which proves the difficulty of tracing back meat quality outcomes to a single stressor, and indicates the need for further research. As stated by the Animal Health and Welfare Panel of the European Food Safety Authority (EFSA) [53], lairage should be avoided or kept to a minimum length of time. This was also shown by the results of Driessen et al. [27], as good meat quality was measured after 2–165 min lairage duration. If mixing is unavoidable, more space during lairage and showering the pigs could have positive effects on pig welfare [53].

### 4.7. Stunning Procedure

Restraining pigs during head electrical stunning with walls or boards should be implemented as standard practice [53]. Training personnel on the correct placement of electrodes, electrical contact, electrical parameters, and exposure time is essential [53], which was also shown in our SLR results. For an efficient head cycle the electrodes must be placed on the ground of the ear on both sides of the head [70]. A heart cycle can be performed afterwards. During the heart cycle, the heart must be placed between the electrodes: side-to-side, back-to-chest or head-to-heart [70]. As also shown in the results of this SLR, personnel-related risk factors during stunning include bad or wrong positioning of electrical tongs, a lack of experience, and tiredness if manual electrical stunning is performed [71]. Even though it has been shown that electrical currents below 0.7 A have led to good stunning results [47], the current of 1.3 A specified by the EU legislation [8] must never be undercut. A rotating system for personnel working in this animal welfare-sensitive area can be positive for animal welfare [53]. The training of personnel performing CO_2_-stunning is essential, especially with regard to exposure time, gas concentration, loading of animals in the stunner, and gas temperature [53].

### 4.8. Control of Stunning Effectiveness

It is obligatory to ensure that pigs do not show signs of consciousness or sensibility after stunning until death (Council Regulation (EC) No 1099/2009 Article 5 [8]). EFSA recommend indicators to assess a pigs’ unconsciousness directly after stunning, during sticking, and during bleeding [71]. These indicators are in accordance with the findings in this current SLR and with the publication of Eyes on Animals [51]. After electrical stunning, the presence of typical tonic/clonic seizures can be seen, and these should also be visible during sticking [71]. The state of unconsciousness is in doubt if no tonic/clonic seizures are present [51]. Eye reflexes and rhythmic breathing should be absent [51,71]. In addition, corneal reflexes can be present directly after stunning, but should not be visible during bleeding [51]. Again, this shows the need to assess more than one parameter. Spontaneous blinking or following the movements of the eyes should not occur and this also could be checked by personnel [51]. During bleeding, pigs should not attempt to rise or lift the head [51,71]. Immediately after CO_2_-stunning and after bleeding when electrical stunning is performed, the loss of muscle tone is another indicator for unconsciousness [71], whereas regular kicking or movements indicate that the pig is regaining consciousness [51]. If during bleeding one pig shows a different body posture in comparison to the others, this pig must be further examined [51]. For both stunning methods, responses to painful stimuli or reactions to the sticking incision along with vocalisation, should also be in focus [51]. 

### 4.9. Management Related Factors

The slaughter line speed is of importance and can be regulated by management [71], depending on the number and skills of personnel. Another important management-based factor is part of the European legislation, according to which animals should be steadily supplied for stunning and killing, so that the personnel do not rush the animals from lairage [8].

### 4.10. Sticking and Bleeding

Efficient bleeding requires deep stunning [72]. It is essential that the personnel responsible for bleeding are well educated in this highly “animal welfare-relevant process” [72,73], especially knowledge of the stun-to-stick interval, the importance and usage of sharp, long knives and the correct position of the sticking incision [53]. Two carotid arteries or major vessels should be severed [8]. Knowledge about the correct sticking position is important and is well described for the hollow knife. The sticking incision is about 5 cm cranial to the sternum directed to the heart, whereafter the Vena cava and usually at least one artery is wounded [74]. Well-performed bleeding has a big impact on the amount of blood exiting the incision [73] and, therefore, influences the occurrence of death. The actual sticking technique can be an influencing factor because if a butcher’s knife is used in comparison to a hollow knife, the person using it needs more experience [73]. However, visual estimation of the amount of blood flowing from the body is easier when a butcher’s knife is used [74] because blood flow in the hollow knife is not visible [75]. The amount of blood flow in the first seconds after sticking can be indicative of whether death is very likely to occur or not [74] and is influenced by the person performing sticking [72,75]. Note that the mean amount of blood loss after sticking should be taken into account if the expertise of the sticking person is evaluated, but the percentage of pigs with blood loss of under 2.5 L is more important for animal welfare [75]. According to Troeger et al. [72], blood losses of 1.75% of the total body weight should be reached and, in addition, 1.5% should have left the body within the first 10 s after sticking. Another important factor is the stun-to-stick interval. In order not to regain consciousness after stunning, the time span until sticking must be performed is defined in the German legislation for the different stunning systems [9]. After electrical stunning, the initiation of bleeding must happen within 10 s if the pigs are lying and 20 s if the pigs are shackled and hanging [9]. If CO_2_-stunning is applied, sticking must occur within 20 s after the pigs leave the stunner or 30 s after the last stop in the CO_2_ atmosphere [9]. However, the data collected on stun-to-stick intervals in this SLR have shown that even with a much longer delay until the sticking incision is made, stunning and deep unconsciousness can be ensured. In spite of that, a longer interval should stringently be avoided from the animal welfare and legal points of view. A “back up stunning procedure” must be always available [8] and applied if pigs show signs of consciousness or doubtful unconsciousness after stunning and during bleeding until they are dead. 

### 4.11. Additional Information and Limitations 

Numerous institutions have worked on handouts and recommendations in order to ensure animal welfare during the day of slaughter, including transport procedures. These elaborate guides and scientific publications provide a good basis to recapitulate the principles of animal welfare for pigs so that trained personnel apply best practice in their day-to-day work [51,53,54,71]. 

In general, it must be taken into account that the studies in the results section had different approaches regarding the consideration of the different changes on the animal-based measurements or indicators. A possible potentiation of animal welfare-related issues or also a weakening in the course of the slaughter day can take place and must be considered.

By defining the inclusion and exclusion criteria, it can be assumed that important non-European or older literature did not appear in the results section of this SLR. Especially the definition of the publication period, study location, and article type can, therefore, be seen as a limitation. Nevertheless, it can be assumed that the defined search and inclusion criteria and, thus, the results of the relevant publications reflect the current state of scientific knowledge in the context of European legislation.

## 5. Conclusions

This SLR has shown that especially handling and human-animal interaction during the transport and slaughter of pigs can influence animal welfare and stress in pigs. Together with improved management on the day of slaughter the sufficient training of personnel is crucial. An excellent repetitive training of handling practice and the animal welfare-sensitive process steps can set a good standard at abattoirs. Freely available summaries for the day-to-day transport, abattoir practice, and scientific opinions regarding transport and slaughter can form the basis for finding and improving critical control points individually for each slaughter plant and to ensure pig welfare. 

## Figures and Tables

**Table 1:** Inclusion and exclusion criteria for the systematic literature review on animal welfare of pigs during transport and slaughter.

Parameter	Inclusion Criteria	Exclusion Criteria
study/article type	original article published in peer-reviewed journal	reviews, systematic reviews;journals/papers published without peer-review process
publication period	2009–2022	before 2009
study location	Europe; countries applying European law	all other countries
animals included	studies on fattening pigs	studies on sows, boars, piglets
topic animal welfare	information on animal welfare at transport and/or slaughter + information on management-based indicators and/or animal-based indicators	information on animal welfare at farm + information not including management-based and animal-based indicators
transport duration	short journey	long journey (>8 h) ^1^

^1^ as defined in Reg. (EC) No 1/2005 [7].

## Data Availability

Data sharing not applicable.

## Supplementary Materials

The following supporting information can be downloaded at: https://www.mdpi.com/article/10.3390/ani12233391/s1, S1: PRISMA 2020 Checklist for Isbrandt et al. “Impact of Procedures and Human-Animal Interactions during Transport and Slaughter on Animal Welfare of Pigs: A Systematic Literature Review”; S2: Detailed search protocol; S3: List of all records; S4: List of included publications
