# Supplementary material for: Impact of Procedures and Human-Animal Interactions during Transport and Slaughter on Animal Welfare of Pigs: A Systematic Literature Review
Source: Animals (Basel). 2022 Dec 2;12(23):3391. doi: 10.3390/ani12233391 (PMC9740978; doi:10.3390/ani12233391)
Supplement: Supplementary file 1 [file animals-12-03391-s001.zip › S2 Detailed search protocol.pdf]

Impact of Procedures and Human-Animal Interactions during Transport and Slaughter on Animal Welfare of Pigs: A Systematic Literature Review

S2: Detailed search protocol

Search terms and search term combinations used in all of the three following databases:

- (pig OR swine) AND (animal welfare OR welfare) AND (transport)
  - (Schwein OR Mastschwein) AND (Tierwohl OR Tierschutz) AND (Lebendtiertransport OR Tiertransport OR Viehtransport OR Transport)
- (pig OR swine) AND (animal welfare OR welfare) AND (slaughter OR slaughterhouse OR abattoir OR lairage OR bleeding OR stunning)
  - (Schwein OR Mastschwein) AND (Tierwohl OR Tierschutz) AND (Schlachtung OR Schlachthaus OR Schlachtbetrieb OR schlachten OR Schlachthof OR Tötung OR Betäubung OR Entblutung OR Wartestall).

Setting during search in the database Pubmed®
https://pubmed.ncbi.nlm.nih.gov/

First Search: 22.07.2020 + 23.07.2020

Article Type: Case Reports; Classical Article; Clinical Study; Clinical Trial Protocol; Clinical Trial, Phase I; Clinical Trial, Phase II; Clinical Trial, Phase III; Clinical Trial, Phase IV; Clinical Trial, Veterinary; Comparative Study; Controlled Clinical Trial; Corrected and Republished Article; Dataset; English Abstract; Evaluation Study; Government Publication; Guideline; Interactive Tutorial; Introductory Journal Article; Journal Article; Legislation; Multicenter Study; Observational Study; Observational Study, Veterinary; Practice Guideline; Pragmatic Clinical Trial; Preprint; Twin Study; Validation Study

Publication Years: 2009- date of search

Repeated Search: 28.01.2022

Duplicates from 2020 were subsequently removed

Article Type: Case Reports; Classical Article; Clinical Study; Clinical Trial Protocol; Clinical Trial, Phase I; Clinical Trial, Phase II; Clinical Trial, Phase III; Clinical Trial, Phase IV; Clinical Trial, Veterinary; Comparative Study; Controlled Clinical Trial; Corrected and Republished Article; Dataset; English Abstract; Evaluation Study; Government Publication; Guideline; Interactive Tutorial; Introductory Journal Article; Journal Article; Legislation; Multicenter Study; Observational Study; Observational Study, Veterinary; Practice Guideline; Pragmatic Clinical Trial; Preprint; Twin Study; Validation Study

Publication Years: 2020- date of search

Setting during search in the database Livivo
https://www.livivo.de/

First Search: 22.07.2020

Year: of 2009 until date of search
Document Types: Article
Language: German or English

Repeated Search: 28.01.2022
Duplicates from 2020 were subsequently removed

Year: of 2020 until date of search
Document Types: Article
Language: German or English

Setting during search in the database Web of Science™
https://www.webofscience.com/

First Search: 23.07.2020

Document Types: Article; Review
Publication Years: 2009-date of search

Repeated Search: 28.01.2022
Duplicates from 2020 were subsequently removed.

Document Types: Article
Publication Years: 2020 or 2021 or 2022
