# Supplementary material for: Impact of Procedures and Human-Animal Interactions during Transport and Slaughter on Animal Welfare of Pigs: A Systematic Literature Review
Source: Animals (Basel). 2022 Dec 2;12(23):3391. doi: 10.3390/ani12233391 (PMC9740978; doi:10.3390/ani12233391)
Supplement: Supplementary file 1 [file animals-12-03391-s001.zip › S3 List of all records.pdf]

Impact of Procedures and Human-Animal Interactions during Transport and Slaughter on Animal Welfare of Pigs:
A Systematic Literature Review

S3: List of all records

Hits 1.-883. found during first search (22-23.07.2020)
Hits 884.-1.099. found after repeated search (including comparison to first search) (28.01.2022)
after literature search in the Databases Pubmed®, Web of Science™ and LIVIVO

	Authors	Year	Title	Journal	DOI
1.	S. Einarsson; Y. Sjunnesson; F. Hultén; L. Eliasson-Selling; A.-M. Dalin; N. Lundeheim; U. Magnusson	2014	A 25 years experience of group-housed sows-reproduction in animal welfare-friendly systems	Acta veterinaria Scandinavica	
2.	A. K. Johnson; L. M. Gesing; M. Ellis; J. J. McGlone; E. Berg; S. M. Lonergan; R. Fitzgerald; L. A. Karriker; A. Ramirez; K. J. Stalder; A. Sapkota; R. Kephart; J. T. Selsby; L. J. Sadler; M. J. Ritter	2013	2011 and 2012 Early Careers Achievement Awards: farm and pig factors affecting welfare during the marketing process	J Anim Sci	10.2527/jas.2012-6114
3.	R. P. Smith; M. J. Sanchez-Vazquez; A. J. C. Cook; S. A. Edwards	2011	Abattoir-based study investigating the association between gross pathological lesions and serological tests for Salmonella infection in pigs	The Veterinary record	
4.	D. Peskovicova; P. Demo; M. Oravcova; I. Bahelka; D. Vasicek	2009	Achievements of research in the field of swine	Animal Production and Animal Science Worldwide: Waap Book of the Year 2007: A Review on Developments and Research in Livestock Systems	
5.	J. Gomez-Laguna; F. J. Salguero; F. J. Pallares; I. M. Rodriguez-Gomez; I. Barranco; L. Carrasco	2011	Acute Phase Proteins as Biomarkers in Animal Health and Welfare	Acute Phase Proteins as Early Non-Specific Biomarkers of Human and Veterinary Diseases	
6.	A. M. Haberland; J. Dodenhoff; R. Eisenreich; K. U. Gotz	2017	Adventitious bursitis in Bavarian fattening pigs housed conventionally	Zuchtungskunde	
7.	F. Sauer; F. Schmoll; T. Sattler	2014	Age at second Improvac((R))-vaccination and its influence on testis weight, testis histology and boar taint compounds of male fatteners compared to entire boars and barrows	Wiener Tierarztliche Monatsschrift	
8.	P. Rolzhauser; J. Saffaf; S. Wohlfahrt; L. Walter; A. Pahl; A. Hamedy; E. Lucker; K. Riehn	2020	The age determination of porcine fetuses against the background of illegal delivery of highly pregnant mammals for slaughtering	Berliner Und Munchener Tierarztliche Wochenschrift	10.2376/0005-9366-18075
9.	B. Bünger; L. Schrader; H. Schrade; B. Zacharias	2015	Agonistic behaviour, skin lesions and activity pattern of entire male, female and castrated male finishing pigs		

10.	B. Bünger; B. Zacharias; P. Grün; E. Tholen; H. Schrade	2011	Agonistisches Verhalten von nicht kastrierten männlichen, weiblichen und kastrierten männlichen Mastschweinen unter LPA-Standard		
11.	W. Zheng; Y. Zhao; H. Xin; R. S. Gates; B. Li; Y. Zhang; M. L. Soupir	2014	AIRBORNE PARTICULATE MATTER AND CULTURABLE BACTERIA REDUCTION FROM SPRAYING SLIGHTLY ACIDIC ELECTROLYZED WATER IN AN EXPERIMENTAL AVIARY LAYING-HEN HOUSING CHAMBER	Transactions of the Asabe	
12.		2015	Aktuell. Bei der Initiative Tierwohl ist die erste Registrierungsphase für schweinehaltende Betriebe abgeschlossen	Fleischwirtschaft	
13.	M. Marahrens; I. Schwarzlose; J. Knöll; A. Biermann; D. Möhrlein; J. Hartung	2020	Aktuelle Untersuchungen zum Tierschutz bei der Betäubung von Schlachtschweinen und Rindern<br>[Keynote]		
14.	M. Machtolf	2012	Aktuelles aus der internationalen Fleischforschung<br>Fettsubstitution von Hackfleischerzeugnissen mit Surimi, Optimierung der Zartheit von ökologisch erzeugtem Schweinefleisch, Aspekte des Tierschutzes bei Schlachtschweinen		
15.	A. Foury; B. Lebret; P. Chevillon; A. Vautier; C. Terlouw; P. Mormède	2011	Alternative rearing systems in pigs: consequences on stress indicators at slaughter and meat quality	Animal	10.1017/s1751731111000784
16.		2016	Alternativen der Reduzierung und Eliminierung von Ebergeruch neben der chirurgischen Kastration. Bei der diesjährigen 129. Fortbildungstagung der Landesarbeitsgemeinschaft Fleischhygiene und Tierschutz Bayern am 12.5.2016 in Kempten stand das Thema Tierwohl in der Fleischwirtschaft und insbesondere in der Schweinefleischproduktion auf der Tagesordnung. In diesem Rahmen wurden die Ergebnisse der Dissertation von Dr. Diana Engesser zum Thema Alternativen der Reduzierung und Eliminierung von Ebergeruch neben der chirurgischen Kastration vorgestellt	Rundschau für Fleischhygiene und Lebensmittelüberwachung	
17.	C. Veit; M. Marahrens; I. Schwarzlose; E. T. Krause; L. Schrader	2017	Alternativen zur betäubungslosen Ferkelkastration in Deutschland<br>Überblick zum aktuellen Stand der Forschung ; Alternatives to anesthesia-free piglet castration in Germany: Overview of the current state of research		
18.	F. W. Bazer; G. A. Johnson; G. Wu	2015	Amino acids and conceptus development during the peri-implantation period of pregnancy	Advances in experimental medicine and biology	
19.	S. Starosta	2015	Amtliche Schlachthofbefunde als Datengrundlage für ein Tierwohl-Monitoring<br>Potentiale und Grenzen		
20.	A. Vitali; E. Lana; M. Amadori; U. Bernabucci; A. Nardone; N. Lacetera	2014	Analysis of factors associated with mortality of heavy slaughter pigs during transport and lairage	J Anim Sci	10.2527/jas.2014-7670

21.	S. Muszyński; S. Świątkiewicz; A. Arczewska-Włosek; P. Dobrowolski; J. L. Valverde Piedra; M. B. Arciszewski; S. Szymańczyk; A. Zacharko-Siembida; S. Kowalik; M. Hułas-Stasiak; A. Tomczyk-Warunek; T. Schwarz; E. Tomaszewska	2019	Analysis of mechanical properties of bones and tendons shows that modern hybrid rye can be introduced to corn-wheat based diet in broiler chickens as an alternative energy source irrespective of xylanase supplementation	Poult Sci	10.3382/ps/pez323
22.	J. Wasilewski; M. Szczepanik; Z. Burski; S. Juscinski	2020	Analysis of Non-conformities in Road Transport of Animals in Poland	Journal of Applied Animal Welfare Science	10.1080/10888705.2019.1668790
23.	G. Bozzo; R. Barrasso; P. Marchetti; R. Roma; G. Samoilis; G. Tantillo; E. Ceci	2018	Analysis of Stress Indicators for Evaluation of Animal Welfare and Meat Quality in Traditional and Jewish Slaughtering	Animals	10.3390/ani8040043
24.	J. Tröscher-Mußotter; B. Tilocca; V. Stefanski; J. Seifert	2019	Analysis of the Bacterial and Host Proteins along and across the Porcine Gastrointestinal Tract	Proteomes	10.3390/proteomes7010004
25.	S. Azarpajouh; J. Marchewka; J. C. Segura Correa; J. A. Calderón Díaz	2018	Anatomical characterization of hoof growth pattern in six Iranian sheep breeds and its possible implication for trimming recommendations	Trop Anim Health Prod	10.1007/s11250-018-1566-y
26.	F. Wirths	2011	Anforderungen des Tierschutzes an Transport und Schlachtung von Bio-Schweinen	Praxis trifft Forschung : neues aus dem ökologischen Ackerbau und der ökologischen Tierhaltung 2011	
27.	J. Hultgren; K. A. Segerkvist; C. Berg; A. H. Karlsson; B. Algers	2020	Animal handling and stress-related behaviour at mobile slaughter of cattle	Preventive Veterinary Medicine	10.1016/j.prevetmed.2020.104959
28.	K. Lindgren	2014	Animal health and welfare in production systems for organic fattening pigs	Organic agriculture. 2014 June, v. 4, no. 2	
29.	M. Xavier; S. Caio Abércio da; B. Ana Maria; D. Cleandro Pazinato	2014	Animal welfare<br>concepts and practical procedures to evaluate the swine productions systems	Semina: Ciências Agrárias, Vol 34, Iss 6Supl2, Pp 4213-	
30.	I. Deimel; A. Franz; A. Spiller	2012	Animal Welfare<br>eine empirische Analyse landwirtschaftlicher Frames ; Animal Welfare: An Empirical Analysis of Agricultural Frames		
31.	M. A. Gerritzen; M. A. B. Raj	2009	Animal welfare and killing for disease control	Welfare of Production Animals: Assessment and Management of Risks	
32.	J. Hartung; B. Nowak; A. C. Springorum	2009	Animal welfare and meat quality	Improving the Sensory and Nutritional Quality of Fresh Meat	10.1533/9781845695439.4.628
33.	T. Grandin	2014	Animal welfare and society concerns finding the missing link	Meat Science	10.1016/j.meatsci.2014.05.011
34.	J. Hartung; A. C. Springorum	2009	Animal welfare and transport	Welfare of Production Animals: Assessment and Management of Risks	

35.	S. More; D. Bicout; A. Botner; A. Butterworth; P. Calistri; K. Depner; S. Edwards; B. Garin-Bastuji; M. Good; C. Gortazar Schmidt; V. Michel; M. A. Miranda; S. Saxmose Nielsen; A. Velarde; H.-H. Thulke; L. Sihvonen; H. Spoolder; J. A. Stegeman; M. Raj; P. Willeberg; D. Candiani; C. Winckler	2017	Animal welfare aspects in respect of the slaughter or killing of pregnant livestock animals (cattle, pigs, sheep, goats, horses)	EFSA journal. European Food Safety Authority	
36.	A. Velarde; A. Dalmau	2012	Animal welfare assessment at slaughter in Europe: moving from inputs to outputs	Meat Sci	10.1016/j.meatsci.2012.04.009
37.	R. Stocchi; N. A. Mandolini; M. Marinsalti; N. Cammertoni; A. R. Loschi; S. Rea	2014	Animal Welfare Evaluation at a Slaughterhouse for Heavy Pigs Intended for Processing	Ital J Food Saf	10.4081/ijfs.2014.1712
38.	M. Medina-Vara; I. Guerrero-Legarreta; P. Roldan-Santiago; P. Castaneda-Serrano; M. Gonzalez-Lozano; D. Mota-Rojas	2016	ANIMAL WELFARE IN BROILERS: PHYSIOLOGICAL PROFILES IN RESPONSE TO TRANSPORT TO ABATTOIR, LAIRAGE AND EXSANGUINATION	Revista Cientifica-Facultad De Ciencias Veterinarias	
39.	P. Roldan-Santiago; D. Mota-Rojas; I. Guerreo-Legarreta; P. Mora-Medina; F. Borderas-Tordesillas; A. D. Alarcon-Rojo; S. Flores-Peinado; H. Orozco-Gregorio; R. Martinez-Rodriguez; M. E. Trujillo-Ortega	2013	Animal welfare of barrows with different antemortem lairage times without food	Veterinarni Medicina	10.17221/6866-vetmed
40.	A. Velarde; E. Fàbrega; I. Blanco-Penedo; A. Dalmau	2015	Animal welfare towards sustainability in pork meat production	Meat Sci	10.1016/j.meatsci.2015.05.010
41.	A. M. Maisano; M. Luini; N. Vitale; S. Rota Nodari; F. Scali; G. L. Alborali; F. Vezzoli	2019	Animal-based measures on fattening heavy pigs at the slaughterhouse and the association with animal welfare at the farm level: a preliminary study	Animal : an international journal of animal bioscience	
42.	T. Sattler; F. Schmoll	2012	An animal-friendly alternative to surgical castration Haptoglobin in boars vaccinated with Improvac (R) and surgical castrated boars as well as carcass and meat quality in comparison to entire boars	Fleischwirtschaft	
43.	M. Nather; L. Theuvsen	2015	Animal-welfare friendly control of epizootics does not have to be expensive - An analysis using the example of classical swine fever	Tieraerztliche Umschau	
44.	A. B. Kruse; C. S. Kristensen; U. Lavlund; H. Stege	2019	Antimicrobial prescription data in Danish national database validated against treatment records in organic pig farms and analysed for associations with lesions found at slaughter	BMC Vet Res	10.1186/s12917-019-1913-x
45.	I. Žaja; M. Samardžija; S. Vince; A. Sluganović; S. Strelec; J. Šuran; I. DelVechio; D. Đuričić; M. Ostović; H. Valpotić; S. Milinković-Tur	2016	Antioxidant protection and lipid peroxidation in testes and different parts of epididymis in boars	Theriogenology	10.1016/j.theriogenology.2016.07.008
46.	M. Sönnichsen	2011	Anwendung des Welfare Quality® Protokolls zur Bewertung des Tierwohls in der Schweineproduktion in Spanien	Mitteilungsblatt Fleischforschung Kulmbach	
47.	S. S. Nielsen; A. M. Michelsen; H. E. Jensen; K. Barington; K. V. Opstrup; J. F. Agger	2014	The apparent prevalence of skin lesions suspected to be human-inflicted in Danish finishing pigs at slaughter	Prev Vet Med	10.1016/j.prevetmed.2014.08.003
48.	T. L. Pereira; E. A. L. Titto; S. Conte; N. Devillers; R. Sommavilla; T. Diesel; F. A. Dalla Costa; F. Guay; R. Friendship; T. Crowe; L. Faucitano	2018	Application of a ventilation fan-misting bank on pigs kept in a stationary trailer before unloading: Effects on trailer microclimate, and pig behaviour and physiological response	Livestock Science	10.1016/j.livsci.2018.07.013

49.	A. M. Gutierrez; M. I. Villa; B. A. Marsilla; S. Martinez-Subiela; A. M. Montes; J. J. Ceron	2015	Application of acute phase protein measurements in meat extract collected during routine veterinary inspection at abattoirs	Research in Veterinary Science	10.1016/j.rvsc.2015.05.019
50.	L. Faucitano; S. Conte; C. Pomar; D. Paiano; Y. Duan; P. F. Zhang; G. Drouin; S. Rina; F. Guay; N. Devillers	2020	Application of extended feed withdrawal time preslaughter and its effects on animal welfare and carcass and meat quality of enriched-housed pigs	Meat Science	10.1016/j.meatsci.2020.108163
51.	A. Gavinelli; T. Kennedy; D. Simonin	2014	The application of humane slaughterhouse practices to large-scale culling	Rev Sci Tech	10.20506/rst.33.1.2280
52.	S. Akase; Y. Uchitani; Y. Sohmura; K. Tatsuta; K. Sadamasu; Y. Adachi	2009	Application of real time PCR for diagnosis of Swine Dysentery	J Vet Med Sci	10.1292/jvms.71.359
53.	A. Dalmau; D. Tample; P. Rodriguez; P. Llonch; A. Velarde	2009	Application of the Welfare Quality (R) protocol at pig slaughterhouses	Animal Welfare	
54.	A. Dalmau; A. Nande; M. Vieira-Pinto; S. Zamprogna; G. Di Martino; J. C. R. Ribas; M. P. da Costa; K. Halinen-Elemo; A. Velarde	2016	Application of the Welfare Quality protocol in pig slaughterhouses of five countries	Livestock Science	10.1016/j.livsci.2016.10.001
55.	C. Pomar; L. Hauschild; G.-H. Zhang; J. Pomar; P. A. Lovatto	2009	Applying precision feeding techniques in growing-finishing pig operations	Revista Brasileira de Zootecnia	
56.	A. Gizella; D. O. Stefania; T. Francesco; Z. Martina; C. Salvatore; C. Leonardo Nanni	2018	Apulo-Calabrese and Crossbreed Pigs Show Different Physiological Response and Meat Quality Traits after Short Distance Transport	Animals, Vol 8, Iss 10, p	
57.		2017	Arzneimittelrecht, Tierwohl und gesündere Tiere - Bericht von der Schweinefachtagung von Boehringer Ingelheim	Tierärztliche Umschau	
58.	D. Sueli Blanes; C. Roberto Souza; C. Rober; F. Luiz Francisco Poli de	2010	Aspectos fundamentais da experimentação animal - aplicações em cirurgia experimental Fundamental aspects on animal research as applied to experimental surgery	Revista da Associação Médica Brasileira, Vol 56, Iss 1, Pp 103-	
59.	D. Kümmerlen; S. Hartmann; A. Riklin; R. Figi; X. Sidler	2019	[Aspects of animal health, animal welfare and biosecurity during 101 transports of piglets in Switzerland]	Schweiz Arch Tierheilkd	10.17236/sat00198
60.	I. Böhne; T. große Beilage; I. Gerhauser; M. Hewicker-Trautwein; P. Wolf; J. Kamphues; E. große Beilage; H. Nathues	2010	Aspekte des Tierschutzes und der Lebensmittel-sicherheit im Fall einer Selen-Überdosierung im Mischfutter für Mastschweine	Tierärztliche Praxis G: Großtiere/Nutztiere	
61.	K. M. Knage-Rasmussen; T. Rousing; J. T. Sørensen; H. Houe	2015	Assessing animal welfare in sow herds using data on meat inspection, medication and mortality	Animal	10.1017/s1751731114002705
62.	E. E. Wigham; A. Butterworth; S. Wotton	2018	Assessing cattle welfare at slaughter - Why is it important and what challenges are faced?	Meat Science	10.1016/j.meatsci.2018.06.010
63.	U. Latacz_Lohmann	2019	Assessing Consumer and Producer Preferences for Animal Welfare Using a Common Elicitation Format	Journal of agricultural economics. 2019 June, v. 70, no. 2	
64.	S. R. Nodari; A. Polloni; S. Giacomelli; F. Vezzoli; G. Galletti	2014	Assessing pig welfare at stunning in Northern Italy commercial abattoirs using electrical method	Large Animal Review	
65.	S. Atkinson; A. Velarde; P. Llonch; B. Algers	2012	Assessing pig welfare at stunning in Swedish commercial abattoirs using CO2 group-stun methods	Animal Welfare	10.7120/09627286.21.4.487
66.	P. Rodriguez; A. Dalmau; X. Manteca; H. Litvan; E. W. Jensen; A. Velarde	2016	Assessment of aversion and unconsciousness during exposure to carbon dioxide at high concentration in lambs	Animal Welfare	10.7120/09627286.25.1.073
67.	P. Llonch; P. Rodríguez; M. Jospin; A. Dalmau; X. Manteca; A. Velarde	2013	Assessment of unconsciousness in pigs during exposure to nitrogen and carbon dioxide mixtures	Animal	10.1017/s1751731112001966

68.	S. Raasch; M. Postma; J. Dewulf; K. D. C. Stärk; E. g. Beilage	2018	Association between antimicrobial usage, biosecurity measures as well as farm performance in German farrow-to-finish farms	Porcine Health Management, Vol 4, Iss 1, Pp 1-	10.1186/s40813-018-0106-5
69.	A. R. Vieira	2009	Association Between Tetracycline Consumption and Tetracycline Resistance in Escherichia coli from Healthy Danish Slaughter Pigs	Foodborne pathogens and disease. 2009 Jan-Feb, v. 6, no. 1	
70.	I. Alpigiani; C. Bacci; L. J. Keeling; M. D. Salman; F. Brindani; S. Pongolini; P. L. Hitchens; S. Bonardi	2016	The associations between animal-based welfare measures and the presence of indicators of food safety in finishing pigs	Animal Welfare	10.7120/09627286.25.3.355
71.	M. Fertner; M. Denwood; A. C. Birkegård; H. Stege; A. Boklund	2017	Associations between Antibacterial Treatment and the Prevalence of Tail-Biting-Related Sequelae in Danish Finishers at Slaughter	Front Vet Sci	10.3389/fvets.2017.00182
72.	J. R. Pluske; D. W. Miller; S. O. Sterndale; D. L. Turpin	2019	Associations between gastrointestinal-tract function and the stress response after weaning in pigs	Animal Production Science	10.1071/an19279
73.	T. Grandin	2012	Auditing animal welfare and making practical improvements in beef-, pork- and sheep-slaughter plants	Animal Welfare	10.7120/096272812x13353700593400
74.	T. Grandin	2010	Auditing animal welfare at slaughter plants	Meat Science	10.1016/j.meatsci.2010.04.022
75.	S. Kruse	2014	Aufbau eines marktgerechten Tierwohlprogramms in der Schweinefleischkette_ (Tierwohl) ; Establishing a market-conform animal welfare program for the pig production chain		
76.	B. Bünger; B. Zacharias; H. Schrade	2015	Aufreiten bei Schweinen _ ein ausschließlich sexuell motiviertes Verhalten am Ende der Ebermast? ; Mounting by pigs _ only a sexual motivated behavior of entire boars at the end of fattening?		
77.	P. M. Thornber	2010	An Australian perspective on developing standards and ensuring compliance	J Vet Med Educ	10.3138/jvme.37.1.22
78.	A. Nasirahmadi; O. Hensel; S. A. Edwards; B. Sturm	2016	Automatic detection of mounting behaviours among pigs using image analysis	Computers and Electronics in Agriculture	10.1016/j.compag.2016.04.022
79.	R. B. D'Eath; M. Jack; A. Futro; D. Talbott; Q. M. Zhu; D. Barclay; E. M. Baxter	2018	Automatic early warning of tail biting in pigs: 3D cameras can detect lowered tail posture before an outbreak	Plos One	10.1371/journal.pone.0194524
80.	M. Kashiha; C. Bahr; S. A. Haredasht; S. Ott; C. P. H. Moons; T. A. Niewold; F. O. Odberg; D. Berckmans	2013	The automatic monitoring of pigs water use by cameras	Computers and Electronics in Agriculture	10.1016/j.compag.2012.09.015
81.	P. Llonch; A. Dalmau; P. Rodriguez; X. Manteca; A. Velarde	2012	Aversion to nitrogen and carbon dioxide mixtures for stunning pigs	Animal Welfare	10.7120/096272812799129475
82.	P. Llonch; P. Rodriguez; A. Velarde; V. A. de Lima; A. Dalmau	2012	Aversion to the inhalation of nitrogen and carbon dioxide mixtures compared to high concentrations of carbon dioxide for stunning rabbits	Animal Welfare	10.7120/096272812x13353700593923
83.	C. J. Phillips; M. K. Pines; T. Muller	2012	The avoidance of ammonia by sheep	Journal of Veterinary Behavior-Clinical Applications and Research	10.1016/j.jveb.2011.05.027

84.	C. Rüben; C. Visscher	2016	Bedarfsgerechte Fütterung und Tierschutz beim Schwein	Amtstierärztlicher Dienst und Lebensmittelkontrolle	
85.	K. Guay; G. Salgado; G. Thompson; B. Backus; A. Sapkota; W. Chaya; J. J. McGlone	2013	Behavior and handling of physically and immunologically castrated market pigs on farm and going to market	J Anim Sci	10.2527/jas.2012-5726
86.	V. Vanheukelom; S. Van Beirendonck; J. Van Thielen; B. Driessen	2012	Behavior, production results and meat quality of intact boars and gilts housed in unmixed groups: A comparative study	Applied Animal Behaviour Science	10.1016/j.applanim.2012.10.004
87.	J. L. Williams; B. T. Richert; J. N. Marchant-Forde; S. D. Eicher	2012	Behavioral changes in neonatal swine after an 8-hour rest during prolonged transportation	J Anim Sci	10.2527/jas.2011-4597
88.	M. J. Hotzel; E. J. C. Lopez; P. A. V. de Oliveira; A. L. Guidoni	2009	Behaviour and performance of pigs finished on deep bedding with wood shavings or rice husks in summer	Animal Welfare	
89.	L. Charli Beatriz; C. Osmar Antonio Dalla; R. Roberto de Oliveira; S. Expedito Tadeu Facco; A. Natália Bortoleto; A. Aurélia Pereira de; J. Amilton de Mello; A. Natália Chinellato de	2012	Bem-estar animal no manejo pré-abate e a influência na qualidade da carne suína e nos parâmetros fisilógicos do estresse Animal welfare at pre-slaughter handling and the influence on pork meat quality and on stress physiological parameters	Ciência Rural, Vol 42, Iss 3, Pp 532-	
90.	S. Carlos A. De P; N. Irenilza De A; S. Douglas D	2009	BEM-ESTAR DO TRABALHADOR EM ESPAÇOS CONFINADOS DE PRODUÇÃO ANIMAL / THE WELFARE OF WORKERS IN ANIMAL HOUSING	Revista Brasileira de Engenharia de Biossistemas, Vol 3, Iss 3, Pp 237-	
91.	M. Vanderzwalmen; P. Carey; D. Snellgrove; K. A. Sloman	2020	Benefits of enrichment on the behaviour of ornamental fishes during commercial transport	Aquaculture	10.1016/j.aquaculture.2020.735360
92.		2019	Betriebszweig Schwein. Branche. Stallbau und Tierwohl	DLG-Mitteilungen	
93.		2019	Betriebszweig Schwein. In Kürze. Tierwohl-Label, Exportmärkte	DLG-Mitteilungen	
94.		2019	Betriebszweig Schwein. Tierwohlindikator. Das verrät die Schwanzhaltung	DLG-Mitteilungen	
95.	M. Salines; M. Andraud; N. Rose; S. Widgren	2020	A between-herd data-driven stochastic model to explore the spatio-temporal spread of hepatitis E virus in the French pig production network	PLoS One	10.1371/journal.pone.0230257
96.	C. Palmer; H. G. Pedersen; P. Sandoe	2018	Beyond Castration and Culling: Should We Use Non-surgical, Pharmacological Methods to Control the Sexual Behavior and Reproduction of Animals?	Journal of Agricultural & Environmental Ethics	10.1007/s10806-018-9718-7
97.	M. Povod; I. Lozynska; E. Samokhina	2019	Biological and economic aspects of immunological castration in comparison with traditional (surgical) method	Bulgarian Journal of Agricultural Science	
98.	S. Buncic; J. D. Collins; F. J. M. Smulders; P. Colin	2009	Biological food safety in relation to animal welfare	Welfare of Production Animals: Assessment and Management of Risks	
99.	M. Heimann	2009	Blood collection from the sublingual vein in mice and hamsters: a suitable alternative to retrobulbar technique that provides large volumes and minimizes tissue damage	Laboratory animals. 2009 July, v. 43, no. 3	
100.	R. Fries; G. Rindermann; C. Siegling-Vlitakis; N. Bandick; L. Bräutigam; A. Buschulte; H. Irsigler; K. Wolf; H. Hartmann	2013	Blood parameters and corneal-reflex of finishing pigs with and without lung affections observed post mortem in two abattoirs stunning with CO_	Research in veterinary science	
101.	L. Reed; J. L. MacNicol; A. Charchoglyan; D. Brewer; C. Murrant; W. Pearson	2019	A Botanical-Based Equine Nutraceutical Reduces Gastric Smooth Muscle Contractile Force In Vitro	Journal of equine veterinary science	

102.	R. Dwinger; B. Lambooij	2012	A brief summary of European legislation regarding animal welfare	Berl Munch Tierarztl Wochenschr	
103.	M. J. Sanchez-Vazquez; W. D. Strachan; D. Armstrong; M. Nielen; G. J. Gunn	2011	The British pig health schemes: integrated systems for large-scale pig abattoir lesion monitoring	Vet Rec	10.1136/vr.d4814
104.	R. B. T. R. d. Silva; I. d. A. Nääs; D. J. d. Moura	2009	Broiler and swine production: animal welfare legislation scenario	Scientia Agricola	
105.	F. Tuyttens; F. Vanhonacker; W. Verbeke	2014	Broiler production in Flanders, Belgium: current situation and producers' opinions about animal welfare	Worlds Poultry Science Journal	10.1017/s004393391400035x
106.	M. Krüger; N. Zinne; H. Höffler; R. Zhang; I. Kropivnitskaja; J. Schmitto; A. Ciubotaru; A. Haverich	2013	Broncho-/tracheoplastische Ex-situ-Operationen im organ care system""	Der Chirurg; Zeitschrift fur alle Gebiete der operativen Medizen	
107.		2011	BVA calls for more investigation of welfare abuse at abattoir	Vet Rec	10.1136/vr.d5086
108.	M. Kölln; A. Loi-Brügger; J. Kamphues	2017	A by-product of swine slaughtering as a protein source in broiler diets: effects on performance, composition of excreta, litter quality and on foot pad health	J Anim Physiol Anim Nutr (Berl)	10.1111/jpn.12668
109.	I. Kempf; A. Kerouanton; S. Bougeard; B. Nagard; V. Rose; G. Mourand; J. Österberg; B. O. Bengtsson	2017	Campylobacter coli in Organic and Conventional Pig Production in France and Sweden<br>Prevalence and Antimicrobial Resistance		
110.	V. Deiss; D. Temple; S. Ligout; C. Racine; J. Bouix; C. Terlouw; A. Boissy	2009	Can emotional reactivity predict stress responses at slaughter in sheep?	Applied Animal Behaviour Science	10.1016/j.applanim.2009.03.018
111.	M. L. V. Larsen; H. M. L. Andersen; L. J. Pedersen	2016	Can tail damage outbreaks in the pig be predicted by behavioural change?	Veterinary Journal	10.1016/j.tvjl.2015.12.001
112.	L. M. Rocha; A. Velarde; A. Dalmau; L. Saucier; L. Faucitano	2016	Can the monitoring of animal welfare parameters predict pork meat quality variation through the supply chain (from farm to slaughter)?	Journal of animal science	
113.	R. Carreras; L. Arroyo; E. Mainau; D. Valent; A. Bassols; A. Dalmau; L. Faucitano; X. Manteca; A. Velarde	2017	Can the way pigs are handled alter behavioural and physiological measures of affective state?	Behav Processes	10.1016/j.beproc.2017.06.005
114.	M. Rice; C. Baird; L. Stikeleather; W. E. M. Morrow; R. Meyer	2014	Carbon dioxide system for on-farm euthanasia of pigs in small groups	Journal of Swine Health and Production	
115.	U. Weiler; V. Stefanski; E. Von Borell	2016	Castration of pigs - conflicting aims and possible solutions from an animal welfare point of view	Zuchtungskunde	
116.	J. Garcia-Diez; A. C. Coelho	2014	Causes and factors related to pig carcass condemnation	Veterinarni Medicina	10.17221/7480-vetmed
117.	G. M. Corte; H. Hünigen; K. C. Richardson; S. M. Niehues; J. Plendl	2019	Cephalometric studies of the mandible, its masticatory muscles and vasculature of growing Göttingen Minipigs-A comparative anatomical study to refine experimental mandibular surgery	PLoS One	10.1371/journal.pone.0215875
118.	A. Horst; M. Gertz; J. Krieter	2019	Challenges and opportunities of using meat inspection data to improve pig health traits by breeding: A review	Livestock Science	10.1016/j.livsci.2019.02.001
119.	M. Becerril-Herrera; M. Alonso-Spilsbury; M. E. T. Ortega; I. Guerrero-Legarreta; R. Ramirez-Necoechea; P. Roldan-Santiago; M. Perez-Sato; E. Soni-Guillermo; D. Mota-Rojas	2010	Changes in blood constituents of swine transported for 8 or 16 h to an Abattoir	Meat Science	10.1016/j.meatsci.2010.07.021
120.	C. Bourguet; V. Deiss; M. Gobert; D. Durand; A. Boissy; E. M. C. Terlouw	2010	Characterising the emotional reactivity of cows to understand and predict their stress reactions to the slaughter procedure	Applied Animal Behaviour Science	10.1016/j.applanim.2010.03.008

121.	J. J. Zonderland; F. Schepers; M. B. M. Bracke; L. A. den Hartog; B. Kemp; H. A. M. Spoolder	2011	Characteristics of biter and victim piglets apparent before a tail-biting outbreak	Animal	10.1017/s1751731110002326
122.	Y. Xiong; A. Green; R. S. Gates	2015	Characteristics of Trailer Thermal Environment during Commercial Swine Transport Managed under U.S. Industry Guidelines	Animals (Basel)	10.3390/ani5020226
123.	S. P. Tseng; S. F. Wang; C. Y. Kuo; J. W. Huang; W. C. Hung; G. M. Ke; P. L. Lu	2015	Characterization of Fosfomycin Resistant Extended-Spectrum β-Lactamase-Producing Escherichia coli Isolates from Human and Pig in Taiwan	PLoS One	10.1371/journal.pone.0135864
124.	H. Aranishi; Y. Kunisawa; T. Komuro	2009	Characterization of interstitial cells of Cajal in the subserosal layer of the guinea-pig colon	Cell Tissue Res	10.1007/s00441-008-0730-5
125.	F. Tecles; D. Escribano; S. Martínez-Miró; F. Hernández; M. D. Contreras; J. J. Cerón	2016	Cholinesterase in porcine saliva: Analytical characterization and behavior after experimental stress	Res Vet Sci	10.1016/j.rvsc.2016.03.006
126.	Y. J. Cui; Y. Hao; J. L. Li; W. G. Bao; G. Li; Y. L. Gao; X. H. Gu	2016	Chronic Heat Stress Induces Immune Response, Oxidative Stress Response, and Apoptosis of Finishing Pig Liver: A Proteomic Approach	International Journal of Molecular Sciences	10.3390/ijms17050393
127.	M. Miele; J. Lever	2013	Civilizing the market for welfare friendly products in Europe? The techno-ethics of the Welfare Quality (R) assessment	Geoforum	10.1016/j.geoforum.2013.04.003
128.	M. S. Herskin; C. Holm; K. Thodberg	2020	Clinical and behavioural consequences of on-farm mixing of cull sows after weaning	Applied Animal Behaviour Science	10.1016/j.applanim.2020.105028
129.	M. Becerril-Herrera; M. Alonso-Spilsbury; C. Lemus-Flores; I. Guerrero-Legarreta; A. Olmos-Hernandez; R. Ramirez-Necoechea; D. Mota-Rojas	2009	CO2 stunning may compromise swine welfare compared with electrical stunning	Meat Science	10.1016/j.meatsci.2008.07.025
130.	T. N. Klauke; M. Piñeiro; S. Schulze-Geisthövel; S. Plattes; T. Selhorst; B. Petersen	2013	Coherence of animal health, welfare and carcass quality in pork production chains	Meat Sci	10.1016/j.meatsci.2013.03.022
131.	N. Cobanovic; U. Jamnikar-Ciglenecki; A. Kirbis; M. Krizman; M. Stukelj; I. Vicic; N. Karabasil	2020	Coherence of Clinical Symptoms at Antemortem Inspection and Pathological Lesions at Postmortem Inspection in Slaughter Pigs	Kafkas Universitesi Veteriner Fakultesi Dergisi	10.9775/kvfd.2020.23884
132.	M. A. Sutherland; P. J. Bryer; B. L. Davis; J. F. Smith; J. J. McGlone	2012	The combined effects of transport and food and water deprivation on the physiology of breeding age gilts	Livestock Science	10.1016/j.livsci.2011.11.005
133.	M. Salines; M. Andraud; N. Rose	2018	Combining network analysis with epidemiological data to inform risk-based surveillance: Application to hepatitis E virus (HEV) in pigs	Prev Vet Med	10.1016/j.prevetmed.2017.11.015
134.	L. Alban; J. V. Petersen; M. E. Busch	2015	A comparison between lesions found during meat inspection of finishing pigs raised under organic/free-range conditions and conventional, indoor conditions	Porcine Health Management	10.1186/2055-5660-1-4
135.	B. Żywicka; Z. Rybak; M. Janeczek; A. Czerski; J. Bujok; M. Szymonowicz; M. Dobrzyński; M. Korczyński; J. Świderski	2020	Comparison of A 1940 nm Thulium-Doped Fiber Laser and A 1470 nm Diode Laser for Cutting Efficacy and Hemostasis in A Pig Model of Spleen Surgery	Materials (Basel)	10.3390/ma13051167
136.	O. R. B. Velazco; S. C. Sanz; F. E. Barber; A. V. Garcia	2013	Comparison of extensive and intensive pig production systems in Uruguay in terms of ethologic, physiologic and meat quality parameters	Revista Brasileira De Zootecnia-Brazilian Journal of Animal Science	

137.	M. Martinez-Macipe; P. Rodríguez; M. Izquierdo; M. Gispert; X. Manteca; E. Mainau; F. I. Hernández; A. Claret; L. Guerrero; A. Dalmau	2016	Comparison of meat quality parameters in surgical castrated versus vaccinated against gonadotrophin-releasing factor male and female Iberian pigs reared in free-ranging conditions	Meat Sci	10.1016/j.meatsci.2015.09.002
138.	N. J. Kells; N. J. Beausoleil; C. B. Johnson; M. A. Sutherland; R. S. Morrison; W. Roe	2017	Comparison of neural histomorphology in tail tips from pigs docked using clippers or cautery iron	Animal	10.1017/s1751731116002500
139.	S. S. Nielsen; G. B. Nielsen; M. J. Denwood; J. Haugegaard; H. Houe	2015	Comparison of recording of pericarditis and lung disorders at routine meat inspection with findings at systematic health monitoring in Danish finisher pigs	Acta Vet Scand	10.1186/s13028-015-0109-z
140.	J. I. Eze; C. Correia-Gomes; J. Borobia-Belsué; A. W. Tucker; D. Sparrow; D. W. Strachan; G. J. Gunn	2015	Comparison of Respiratory Disease Prevalence among Voluntary Monitoring Systems for Pig Health and Welfare in the UK	PloS one	
141.	L. C. Hoffman; P. Fisher	2010	Comparison of the effects of different transport conditions and lairage times in a Mediterranean climate in South Africa on the meat quality of commercially crossbred Large white x Landrace pigs	Journal of the South African Veterinary Association-Tydskrif Van Die Suid-Afrikaanse Veterinere Vereniging	
142.	D. L. Teixeira; L. A. Boyle	2014	A comparison of the impact of behaviours performed by entire male and female pigs prior to slaughter on skin lesion scores of the carcass	Livestock Science	10.1016/j.livsci.2014.09.026
143.	S. Hoy; I. Jans-Wenstrup	2018	Conditions of occurrence and frequency of tail biting in fattening pigs	Tieraerztliche Umschau	
144.	P. Fanny; A. E. Sandra; M. Dominiek; K. Ilias	2018	Connecting Different Data Sources to Assess the Interconnections between Biosecurity, Health, Welfare, and Performance in Commercial Pig Farms in Great Britain	Frontiers in Veterinary Science, Vol	
145.	C. Terlouw; C. Bourguet; V. Deiss	2016	Consciousness, unconsciousness and death in the context of slaughter. Part II. Evaluation methods	Meat Sci	10.1016/j.meatsci.2016.03.010
146.	W. I. Sonntag; M. T. Kiehas; A. Spiller; A. Kaiser; L. M. Ludolph; K. G. Grunert; M. von Meyer-Hofer	2019	Consumer evaluation of intra-sustainable trade-offs in pig production - A mixed-method approach to analyze different consumer segments	Livestock Science	10.1016/j.livsci.2019.04.010
147.	G. T. Tonsor; C. Wolf; N. Olynk	2009	Consumer voting and demand behavior regarding swine gestation crates	Food Policy	10.1016/j.foodpol.2009.06.008
148.	G. Di Martino; K. Capello; A. Scollo; F. Gottardo; A. L. Stefani; F. Rampin; E. Schiavon; S. Marangon; L. Bonfanti	2013	Continuous straw provision reduces prevalence of oesophago-gastric ulcer in pigs slaughtered at 170 kg (heavy pigs)	Res Vet Sci	10.1016/j.rvsc.2013.08.012
149.	J. H. Choe; M. H. Choi; Y. C. Ryu; K. S. Lim; E. A. Lee; J. H. Kang; K. C. Hong; S. K. Lee; Y. T. Kim; S. S. Moon; K. W. Lee; M. S. Rhee; B. C. Kim	2015	Correlations among various blood parameters at exsanguination and their relationships to pork quality traits	Animal Production Science	10.1071/an13424
150.	C. Bergamin; A. Comin; M. Corazzin; M. Faustini; T. Peric; A. Scollo; F. Gottardo; M. Montillo; A. Prandi	2019	Cortisol, DHEA, and Sexual Steroid Concentrations in Fattening Pigs' Hair	Animals	10.3390/ani9060345
151.	S. Schukat; H. Heise	2019	Cost calculations of various animal welfare initiative scenarios in pig fattening using the example of a 1.920er fattening barn	Berichte Uber Landwirtschaft	

152.	J. A. Bouwsema; J. A. Lines	2019	Could low atmospheric pressure stunning (LAPS) be suitable for pig slaughter? A review of available information	Animal Welfare	10.7120/09627286.28.4.421
153.	A. C. D. Julia; M. Edgar García; D. Alessia; A. B. Laura	2018	Cross-Fostering Implications for Pig Mortality, Welfare and Performance	Frontiers in Veterinary Science, Vol	
154.	T. Steinmann; T. Blaha; D. Meemken	2018	Cross-location standardization of lung scoring during post-mortem inspection of fattening pigs under industrialized conditions at two different slaughterhouses	Berliner Und Munchener Tierarztliche Wochenschrift	10.2376/0005-9366-17015
155.	S. Viegas; L. A. Caetano; M. Korkalainen; T. Faria; C. Pacífico; E. Carolino; A. Quintal Gomes; C. Viegas	2017	Cytotoxic and Inflammatory Potential of Air Samples from Occupational Settings with Exposure to Organic Dust	Toxics	10.3390/toxics5010008
156.	S. Looden	2018	Das Leid auf den Ladeflächen		
157.	P. Rolzhauser; J. Saffaf; S. Wohlfahrt; L. Walter; A. Pahl; A. Hamedy; E. Lucke; K. Riehn	2019	The data collection of slaughtered pregnant pigs by competent veterinary authorities: findings from the SiGN-Project	Berliner Und Munchener Tierarztliche Wochenschrift	10.2376/0005-9366-18090
158.	H. Kongsted; L. Foldager; J. T. Sorensen	2020	Data from routine meat inspection is a poor indicator of the prevalence of tail lesions in undocked pigs	Porcine Health Management	10.1186/s40813-020-00149-z
159.	S. Shields; P. Shapiro; A. Rowan	2017	A Decade of Progress toward Ending the Intensive Confinement of Farm Animals in the United States	Animals	10.3390/ani7050040
160.	J. Nonneman Dan; T. Brown-Brandl; A. Jones Shuna; T. Wiedmann Ralph; A. Rohrer Gary	2012	A defect in dystrophin causes a novel porcine stress syndrome	BMC Genomics, Vol 13, Iss 1, p	
161.	I. Schwarzlose	2016	Definition, Erfassung und Optimierung von Parametern bei der Elektrobetäubung von Schlachtschweinen unter Tierschutz- und Fleischqualitätsaspekten (EPOS) ; Definition, recording and optimization of parameters for electrical stunning of slaughter pigs under aspects of animal welfare and meat quality (EPOS)		
162.	J. A. Calderón Díaz; A. Diana; L. A. Boyle; F. C. Leonard; M. McElroy; S. McGettrick; J. Moriarty; E. García Manzanilla	2017	Delaying pigs from the normal production flow is associated with health problems and poorer performance	Porcine Health Manag	10.1186/s40813-017-0061-6
163.	M. Eckhard	2016	Demands of pigs on the construction of tube feeders depending on gender	Landtechnik, Vol 71, Iss	
164.	A. Malmsten; N. Lundeheim; A.-M. Dalin	2020	Dental disorders in sows from Swedish commercial herds	Acta veterinaria Scandinavica	
165.	X. Wu; L. M. Gao; K. Zhou; X. Li; X. Lin; D. Wan; X. Xiong; G. Liu; Y. L. Yin	2018	Deposition and transport of trace mineral elements were affected by stocking density in fattening pigs	Journal of Trace Elements in Medicine and Biology	10.1016/j.jtemb.2018.04.007
166.	M. Hubert	2019	Der intelligente Stall<br>Mehr Tierwohl für Kuh und Schwein?		
167.	J. Efken; H. Bäurle; G. Haxsen; C. Niemann; C. Tamasy	2012	Der Markt für Fleisch und Fleischprodukte		
168.		2019	Der Schweinestall der Zukunft - auf Stroh. Die Haltung landwirtschaftlicher Nutztiere, insbesondere der Schweine, steht seit längerem im Fokus der Tierwohldebatte. Fast tägliche Meldungen in den verschiedenen Medien weisen auf häufig tierschutzwidrige Haltungsbedingungen hin	Rundschau für Fleischhygiene und Lebensmittelüberwachung	

169.	D. U. Thomson; G. H. Loneragan; J. N. Henningson; S. Ensley; B. Bawa	2015	Description of a novel fatigue syndrome of finished feedlot cattle following transportation	J Am Vet Med Assoc	10.2460/javma.247.1.66
170.	L. M. Mansley; A. I. Donaldson; M. V. Thrusfield; N. Honhold	2011	Destructive tension: mathematics versus experience--the progress and control of the 2001 foot and mouth disease epidemic in Great Britain	Rev Sci Tech	10.20506/rst.30.2.2054
171.	S. R. Clegg; L. E. Sullivan; J. Bell; R. W. Blowey; S. D. Carter; N. J. Evans	2016	Detection and isolation of digital dermatitis treponemes from skin and tail lesions in pigs	Research in veterinary science	
172.	N. Jerez-Timaure; M. T. Sulbaran; L. A. de Moreno; A. Rodas-Gonzalez; J. Trompiz; J. Ortega	2013	Detection of quality failures in pork carcass and meat using farm and plant audit	Revista Mexicana De Ciencias Pecuarias	
173.	R. Martinez-Rodriguez; P. Roldan-Santiago; S. Flores-Peinado; J. A. Ramirez-Telles; P. Mora-Medina; M. E. Trujillo-Ortega; M. Gonzalez-Lozano; M. Becerril-Herrera; M. Sanchez-Hernandez; D. Mota-Rojas	2011	Deterioration of Pork Quality Due to the Effects of Acute Ante Mortem Stress: An Overview	Asian Journal of Animal and Veterinary Advances	10.3923/ajava.2011.1170.1184
174.	S. Ngarava; A. Mushunje	2018	Determinants of pricing objectives and price flexibility policies of pork-based agro-businesses in Mashonaland Central province, Zimbabwe	South African Journal of Economic and Management Sciences	10.4102/sajems.v21i1.2029
175.	E. Quendler; P. Pötz; W. Hagmüller; R. Kogler; J. Boxberger	2013	Determination of the working time requirement for suckling sows in the pen of Wels	Journal of Agricultural Engineering, Vol 44, Iss 2s (2013)	
176.	P. Trevisi; M. Bertocchi; D. Luise; M. Mazzoni; L. N. Costa; P. Bosi	2019	Developing and testing a new feed block for the gut health and welfare of the weaning pig	Italian Journal of Animal Science	10.1080/1828051x.2019.1621215
177.	T. Grandin	2012	Developing measures to audit welfare of cattle and pigs at slaughter	Animal Welfare	10.7120/09627286.21.3.351
178.	N. B. Goecke; C. K. Hjulsager; J. S. Krog; K. Skovgaard; L. E. Larsen	2019	Development of a high-throughput real-time PCR system for detection of enzootic pathogens in pigs	Journal of veterinary diagnostic investigation : official publication of the American Association of Veterinary Laboratory Diagnosticians, Inc	
179.	P. Brandt; T. Rousing; M. S. Herskin; E. V. Olsen; M. D. Aaslyng	2017	Development of an index for the assessment of welfare of finishing pigs from farm to slaughter based on expert opinion	Livestock Science	10.1016/j.livsci.2017.02.008
180.	M. Machtolf; M. Moje; K. Troeger; M. Bülte	2013	Die Betäubung von Schlachtschweinen mit Helium ; Stunning slaughter pigs with helium		
181.	M. Machtolf; M. Moje; K. Troeger; M. Bülte	2013	Die Betäubung von Schlachtschweinen mit Helium und Kohlendioxid im Vergleich. Auswirkungen auf das Tierwohl sowie die Schlachtkörper- und Fleischqualität<br>Stunning slaughter pigs with helium and carbon dioxide - a comparison. Impact on animal welfare and on carcass and meat quality		
182.	M. Machtolf	2012	Die Betäubung von Schlachtschweinen mit Kohlendioxid und Stickstoff in Mischungen - Auswirkungen auf das Tierwohl und die Fleischqualität	Mitteilungsblatt Fleischforschung Kulmbach	

183.	M. Machtolf; K. Troeger	2013	Die Gasbetäubung von Schlachtschweinen Alternativen zum Einsatz von Kohlendioxid		
184.	H. Boytchev	2016	Die Seucheninsel		
185.	C. Lauer	2018	Die Wurst soll schmecken - und kein Ferkel leiden		
186.	M. Federica; R. Raffaella; R. Sabrina; C. Sara; C. Carlo	2016	Dietary supplementation with algae and polyphenols in lactating sows effects on sows and piglets' performance	International Journal of Health, Animal Science and Food Safety, Vol 3, Iss 1s (2016)	
187.	H. Hartmann; C. Siegling-Vlitakis; K. Wolf; G. Rindermann; R. Fries	2009	Different CO2-stunning procedures and post mortem obtained lung lesions in response to the corneal reflex and parameters in blood of slaughtered pigs	Berliner Und Munchener Tierarztliche Wochenschrift	10.2376/0005-9366-122-333
188.	S. Martín-Peláez; B. Peralta; E. Creus; A. Dalmau; A. Velarde; J. F. Pérez; E. Mateu; S. M. Martín-Orúe	2009	Different feed withdrawal times before slaughter influence caecal fermentation and faecal Salmonella shedding in pigs	Vet J	10.1016/j.tvjl.2008.08.002
189.	D. Mörlein; A. Grave; A. R. Sharifi; M. Bücking; M. Wicke	2012	Different scalding techniques do not affect boar taint	Meat Sci	10.1016/j.meatsci.2012.02.028
190.	M. Greger; S. Parente; M. C. Appleby; J. L. Lanier	2010	DISEASE AND TRANSPORT: A COSTLY TICKET AROUND THE WORLD	Handbook of Disease Outbreaks: Prevention, Detection and Control	
191.	N. Dadios; J. Hardstaff; S. Alonso; K. Staerk; A. Lindberg	2014	Disease monitoring in farmed game: the role of abattoir meat inspection	Trends in Game Meat Hygiene: From Forest to Fork	10.3920/978-90-8686-238-2_4
192.	D. Conficoni; M. Santagiuliana; M. Marchesan; F. Franceschini; P. Catellani; M. Ferioli; V. Giaccone	2019	Distribution of Listeria spp. on Carcasses of Regularly Slaughtered Swine for Italian Dry Cured Ham	J Food Prot	10.4315/0362-028x.Jfp-18-599
193.	M. Giovanna; T. Lucio; S. Luca; C. Massimo	2010	Do mood disorders play a role in pig welfare?	Italian Journal of Animal Science, Vol 8, Iss 4, Pp 691-	
194.	H. Arndt; N. Volkmann; B. Spindler; J. Hartung; N. Kemper	2019	Do Pigs Have Adequate Space in Animal Transportation Vehicles?-Planimetric Measurement of the Floor Area Covered by Finishing Pigs in Various Body Positions	Frontiers in Veterinary Science	10.3389/fvets.2018.00330
195.	C. Ludovic Toma; N. S. Evangelia; S. c. Endre; A. i. Stelian; E. Silvia; B. Simona; T. Iulian; G. Dinu	2011	Do Romanian Farmers and Consumers Have Different Views on Farm Animal Welfare Issues?	Scientific Papers Animal Science and Biotechnologies, Vol 44, Iss 2, Pp 285-	
196.	S.-L. A. Schild; T. Rousing; H. E. Jensen; K. Barington; M. S. Herskin	2015	Do umbilical outpouchings affect the behaviour or clinical condition of pigs during 6 h housing in a pre-transport pick-up facility?	Research in veterinary science	
197.	S. Harley; L. A. Boyle; N. E. O'Connell; S. J. More; D. L. Teixeira; A. Hanlon	2014	Docking the value of pigmeat? Prevalence and financial implications of welfare lesions in Irish slaughter pigs	Animal Welfare	10.7120/09627286.23.3.275
198.	Y. Y. W. Lau; J. R. Pluske; P. A. Fleming	2015	Does the environmental background (intensive v. outdoor systems) influence the behaviour of piglets at weaning?	Animal	10.1017/s1751731115000531
199.	S. L. A. Schild; P. Brandt; T. Rousing; M. S. Herskin	2015	Does the presence of umbilical outpouchings affect the behaviour of pigs during the day of slaughter?	Livestock Science	10.1016/j.livsci.2015.03.023

200.	K. H. Jensen; L. Jørgensen; S. Haugegaard; M. S. Herskin; M. B. Jensen; L. J. Pedersen; N. Canibe	2017	The dose-response relationship between the amount of straw provided on the floor and gastric ulceration of pars oesophagea in growing pigs	Res Vet Sci	10.1016/j.rvsc.2017.01.005
201.	M. Wedin; E. M. Baxter; M. Jack; A. Futro; R. B. D'Eath	2018	Early indicators of tail biting outbreaks in pigs	Applied Animal Behaviour Science	10.1016/j.applanim.2018.08.008
202.	J. A. C. Diaz; L. A. Boyle; A. Diana; F. C. Leonard; J. P. Moriarty; M. C. McElroy; S. McGettrick; D. Kelliher; E. G. Manzanilla	2017	Early life indicators predict mortality, illness, reduced welfare and carcass characteristics in finisher pigs	Preventive Veterinary Medicine	10.1016/j.prevetmed.2017.07.018
203.	K. Andersson; C. Brunius; G. Zamaratskaia; K. Lundström	2012	Early vaccination with Improvac®: effects on performance and behaviour of male pigs	Animal	10.1017/s1751731111001200
204.	M. Andraud; M. Casas; N. Pavio; N. Rose	2014	Early-life hepatitis e infection in pigs: the importance of maternally-derived antibodies	PLoS One	10.1371/journal.pone.0105527
205.	C. Filipe Antonio Dalla; C. Osmar Antonio Dalla; C. Izabela Cruvinel Di; G. Neville George; C. Melissa Selaysim Di; L. Guilherme Brunno de Medeiros; T. Fernando de Castro	2019	Ease of Handling and Physiological Parameters of Stress, Carcasses, and Pork Quality of Pigs Handled in Different Group Sizes	Animals, Vol 9, Iss 10, p	
206.	X. Guo; G. D. Claassen; A. G. Oude Lansink; W. Loeffen; H. W. Saatkamp	2016	Economic Analysis of Classical Swine Fever Surveillance in the Netherlands	Transbound Emerg Dis	10.1111/tbed.12274
207.	S. Katharina; M. Sören; T. Ludwig	2018	Economic evaluation of selected animal welfare measures in pig farming from a single farm perspective	Landtechnik, Vol 73, Iss	
208.	T. J. Gibson; E. L. Jackson	2017	The economics of animal welfare	Revue scientifique et technique (International Office of Epizootics)	
209.	J. Heikkila	2011	Economics of biosecurity across levels of decision-making: a review	Agronomy for Sustainable Development	10.1051/agro/2010003
210.	C. Osmar Antonio Dalla; L. Jorge Vitor; C. Arlei; K. Jalusa Deon; C. Mateus José Rodrigues Paranhos da; F. Luigi; P. José Vicente; R. Darlan Dalla	2009	Efeito do manejo pré-abate sobre alguns parâmetros fisiológicos em fêmeas suínas pesadas Effect of pre-slaughter management on physiological parameters of heavy-weight female pigs	Ciência Rural, Vol 39, Iss 3, Pp 852-	
211.	J. Willamil; E. Creus; J. F. Pérez; E. Mateu; S. M. Martín-Orúe	2011	Effect of a microencapsulated feed additive of lactic and formic acid on the prevalence of Salmonella in pigs arriving at the abattoir	Arch Anim Nutr	10.1080/1745039x.2011.623047
212.	D. Lemos Teixeira; L. Lykke; L. Boyle	2020	The Effect of a Novel Transport System on the Welfare and Meat Quality of Slaughter Pigs	J Appl Anim Welf Sci	10.1080/10888705.2020.1790370
213.	B. Karaconji; B. Lloyd; N. Campbell; D. Meaney; T. Ahern	2015	Effect of an anti-gonadotropin-releasing factor vaccine on sexual and aggressive behaviour in male pigs during the finishing period under Australian field conditions	Aust Vet J	10.1111/avj.12307
214.	A. Rubio-Gonzalez; Y. Potes; D. Illan-Rodriguez; I. Vega-Naredo; V. Sierra; B. Caballero; E. Fabrega; A. Velarde; A. Dalmau; M. Olivan; A. Coto-Montes	2015	Effect of animal mixing as a stressor on biomarkers of autophagy and oxidative stress during pig muscle maturation	Animal	10.1017/s1751731115000518
215.	O. O. Asala; J. O. Ayo; P. I. Rekwot; N. S. Minka; D. O. Omoniwa; A. Y. Adenkola	2011	Effect of ascorbic acid administration on erythrocyte osmotic fragility of pigs transported by road during the hot-dry season	Vet Res Commun	10.1007/s11259-011-9469-7

216.	M. P. Serrano; D. G. Valencia; A. Fuentetaja; R. Lazaro; G. G. Mateos	2009	Effect of castration on productive performance, carcass characteristics and meat quality of Iberian pig females reared under intensive management systems	Livestock Science	10.1016/j.livsci.2008.11.001
217.	Á. Végh; Z. Abonyi-Tóth; P. Rafai	2017	Effect of current intensity and duration on the effectiveness of head-only electrical stunning in pigs under commercial conditions	Acta Vet Hung	10.1556/004.2017.002
218.	V. A. de Lima; M. C. Ceballos; N. G. Gregory; M. Da Costa	2019	Effect of different catching practices during manual upright handling on broiler welfare and behavior	Poult Sci	10.3382/ps/pez284
219.	A. Garcia; L. Gil; C. Malo; F. Martinez; C. Kershaw-Young; I. de Blas	2014	Effect of different disaccharides on the integrity and fertilising ability of freeze-dried boar spermatozoa: a preliminary study	Cryo Letters	
220.	P. B. Gade; L. Christensen	2011	Effect of different stocking densities during transport on welfare and meat quality in Danish slaughter pigs	Meat science	
221.	K. Thodberg; M. S. Herskin; T. Jensen; K. H. Jensen	2018	The effect of docking length on the risk of tail biting, tail-directed behaviour, aggression and activity level of growing pigs kept under commercial conditions	Animal : an international journal of animal bioscience	10.1017/s1751731118000563
222.	A. Mason; E. Tolo; L. Hektoen; H. A. Haga	2018	The effect of electrical head-to-chest stunning on the EEG in sheep	Animal Welfare	10.7120/09627286.27.4.343
223.	N. Casal; M. Font-i-Furnols; M. Gispert; X. Manteca; E. Fabrega	2018	Effect of Environmental Enrichment and Herbal Compounds-Supplemented Diet on Pig Carcass, Meat Quality Traits, and Consumers' Acceptability and Preference	Animals	10.3390/ani8070118
224.	A. Yanez-Pizana; D. Mota-Rojas; M. Castillo-Rivera; R. Ramirez-Necoechea; I. Guerrero-Legarreta; P. Mora-Medina; M. Gonzalez-Lozano	2019	Effect of environmental enrichment on weaned piglets: physiological responses	Veterinarni Medicina	10.17221/104/2018-vetmed
225.	N. Panella-Riera; M. Gispert; M. Gil; J. Soler; J. Tibau; M. A. Oliver; A. Velarde; E. Fabrega	2012	Effect of feed deprivation and lairage time on carcass and meat quality traits on pigs under minimal stressful conditions	Livestock Science	10.1016/j.livsci.2012.02.017
226.	N. Quiniou; M. Monziols; F. Colin; T. Goues; V. Courboulay	2012	Effect of feed restriction on the performance and behaviour of pigs immunologically castrated with Improvac®	Animal	10.1017/s1751731112000444
227.	V. E. Beattie; M. S. Burrows; B. W. Moss; R. N. Weatherup	2011	The effect of food deprivation prior to slaughter on performance, behaviour and meat quality	Meat science	
228.	H. Phoebe; B. Laura; Y. Bridget; O. D. Keelin	2019	The Effect of Group Composition and Mineral Supplementation during Rearing on Measures of Cartilage Condition and Bone Mineral Density in Replacement Gilts	Animals, Vol 9, Iss 9, p	
229.	J. A. Hagenmaier; C. D. Reinhardt; S. J. Bartle; J. N. Henningson; M. J. Ritter; M. S. Calvo-Lorenzo; G. J. Vogel; C. A. Guthrie; M. G. Siemens; D. U. Thomson	2017	Effect of handling intensity at the time of transport for slaughter on physiological response and carcass characteristics in beef cattle fed ractopamine hydrochloride	Journal of Animal Science	10.2527/jas.2016.0821
230.	L. Arroyo; R. Carreras; D. Valent; R. Peña; E. Mainau; A. Velarde; J. Sabrià; A. Bassols	2016	Effect of handling on neurotransmitter profile in pig brain according to fear related behaviour	Physiol Behav	10.1016/j.physbeh.2016.10.005
231.	F. R. Dunshea; J. R. D. Allison; M. Bertram; D. D. Boler; L. Brossard; R. Campbell; J. P. Crane; D. P. Hennessy; L. Huber; C. de Lange; N. Ferguson; P. Matzat; F. McKeith; P. J. U. Moraes; B. P. Mullan; J. Noblet; N. Quiniou; M. Tokach	2013	The effect of immunization against GnRF on nutrient requirements of male pigs: a review	Animal : an international journal of animal bioscience	

232.	K. Rypula; M. Porowski; J. Kaba; M. Gorczykowski; A. Deniz	2012	Effect of Isosporiasis Prevention with Toltrazuril on Long-Term Pig Performance	Scientific World Journal	10.1100/2012/486324
233.	M. F. o. V. M. B. Balti; M. F. o. V. M. B. Dokmanovi; N. F. o. V. M. B. Karabasil; S. F. o. A. B. Z. Hristov; R. F. o. V. M. B. Markovi; N. F. o. V. M. B. Glamo_lija; M. F. o. V. M. B. Todorovi	2012	Effect of lairage conditions and time on pork quality (a review)	Savremena poljoprivreda (Serbia)	
234.	M. Dokmanovic; J. Ivanovic; J. Janjic; M. Boskovic; M. Laudanovic; S. Pantic; M. Z. Baltic	2017	Effect of lairage time, behaviour and gender on stress and meat quality parameters in pigs	Anim Sci J	10.1111/asj.12649
235.	N. Panella-Riera; A. Velarde; A. Dalmau; E. Fabrega; M. F. I. Furnols; M. Gispert; J. Soler; J. Tibau; M. A. Oliver; M. Gil	2009	Effect of magnesium sulphate and L-tryptophan and genotype on the feed intake, behaviour and meat quality of pigs	Livestock Science	10.1016/j.livsci.2009.02.010
236.	S. Nienke van; T. Dayane Lemos; H. Alison; B. Laura Ann	2015	The effect of mixing entire male pigs prior to transport to slaughter on behaviour, welfare and carcass lesions	PLoS ONE, Vol 10, Iss 4, p e	
237.	E. Fabrega; X. Puigvert; J. Soler; J. Tibau; A. Dalmau	2013	Effect of on farm mixing and slaughter strategy on behaviour, welfare and productivity in Duroc finished entire male pigs	Applied Animal Behaviour Science	10.1016/j.applanim.2012.11.006
238.	R. E. Meyer; J. T. Whitley; W. E. M. Morrow; L. F. Stikeleather; C. L. Baird; J. M. Rice; B. V. Halbert; D. K. Styles; C. S. Whisnant	2013	Effect of physical and inhaled euthanasia methods on hormonal measures of stress in pigs	Journal of Swine Health and Production	
239.	J. Chai; Q. Xiong; C. X. Zhang; W. Miao; F. E. Li; R. Zheng; J. Peng; S. W. Jiang	2010	Effect of pre-slaughter transport plant on blood constituents and meat quality in halothane genotype of NN Large White x Landrace pigs	Livestock Science	10.1016/j.livsci.2009.09.014
240.	G. Arlene; P. Glenna; P. Guilherme; M. Matthew; G. Kimberly; B. Brittany; S. Mhairi; M. John	2015	Effect of Provision of Feed and Water during Transport on the Welfare of Weaned Pigs	Animals, Vol 5, Iss 2, Pp 407-	
241.	S. Goumon; L. Faucitano; R. Bergeron; T. Crowe; M. L. Connor; H. W. Gonyou	2013	Effect of ramp configuration on easiness of handling, heart rate, and behavior of near-market weight pigs at unloading	J Anim Sci	10.2527/jas.2012-6083
242.	C. Terlouw; A. Berne; T. Astruc	2009	Effect of rearing and slaughter conditions on behaviour, physiology and meat quality of Large White and Duroc-sired pigs	Livestock Science	10.1016/j.livsci.2008.08.016
243.	M. A. Gerritzen; V. A. Hindle; K. Steinkamp; H. G. M. Reimert; J. T. N. van der Werf; M. Marahrens	2013	The effect of reduced loading density on pig welfare during long distance transport	Animal : an international journal of animal bioscience	
244.	B. Fredriksen; C. Hexeberg	2009	The effect of removing animals for slaughter on the behaviour of the remaining male and female pigs in the pen	Res Vet Sci	10.1016/j.rvsc.2008.06.005
245.	S. Goumon; J. A. Brown; L. Faucitano; R. Bergeron; T. Crowe; M. L. Connor; H. W. Gonyou	2013	Effect of rest duration on recovery from repeated exercise in near-market-weight pigs	Journal of Animal Science	10.2527/jas.2012-6184
246.	D. Newman; J. Young; C. Carr; M. Ryan; E. Berg	2014	Effect of Season, Transport Length, Deck Location, and Lairage Length on Pork Quality and Blood Cortisol Concentrations of Market Hogs	Animals (Basel)	10.3390/ani4040627
247.	D. Knecht; K. Duzinski	2016	The effect of sex, carcass mass, back fat thickness and lean meat content on pork ham and loin characteristics	Archives Animal Breeding	10.5194/aab-59-51-2016
248.	S. Conte; P. G. Lawlor; N. O'Connell; L. A. Boyle	2012	Effect of split marketing on the welfare, performance, and carcass traits of finishing pigs	Journal of animal science	

249.	H. J. Guise; H. L. Riches; E. J. Hunter; T. A. Jones; P. D. Warriss; P. J. Kettlewell	2011	The effect of stocking density in transit on the carcass quality and welfare of slaughter pigs: 1. Carcass measurements	Meat science	
250.	P. D. Warriss; S. N. Brown; T. G. Knowles; J. E. Edwards; P. J. Kettlewell; H. J. Guise	2011	The effect of stocking density in transit on the carcass quality and welfare of slaughter pigs: 2. Results from the analysis of blood and meat samples	Meat science	
251.	D. L. Teixeira; G. C. Miranda-de la Lama; M. Villarroel; S. Garcia-Belenguer; C. Sanudo; G. A. Maria	2012	Effect of straw on lamb welfare, production performance and meat quality during the finishing phase of fattening	Meat Science	10.1016/j.meatsci.2012.07.009
252.	V. Resendiz-Cruz; J. E. Ramirez-Bribiesca; D. Mota-Rojas; I. Guerrero-Legarreta; R. G. Cruz-Monterrosa; D. Hernandez-Sanchez	2018	The effect of stress on haematologic response and physicochemical parameters of muscle meat in rabbits	Journal of Animal Physiology and Animal Nutrition	10.1111/jpn.12759
253.	M. Tretola; F. Maghin; P. Silacci; S. Ampuero; G. Bee	2019	Effect of Supplementing Hydrolysable Tannins to a Grower-Finisher Diet Containing Divergent PUFA Levels on Growth Performance, Boar Taint Levels in Back Fat and Intestinal Microbiota of Entire Males	Animals	10.3390/ani9121063
254.	G. Di Martino; A. Scollo; F. Gottardo; A. L. Stefani; E. Schiavon; K. Capello; S. Marangon; L. Bonfanti	2015	The effect of tail docking on the welfare of pigs housed under challenging conditions	Livestock Science	10.1016/j.livsci.2014.12.012
255.	S. Conte; L. A. Boyle; N. E. O'Connell; P. B. Lynch; P. G. Lawlor	2011	Effect of target slaughter weight on production efficiency, carcass traits and behaviour of restrictively-fed gilts and intact male finisher pigs	Livestock Science	10.1016/j.livsci.2010.08.018
256.	X. Averos; A. Herranz; R. Sanchez; L. F. Gosalvez	2009	Effect of the duration of commercial journeys between rearing farms and growing-finishing farms on the physiological stress response of weaned piglets	Livestock Science	10.1016/j.livsci.2008.09.019
257.	A. Van den Broeke; M. Aluwé; S. Janssens; J. Wauters; L. Vanhaecke; N. Buys; S. Millet; F. A. M. Tuyttens	2015	The effect of the MC4R gene on boar taint compounds, sexual maturity and behaviour in growing-finishing boars and gilts	Animal : an international journal of animal bioscience	
258.	M. H. Anil; P. E. Whittington; J. L. McKinstry	2011	The effect of the sticking method on the welfare of slaughter pigs	Meat science	
259.	M. A. Sutherland; N. Krebs; J. S. Smith; J. W. Dailey; J. A. Carroll; J. J. McGlone	2009	The effect of three space allowances on the physiology and behavior of weaned pigs during transportation	Livestock Science	10.1016/j.livsci.2009.06.021
260.	R. V. Knox; J. Shen; L. L. Greiner; J. F. Connor	2016	Effect of timing of relocation of replacement gilts from group pens to individual stalls before breeding on fertility and well-being	Journal of animal science	
261.	T. L. Pereira; A. Corassa; C. M. Komiyama; A. P. S. Ton; C. V. de Araujo; J. L. Stuani; R. M. Honorio	2017	THE EFFECT OF TRANSPORT DENSITY AND GENDER ON SKIN TEMPERATURE AND CARCASS AND MEAT QUALITY IN PIGS	Bioscience Journal	
262.	T. L. Pereira; A. Corassa; C. M. Komiyama; C. V. Araujo; A. Kataoka	2015	The effect of transport density and gender on stress indicators and carcass and meat quality in pigs	Spanish Journal of Agricultural Research	10.5424/sjar/2015133-6638
263.	M. P. Pérez; J. Palacio; M. P. Santolaria; M. C. Aceña; G. Chacón; M. Gascón; J. H. Calvo; P. Zaragoza; J. A. Beltran; S. García-Belenguer	2011	Effect of transport time on welfare and meat quality in pigs	Meat science	
264.	S. T. Machado; I. D. Naas; M. M. Neto; O. Vendrametto; J. G. M. Dos Reis	2016	EFFECT OF TRANSPORTATION DISTANCE ON WEIGHT LOSSES IN PIGS FROM DEHYDRATION	Engenharia Agricola	10.1590/1809-4430-Eng.Agric.v36n6p1229-1238/2016

265.	V. V. de Perre; L. Permentier; S. Bie; G. Verbeke; R. Geers	2010	Effect of unloading, lairage, pig handling, stunning and season on pH of pork	Meat Science	10.1016/j.meatsci.2010.07.019
266.	E. Fàbrega	2010	Effect of vaccination against gonadotrophin-releasing hormone, using Improvac®, on growth performance, body composition, behaviour and acute phase proteins	Livestock science. 2010 Aug., v. 132, no. 1-3	
267.	D. C. Canaday; J. L. Salak-Johnson; A. M. Visconti; X. Wang; K. Bhalerao; R. V. Knox	2013	Effect of variability in lighting and temperature environments for mature gilts housed in gestation crates on measures of reproduction and animal well-being	J Anim Sci	10.2527/jas.2012-5733
268.	M. Karwowska; J. Mikolajczak; Z. J. Dolatowski; S. Borowski	2015	THE EFFECT OF VARYING DISTANCES FROM THE WIND TURBINE ON MEAT QUALITY OF GROWING-FINISHING PIGS	Annals of Animal Science	10.1515/aoas-2015-0051
269.	P. J. Bryer; M. A. Sutherland; B. L. Davis; J. F. Smith; J. J. McGlone	2011	The effect transport and space allowance on the physiology of breeding age gilts	Livestock Science	10.1016/j.livsci.2010.09.026
270.	M. T. Young; A. L. French; J. W. Clymer	2011	An effective, economical method of reducing environmental noise in the vivarium	J Am Assoc Lab Anim Sci	
271.	T. M. Casey-Trott; S. T. Millman; P. V. Turner; S. G. Nykamp; P. C. Lawlis; T. M. Widowski	2014	Effectiveness of a nonpenetrating captive bolt for euthanasia of 3 kg to 9 kg pigs	J Anim Sci	10.2527/jas.2014-7980
272.	T. M. Casey-Trott; S. T. Millman; P. V. Turner; S. G. Nykamp; T. M. Widowski	2013	Effectiveness of a nonpenetrating captive bolt for euthanasia of piglets less than 3 d of age	J Anim Sci	10.2527/jas.2013-6320
273.	R. Svenja; C. Lucie; P. Merel; B. Annette; S. Marie; B. Catherine; E. Ulf; B. Elisabeth grosse; S. Katharina; D. Jeroen; M. C. on the behalf of the	2020	Effectiveness of alternative measures to reduce antimicrobial usage in pig production in four European countries	Porcine Health Management, Vol 6, Iss 1, Pp 1-	
274.	S. E. O. Oliveira; N. G. Gregory; F. A. Dalla Costa; T. J. Gibson; O. A. Dalla Costa; M. J. R. Paranhos da Costa	2018	Effectiveness of pneumatically powered penetrating and non-penetrating captive bolts in stunning cattle	Meat Sci	10.1016/j.meatsci.2018.02.010
275.	R. Poletto; M. H. Rostagno; B. T. Richert; J. N. Marchant-Forde	2009	Effects of a step-up" ractopamine feeding program, sex, and social rank on growth performance, hoof lesions, and Enterobacteriaceae shedding in finishing pigs"	Journal of animal science	
276.	F. A. D. Costa; N. Devillers; M. da Costa; L. Faucitano	2016	Effects of applying preslaughter feed withdrawal at the abattoir on behaviour, blood parameters and meat quality in pigs	Meat Science	10.1016/j.meatsci.2016.03.033
277.	E. Voslarova; P. Chloupek; J. Chloupek; I. Bedanova; V. Pistekova; V. Vecerek	2011	The effects of chronic intermittent noise exposure on broiler chicken performance	Animal Science Journal	10.1111/j.1740-0929.2011.00877.x
278.	F. J. van der Staay; T. Schuurman; M. Hulst; M. Smits; J. Prickaerts; G. Kenis; S. M. Korte	2010	Effects of chronic stress: A comparison between tethered and loose sows	Physiology & Behavior	10.1016/j.physbeh.2010.02.020
279.	C. Munsterhjelm; M. Heinonen; A. Valros	2015	Effects of clinical lameness and tail biting lesions on voluntary feed intake in growing pigs	Livestock Science	10.1016/j.livsci.2015.09.003
280.	J. Kamenik; F. Conte; E. Voslarova; J. Blahova; P. Marsalek; I. Bedanova; V. Vecerek	2018	Effects of CO2 and electrical stunning methods on selected plasma indices in pigs	Berliner Und Munchener Tierarztliche Wochenschrift	10.2376/0005-9366-16052
281.	M. G. S. McKendree; C. C. Croney; N. J. O. Widmar	2014	Effects of demographic factors and information sources on United States consumer perceptions of animal welfare	Journal of animal science	
282.	T. Zhang; Y. F. Zhou; Y. Zou; X. M. Hu; L. F. Zheng; H. K. Wei; I. Giannenas; L. Z. Jin; J. Peng; S. W. Jiang	2015	Effects of dietary oregano essential oil supplementation on the stress response, antioxidative capacity, and HSPs mRNA expression of transported pigs	Livestock Science	10.1016/j.livsci.2015.05.037

283.	J. A. Correa; S. Torrey; N. Devillers; J. P. Laforest; H. W. Gonyou; L. Faucitano	2010	Effects of different moving devices at loading on stress response and meat quality in pigs	J Anim Sci	10.2527/jas.2010-2833
284.	M. S. Cockram; J. Y. Spence	2012	The effects of driving events on the stability and resting behaviour of cattle, young calves and pigs	Animal Welfare	10.7120/09627286.21.3.403
285.	J. Li; Q. Han; R. Liu; P. Wen; W. Ji; L. Pan; C. Wang; P. Zhao; H. Liu; J. Bao	2020	Effects of environment and breed on growth performance and meat quality of fattening pigs	Animal Welfare	10.7120/09627286.29.2.177
286.	E. Fàbrega; M. Marcet-Rius; R. Vidal; D. Escribano; J. J. Cerón; X. Manteca; A. Velarde	2019	The Effects of Environmental Enrichment on the Physiology, Behaviour, Productivity and Meat Quality of Pigs Raised in a Hot Climate	Animals (Basel)	10.3390/ani9050235
287.	N. Krebs	2009	Effects of exposing pigs to moving and odors in a simulated slaughter chute	Applied animal behaviour science. 2009 Jan. 31, v. 116, no. 2-4	
288.	L. Faucitano; P. Chevillon; M. Ellis	2010	Effects of feed withdrawal prior to slaughter and nutrition on stomach weight, and carcass and meat quality in pigs	Livestock Science	10.1016/j.livsci.2009.10.002
289.	J. D. Acevedo-Giraldo; J. A. Sánchez; M. H. Romero	2019	Effects of feed withdrawal times prior to slaughter on some animal welfare indicators and meat quality traits in commercial pigs	Meat science	
290.	C. M. Pilcher; M. Ellis; A. Rojo-Gómez; S. E. Curtis; B. F. Wolter; C. M. Peterson; B. A. Peterson; M. J. Ritter; J. Brinkmann	2011	Effects of floor space during transport and journey time on indicators of stress and transport losses of market-weight pigs	J Anim Sci	10.2527/jas.2010-3143
291.	K. R. Stackhouse-Lawson; C. B. Tucker; M. S. Calvo-Lorenzo; F. M. Mitloehner	2015	Effects of growth-promoting technology on feedlot cattle behavior in the 21 days before slaughter	Applied Animal Behaviour Science	10.1016/j.applanim.2014.11.001
292.	E. Fàbrega; X. Manteca; J. Font; M. Gispert; D. Carrión; A. Velarde; J. L. Ruiz-de-la-Torre; A. Diestre	2011	Effects of halothane gene and pre-slaughter treatment on meat quality and welfare from two pig crosses	Meat science	
293.	E. M. van Grevenhof; S. Ott; W. Hazeleger; P. R. van Weeren; P. Bijma; B. Kemp	2011	The effects of housing system and feeding level on the joint-specific prevalence of osteochondrosis in fattening pigs	Livestock Science	10.1016/j.livsci.2010.06.010
294.	B. Driessen; S. V. Beirendonck; J. Buyse	2020	Effects of Housing, Short Distance Transport and Lairage on Meat Quality of Finisher Pigs	Animals (Basel)	10.3390/ani10050788
295.	E. Nannoni; G. Martelli; G. Rubini; L. Sardi	2019	Effects of increased space allowance on animal welfare, meat and ham quality of heavy pigs slaughtered at 160Kg	PLoS One	10.1371/journal.pone.0212417
296.	H. W. Liu; R. Z. Zhong; D. W. Zhou; H. X. Sun; C. S. Zhao	2012	Effects of lairage time after road transport on some blood indicators of welfare and meat quality traits in sheep	Journal of Animal Physiology and Animal Nutrition	10.1111/j.1439-0396.2011.01230.x
297.	M. Dokmanovic; A. Velarde; V. Tomovic; N. Glamoclija; R. Markovic; J. Janjic; M. Z. Baltic	2014	The effects of lairage time and handling procedure prior to slaughter on stress and meat quality parameters in pigs	Meat Science	10.1016/j.meatsci.2014.06.003
298.	S. Zhen; Y. Liu; X. Li; K. Ge; H. Chen; C. Li; F. Ren	2013	Effects of lairage time on welfare indicators, energy metabolism and meat quality of pigs in Beijing	Meat Sci	10.1016/j.meatsci.2012.09.008
299.	G. Mazzone; G. Vignola; M. Giammarco; A. C. Manetta; L. Lambertini	2010	Effects of loading methods on rabbit welfare and meat quality	Meat Science	10.1016/j.meatsci.2009.11.019
300.	D. Mota-Rojas; M. Becerril-Herrera; P. Roldan-Santiago; M. Alonso-Spilsbury; S. Flores-Peinado; R. Ramírez-Necoechea; J. A. Ramírez-Telles; P. Mora-Medina; M. Pérez; E. Molina; E. Soní; M. E. Trujillo-Ortega	2012	Effects of long distance transportation and CO2 stunning on critical blood values in pigs	Meat Sci	10.1016/j.meatsci.2011.11.027

301.	C. Wang; Y. J. Chen; Y. J. Bi; P. Zhao; H. Q. Sun; J. H. Li; H. G. Liu; R. X. Zhang; X. Li; J. Bao	2020	Effects of Long-Term Gentle Handling on Behavioral Responses, Production Performance, and Meat Quality of Pigs	Animals	10.3390/ani10020330
302.	M. J. Ritter; M. Ellis; D. B. Anderson; S. E. Curtis; K. K. Keffaber; J. Killefer; F. K. McKeith; C. M. Murphy; B. A. Peterson	2009	Effects of multiple concurrent stressors on rectal temperature, blood acid-base status, and longissimus muscle glycolytic potential in market-weight pigs	Journal of Animal Science	10.2527/jas.2008-0874
303.	C. Leclercq; A. Prunier; E. Merlot	2014	Effects of neonatal surgical castration and immunocastration in male pigs on blood T lymphocytes and health markers	Animal	10.1017/s1751731114000445
304.	L. N. Edwards; T. Grandin; T. E. Engle; M. J. Ritter; A. A. Sosnicki; B. A. Carlson; D. B. Anderson	2010	The effects of pre-slaughter pig management from the farm to the processing plant on pork quality	Meat science	
305.	F. Mantis; I. Bizelis; G. K. Symeon; E. Rogdakis	2019	Effects of pre-slaughter short-term factors on pork quality	Animal Production Science	10.1071/an17665
306.	L. M. Rocha; A. M. Bridi; A. Foury; P. Mormède; A. V. Weschenfelder; N. Devillers; W. Bertoloni; L. Faucitano	2013	Effects of ractopamine administration and castration method on the response to preslaughter stress and carcass and meat quality in pigs of two Piétrain genotypes	J Anim Sci	10.2527/jas.2012-6058
307.	G. A. Carroll; L. A. Boyle; D. L. Teixeira; N. van Staaveren; A. Hanlon; N. E. O'Connell	2016	Effects of scalding and dehairing of pig carcasses at abattoirs on the visibility of welfare-related lesions	Animal	10.1017/s1751731115002037
308.	S. Conte; L. Faucitano; R. Bergeron; S. Torrey; H. W. Gonyou; T. Crowe; E. T. Tamminga; T. M. Widowski	2015	Effects of season, truck type, and location within truck on gastrointestinal tract temperature of market-weight pigs during transport	Journal of Animal Science	10.2527/jas.2015-9338
309.	S. M. Lee; J. Y. Kim; E. J. Kim	2012	Effects of Stocking Density or Group Size on Intake, Growth, and Meat Quality of Hanwoo Steers (Bos taurus coreanae)	Asian-Australasian Journal of Animal Sciences	10.5713/ajas.2012.12254
310.	K. J. Fiedler; R. L. Parsons; L. J. Sadler; S. T. Millman	2016	Effects of stocking rate on measures of efficacy and welfare during argon gas euthanasia of weaned pigs	Animal Welfare	10.7120/09627286.25.1.083
311.	K. J. Fiedler; R. L. Parsons; L. J. Sadler; S. T. Millman	2014	Effects of stocking rate on measures of efficacy and welfare during carbon dioxide gas euthanasia of young pigs	Animal Welfare	10.7120/09627286.23.3.309
312.	K. Rigalma; C. Duvaux-Ponter; V. Deiss; C. Charles; L. Deveaux; F. Deschamps; S. Roussel	2010	Effects of stray voltage on the physiology of stress, growth performance and carcass parameters in Romane male lambs	Small Ruminant Research	10.1016/j.smallrumres.2010.07.012
313.	M. S. Herskin; K. Thodberg; H. E. Jensen	2015	Effects of tail docking and docking length on neuroanatomical changes in healed tail tips of pigs	Animal	10.1017/s1751731114002857
314.	Y. Z. Li; H. F. Zhang; L. J. Johnston; W. Martin; J. D. Peterson; J. F. Coetzee	2017	Effects of tail docking and tail biting on performance and welfare of growing-finishing pigs in a confinement housing system	Journal of Animal Science	10.2527/jas2017.1571
315.	F. A. Dalla Costa; L. S. Lopes; O. A. Dalla Costa	2017	Effects of the Truck Suspension System on Animal Welfare, Carcass and Meat Quality Traits in Pigs	Animals (Basel)	10.3390/ani7010005
316.	S. P. Parois; A. W. Duttlinger; B. T. Richert; S. R. Lindemann; J. S. Johnson; J. N. Marchant-Forde	2020	Effects of Three Distinct 2-Week Long Diet Strategies After Transport on Weaned Pigs' Short and Long-Term Welfare Markers, Behaviors, and Microbiota	Frontiers in Veterinary Science	10.3389/fvets.2020.00140
317.	A. V. Weschenfelder; S. Torrey; N. Devillers; T. Crowe; A. Bassols; Y. Saco; M. Piñeiro; L. Saucier; L. Faucitano	2012	Effects of trailer design on animal welfare parameters and carcass and meat quality of three Pietrain crosses being transported over a long distance	J Anim Sci	10.2527/jas.2012-4676

318.	A. V. Weschenfelder; S. Torrey; N. Devillers; T. Crowe; A. Bassols; Y. Saco; M. Pineiro; L. Saucier; L. Faucitano	2013	Effects of trailer design on animal welfare parameters and carcass and meat quality of three Pietrain crosses being transported over a short distance	Livestock Science	10.1016/j.livsci.2013.07.004
319.	D. Bert; B. Sanne Van; B. Johan	2020	Effects of Transport and Lairage on the Skin Damage of Pig Carcasses	Animals, Vol 10, Iss 575, p	
320.	M. A. Sutherland; B. L. Backus; J. J. McGlone	2014	Effects of Transport at Weaning on the Behavior, Physiology and Performance of Pigs	Animals (Basel)	10.3390/ani4040657
321.	E. Wirthgen; S. Goumon; M. Kunze; C. Walz; M. Spitschak; A. Tuchscherer; J. Brown; C. Höflich; L. Faucitano; A. Hoeflich	2018	Effects of Transport Duration and Environmental Conditions in Winter or Summer on the Concentrations of Insulin-Like Growth Factors and Insulin-Like Growth Factor-Binding Proteins in the Plasma of Market-Weight Pigs	Front Endocrinol (Lausanne)	10.3389/fendo.2018.00036
322.	S. Goumon; J. A. Brown; L. Faucitano; R. Bergeron; T. M. Widowski; T. Crowe; M. L. Connor; H. W. Gonyou	2013	Effects of transport duration on maintenance behavior, heart rate and gastrointestinal tract temperature of market-weight pigs in 2 seasons	J Anim Sci	10.2527/jas.2012-6081
323.	Z. Zhu; Y. Chen; Z. Y. Huang; Y. Zhang; Q. Xu; Y. Y. Tong; F. Zhai; G. B. Chang; G. H. Chen	2014	Effects of transport stress and rest before slaughter on blood parameters and meat quality of ducks	Canadian Journal of Animal Science	10.4141/cjas-2014-017
324.	M. B. Scheeren; H. W. Gonyou; J. Brown; A. V. Weschenfelder; L. Faucitano	2014	Effects of transport time and location within truck on skin bruises and meat quality of market weight pigs in two seasons	Canadian Journal of Animal Science	10.4141/cjas2013-136
325.	C. S. Gajana; T. T. Nkukwana; U. Marume; V. Muchenje	2013	Effects of transportation time, distance, stocking density, temperature and lairage time on incidences of pale soft exudative (PSE) and the physico-chemical characteristics of pork	Meat Sci	10.1016/j.meatsci.2013.05.028
326.	G. Martelli; R. Boccuzzi; M. Grandi; G. Mazzone; G. Zaghini; L. Sardi	2010	The effects of two different light intensities on the production and behavioural traits of Italian heavy pigs	Berl Munch Tierarztl Wochenschr	
327.	M. John; S. Avi	2014	The Effects of Using a Ramp and Elevator to Load and Unload Trailers on the Behavior and Physiology of Piglets	Animals, Vol 4, Iss 3, Pp 535-	
328.	M. A. Sutherland; A. McDonald; J. J. McGlone	2009	Effects of variations in the environment, length of journey and type of trailer on the mortality and morbidity of pigs being transported to slaughter	Vet Rec	10.1136/vetrec.165.1.13
329.	N. Cobanovic; M. Boskovic; D. Vasilev; M. Dimitrijevic; N. Parunovic; J. Djordjevic; N. Karabasil	2016	Effects of various pre-slaughter conditions on pig carcasses and meat quality in a low-input slaughter facility	South African Journal of Animal Science	10.4314/sajas.v46i4.6
330.	N. N. Jiang; T. Xing; P. Wang; C. Xie; X. L. Xu	2015	Effects of Water-misting Sprays with Forced Ventilation after Transport during Summer on Meat Quality, Stress Parameters, Glycolytic Potential and Microstructures of Muscle in Broilers	Asian-Australasian Journal of Animal Sciences	10.5713/ajas.15.0152
331.	D. Valent; L. Arroyo; R. Peña; K. Yu; R. Carreras; E. Mainau; A. Velarde; A. Bassols	2017	Effects on pig immunophysiology, PBMC proteome and brain neurotransmitters caused by group mixing stress and human-animal relationship	PLoS One	10.1371/journal.pone.0176928
332.	K. Walia; H. Lynch; J. Grant; G. Duffy; F. C. Leonard; P. G. Lawlor; G. E. Gardiner	2017	The efficacy of disinfectant misting in the lairage of a pig abattoir to reduce Salmonella and Enterobacteriaceae on pigs prior to slaughter	Food Control	10.1016/j.foodcont.2016.12.028
333.	J. Wang	2020	Efficacy of the Bartha-K61 vaccine and a gE_/gI_/TK_ prototype vaccine against variant porcine pseudorabies virus (vPRV) in piglets with sublethal challenge of vPRV	Research in veterinary science. 2020 Feb., v. 128	

334.		2014	EFSA recommends a toolbox of indicators for monitoring welfare at slaughter	Vet Rec	10.1136/vr.g212
335.	U. Neudorf; C. Müntjes; T. Konorza; H. Kälsch; P. Kahlert; G. Kaiser; A. Wissmann; C. Krüger; M. Kästner; M. Sigler; M. Schneider	2013	Ein _Baby-Stent_ für Neugeborene und das weitere Leben _ Ergebnisse der Tierversuche	The Thoracic and Cardiovascular Surgeon	
336.	E. Spindler; S. Klein; M. Erhard; S. Reese; D. Patzkéwitsch	2018	Eine alternative Abferkelbucht im Feldversuch _ direkter Vergleich zweier Abferkelsysteme	Tierärztliche Praxis Ausgabe G: Großtiere / Nutztiere	
337.	C. Otten; A. Berk; S. Dänicke	2013	Einfluss von Lysin- und Energiegehalt des Futters auf freiwillige Futteraufnahme und Wachstumsleistung von Ebern und Kastraten		
338.	M. von Wenzlawowicz	2009	Electrical stunning of sows and sheep	Deutsche Tierarztliche Wochenschrift	10.2376/0341-6593-116-107
339.	M. von Wenzlawowicz; R. Holmes; I. Schwarzlose; B. Maurer; M. Marahrens; M. Bucher; C. Opitz	2017	Elektrobetäubung beim Schwein _ eine Gratwanderung zwischen Tierschutz und Tierquälerei		
340.	I. Reimert; J. E. Bolhuis; B. Kemp; T. B. Rodenburg	2015	Emotions on the loose: emotional contagion and the role of oxytocin in pigs	Animal Cognition	10.1007/s10071-014-0820-6
341.	P. Y. Decaudin; D. Raboisson; A. Waret-Szkuta	2017	End-Cycle Sow Carcass Condemnation in a French Slaughterhouse	Frontiers in Veterinary Science	10.3389/fvets.2017.00108
342.	S. Maekawa; R. Nomura; T. Murase; Y. Ann; M. Oeholm; M. Harada	2013	Endoscopic gallbladder stenting for acute cholecystitis: a retrospective study of 46 elderly patients aged 65 years or older	BMC Gastroenterol	10.1186/1471-230x-13-65
343.	A. Schweiger; J. Fischer; K. Troeger	2012	Entblutestich optimal ansetzen<br>Untersuchungen zu Art und Umfang von Gefäßdurchtrennungen entlang des Stichkanals von Schlachtschweinen		
344.	A. Schweiger; J. Fischer; K. Troeger	2013	Entblutung von Schlachtschweinen<br>anatomische Untersuchung der Stichstelle		
345.	G. Bee; P. Chevillon; M. Bonneau	2015	Entire male pig production in Europe	Animal Production Science	10.1071/an15279
346.	A. Schütz; W. I. Sonntag; A. Spiller	2019	Environmental Enrichment in pig husbandry<br>Consumer comparative assessment of different housing elements based on a pictorial survey		
347.	A. Enz; G. Schüpbach-Regula; R. Bettschart; E. Fuschini; E. Bürgi; X. Sidler	2013	Erfahrungen zur Schmerzausschaltung bei der Ferkelkastration in der Schweiz Teil 1: Inhalationsanästhesie	Schweizer Archiv fur Tierheilkunde	
348.		2015	Erzeugung. Schweinefleisch. Dänische Schweinebranche nutzt internationale Standards für Transparenz bei Tierwohl, Qualität und Lebensmittelsicherheit	Fleischwirtschaft	
349.	J. McGlone; A. Johnson; A. Sapkota; R. Kephart	2014	Establishing Bedding Requirements during Transport and Monitoring Skin Temperature during Cold and Mild Seasons after Transport for Finishing Pigs	Animals (Basel)	10.3390/ani4020241
350.	K. Rebecca; J. Anna; S. Avi; S. Kenneth; M. John	2014	Establishing Bedding Requirements on Trailers Transporting Market Weight Pigs in Warm Weather	Animals, Vol 4, Iss 3, Pp 476-	
351.	K. Rebecca; J. Anna; S. Avi; S. Kenneth; M. John	2014	Establishing Sprinkling Requirements on Trailers Transporting Market Weight Pigs in Warm and Hot Weather	Animals, Vol 4, Iss 2, Pp 164-	

352.	J. McGlone; A. Sapkota; A. Johnson; R. Kephart	2014	Establishing Trailer Ventilation (Boarding) Requirements for Finishing Pigs during Transport	Animals (Basel)	10.3390/ani4030515
353.	T. Lindström; S. A. Sisson; S. S. Lewerin; U. Wennergren	2010	Estimating animal movement contacts between holdings of different production types	Prev Vet Med	10.1016/j.prevetmed.2010.03.002
354.	S. Yadav; H.-Y. Weng	2017	Estimating the scale of adverse animal welfare consequences of movement restriction and mitigation strategies in a classical swine fever outbreak	BMC veterinary research	
355.	K. E. Wurtz; J. M. Siegford; R. O. Bates; C. W. Ernst; J. P. Steibel	2017	Estimation of genetic parameters for lesion scores and growth traits in group-housed pigs	Journal of Animal Science	10.2527/jas2017.1757
356.	S. Harley; S. J. More; N. E. O'Connell; A. Hanlon; D. Teixeira; L. Boyle	2012	Evaluating the prevalence of tail biting and carcase condemnations in slaughter pigs in the Republic and Northern Ireland, and the potential of abattoir meat inspection as a welfare surveillance tool	Vet Rec	10.1136/vr.100986
357.	K. Wadepohl; T. Blaha; D. Meemken	2020	Evaluation of a simplified Herd Health and Welfare Index for benchmarking in pig herds	Tierarztliche Praxis. Ausgabe G, Grosstiere/Nutztiere	
358.	L. Blömke; N. Volkmann; N. Kemper	2020	Evaluation of an automated assessment system for ear and tail lesions as animal welfare indicators in pigs at slaughter	Meat Sci	10.1016/j.meatsci.2019.107934
359.	S. E. O. Oliveira; F. A. Dalla Costa; T. J. Gibson; O. A. D. Costa; A. Coldebella; N. G. Gregory	2018	Evaluation of brain damage resulting from penetrating and non-penetrating stunning in Nelore Cattle using pneumatically powered captive bolt guns	Meat Sci	10.1016/j.meatsci.2018.07.016
360.	N. Kells; N. Beausoleil; C. Johnson; M. Sutherland	2018	Evaluation of Different Gases and Gas Combinations for On-Farm Euthanasia of Pre-Weaned Pigs	Animals	10.3390/ani8030040
361.	K. Tereszkiewicz; K. Choroszy	2019	Evaluation of post-slaughter exsanguination of selected breeds of pigs	Indian Journal of Animal Research	10.18805/ijar.B-714
362.	E. Nannoni; G. Liuzzo; A. Serraino; F. Giacometti; G. Martelli; L. Sardi; M. Vitali; L. Romagnoli; E. Moscardini; F. Ostanello	2017	Evaluation of pre-slaughter losses of Italian heavy pigs	Animal Production Science	10.1071/an15893
363.	F. Vial; M. Reist	2014	Evaluation of Swiss slaughterhouse data for integration in a syndromic surveillance system	Bmc Veterinary Research	10.1186/1746-6148-10-33
364.	U. Yildiz; M. Saatci	2009	An Evaluation of the Welfare in the Large and Small Animal Transportations Made from Sarikamis	Kafkas Universitesi Veteriner Fakultesi Dergisi	
365.	F. Orford; E. A. Ford; S. N. Brown; J. McKinstry; P. J. Hadley; J. A. Lines; T. G. Knowles; S. B. Wotton	2016	The evaluation of two commercial electric sheep stunning systems: current applied and the effect on heart function	Animal Welfare	10.7120/09627286.25.3.331
366.	M. Krüger; N. Zinne; H. Höffler; R. Zhang; I. Kropivnitskaja; J. Schmitto; A. Ciubotaru; A. Haverich	2013	[Ex situ tracheobronchoplastic operations using the organ care system]	Chirurg	10.1007/s00104-012-2444-1
367.	A. Enz; G. Schüpbach-Regula; R. Bettschart; E. Fuschini; E. Bürgi; X. Sidler	2013	[Experiences with pain control during piglet castration in Switzerland Part 1: Inhalation anesthesia]	Schweiz Arch Tierheilkd	10.1024/0036-7281/a000530
368.	A. Enz; G. Schupbach-Regula; R. Bettschart; E. Fuschini; E. Burgi; X. Sidler	2013	Experiences with piglet castration under inhalational anaesthesia in Switzerland	Schweizer Archiv Fur Tierheilkunde	10.1024/0036-7281/a000530

369.	C. A. Schwartz; P. Haage; C. Hohl	2012	Experimentelle Frühdiagnostik der akuten mesenterialen Ischämie mittels diffusionsgewichteter MRT (DWI) und paralleler Bildgebung	RöFo - Fortschritte auf dem Gebiet der Röntgenstrahlen und der bildgebenden Verfahren	
370.	R. M. Ayyub	2015	Exploring perceptions of non-Muslims towards Halal foods in UK	British Food Journal	10.1108/bfj-07-2014-0257
371.	M. Shirali; S. Ponsuksili; E. Murani; P. F. Varley; J. Jensen; H. Reyer; K. Wimmers	2017	Exploring the genetics of feed efficiency and feeding behaviour traits in a pig line highly selected for performance characteristics	Molecular genetics and genomics, 1-11	
372.	M. A. Dolman; H. C. J. Vrolijk; I. J. M. d. Boer	2012	Exploring variation in economic, environmental and societal performance among Dutch fattening pig farms	Livestock Science	
373.	E. Negrato; G. Di Martino; M. Vascellari; G. Radaelli; K. Capello; F. Pascoli; D. Bertotto; L. Bonfanti	2013	Expression of heat shock protein 70 in the liver of extensively and intensively kept heavy pigs	Animal	10.1017/s1751731113000517
374.	E. Bao; K. R. Sultan; N. Bernhard; J. Hartung	2009	Expression of heat shock proteins in tissues from young pigs exposed to transport stress	Dtsch Tierarztl Wochenschr	
375.	D. L. Hoeksma; M. A. Gerritzen; A. M. Lokhorst; P. M. Poortvliet	2017	An extended theory of planned behavior to predict consumers' willingness to buy mobile slaughter unit meat	Meat Sci	10.1016/j.meatsci.2017.01.011
376.	X. Averos; T. G. Knowles; S. N. Brown; P. D. Warriss; L. F. Gosalvez	2010	Factors affecting the mortality of weaned piglets during commercial transport between farms	Veterinary Record	10.1136/vr.c6226
377.	Y. J. Xiong; R. S. Gates; A. R. Green-Miller	2018	Factors Affecting Trailer Thermal Environment Experienced by Market Pigs Transported in the US	Animals	10.3390/ani8110203
378.	C. E. Dewey; C. Haley; T. Widowski; Z. Poljak; R. M. Friendship	2009	Factors associated with in-transit losses of fattening pigs	Animal Welfare	
379.	R. S. E. Peden; F. Akaichi; I. Camerlink; L. A. Boyle; S. P. Turner	2019	Factors Influencing Farmer Willingness to Reduce Aggression between Pigs	Animals	10.3390/ani9010006
380.	A. Grumpel; J. Krieter; C. Veit; S. Dippel	2018	Factors influencing the risk for tail lesions in weaner pigs (Sus scrofa)	Livestock Science	10.1016/j.livsci.2018.09.001
381.	M. Sinclair; Y. Zhang; K. Descovich; C. J. C. Phillips	2020	Farm Animal Welfare Science in China-A Bibliometric Review of Chinese Literature	Animals	10.3390/ani10030540
382.	S. Mann; S. Beciu; G. A. Arghiroiu	2019	Farm animals against open borders: uncovering discrepancies between narratives and evidence regarding actors and motives in the animal trade	Ciencia Rural	10.1590/0103-8478cr20180567
383.	S. Schukat; A. Kuhlmann; H. Heise	2019	Fattening Pig Farmers' Intention to Participate in Animal Welfare Programs	Animals	10.3390/ani9121042
384.	X. Liu; H. Schmidt; D. Mörlein	2016	Feasibility of boar taint classification using a portable Raman device	Meat Sci	10.1016/j.meatsci.2016.02.015
385.	D. Sola-Oriol; J. Gasa	2017	Feeding strategies in pig production: Sows and their piglets	Animal Feed Science and Technology	10.1016/j.anifeedsci.2016.07.018
386.	E. Herbich; B. Heissenberger; A. Ladinig; A. Griessler; M. Ritzmann; C. Weissenbacher-Lang; I. Hennig-Pauka	2013	Feldstudie zur simultanen Vakzination gegen das porzine Circovirus Typ 2 und Mycoplasma hyopneumoniae	Tierärztliche Praxis G: Großtiere/Nutztiere	
387.	M. D. Guardia; J. Estany; J. Alvarez-Rodriguez; X. Manteca; M. Tor; M. A. Oliver; M. Gispert; A. Diestre	2012	A field assessment of the effect of pre-slaughter conditions and genetic-stress susceptibility on blood welfare indicators in pigs	Animal Welfare	10.7120/09627286.21.4.517

388.	K. Luuk; G. Victor; J. Rika	2017	A field efficacy and safety trial in the Netherlands in pigs vaccinated at 3 weeks of age with a ready-to-use porcine circovirus type 2 and Mycoplasma hyopneumoniae combined vaccine	Porcine Health Management, Vol 3, Iss 1, Pp 1-	
389.	D. Duivon; I. Corrégé; A. Hémonic; M. Rigaut; D. Roudaut; R. Jolie	2018	Field evaluation of piglet vaccination with a Mycoplasma hyopneumoniae bacterin as compared to a ready-to-use product including porcine circovirus 2 and M. hyopneumoniae in a conventional French farrow-to-finish farm	Porcine Health Manag	10.1186/s40813-017-0077-y
390.	E. Herbich; B. Heissenberger; A. Ladinig; A. Griessler; M. Ritzmann; C. Weissenbacher-Lang; I. Hennig-Pauka	2013	[Field trial on the simultaneous vaccination against porcine circovirus type 2 (PCV2) and Mycoplasma hyopneumoniae]	Tierarztl Prax Ausg G Grosstiere Nutztiere	
391.	J. G. M. Dos Reis; S. T. Machado; R. C. Santos; I. D. Naas; R. V. Oliveira	2015	FINANCIAL LOSSES IN PORK SUPPLY CHAIN: A STUDY OF THE PRE-SLAUGHTER HANDLING IMPACTS	Engenharia Agricola	10.1590/1809-4430-Eng.Agric.v35n1p163-170/2015
392.	M. van Son; M. P. Kent; H. Grove; R. Agarwal; H. Hamland; S. Lien; E. Grindflek	2017	Fine mapping of a QTL affecting levels of skatole on pig chromosome 7	BMC Genet	10.1186/s12863-017-0549-8
393.	M. S. Cockram	2019	Fitness of animals for transport to slaughter	Canadian Veterinary Journal-Revue Veterinaire Canadienne	
394.	N. Arzoomand; I. Vagsholm; R. Niskanen; A. Johansson; A. Comin	2019	Flexible distribution of tasks in meat inspection - A pilot study	Food Control	10.1016/j.foodcont.2019.03.010
395.	D. U. A. M. D. o. A. S. S. Mota-Rojas; W. Animal; H. U. A. M. D. o. A. S. S. Orozco-Gregorio; W. Animal; D. H. I. d. M. F. G. D. o. N. Villanueva-Garcia; H. U. A. M. I. D. o. R. B. Bonilla-Jaime; X. U. d. V. d. M.-L. V. E. M. D. o. S. S. Suarez-Bonilla; Health; R. I. N. d. C. M. y. N. S. Z. D. o. E. R. Hernandez-Gonzalez; R. Animal; P. U. A. M. D. o. A. S. S. Roldan-Santiago; W. Animal; M. E. U. N. A. d. M. D. o. A. M. Trujillo-Ortega; Production	2011	Foetal and neonatal energy metabolism in pigs and humans: a review	Veterinarni Medicina - UZEI (Czech Republic)	
396.	H. Stein; J. Schulz; N. Kemper; A. Tichy; I. Krauss; C. Knecht; I. Hennig-Pauka	2016	Fogging low concentrated organic acid in a fattening pig unit - Effect on animal health and microclimate	Ann Agric Environ Med	10.5604/12321966.1226850
397.	K. Barington; H. E. Jensen; K. Skovgaard	2018	Forensic age determination of human inflicted porcine bruises inflicted within 10 h prior to slaughter by application of gene expression signatures	Res Vet Sci	10.1016/j.rvsc.2018.08.007
398.	K. Barington; H. E. Jensen	2013	Forensic cases of bruises in pigs	Vet Rec	10.1136/vr.101854
399.	L. Scherer; B. Tomasik; O. Rueda; S. Pfister	2018	Framework for integrating animal welfare into life cycle sustainability assessment	International Journal of Life Cycle Assessment	10.1007/s11367-017-1420-x
400.	J. Cheng; S. Janssens; N. Buys	2009	Full sib pens of pigs are not suitable to identify variance component of associative effect: a simulation study using Gibbs Sampling	BMC genetics	

401.	S. B. Damy; R. S. Camargo; R. Chammas; L. F. Figueiredo	2010	[Fundamental aspects on animal research as applied to experimental surgery]	Rev Assoc Med Bras (1992)	10.1590/s0104-42302010000100024
402.	J. J. Zonderland; M. B. M. Bracke; L. A. den Hartog; B. Kemp; H. A. M. Spoolder	2010	Gender effects on tail damage development in single- or mixed-sex groups of weaned piglets	Livestock Science	10.1016/j.livsci.2010.01.018
403.	L. T. U. d. S. A. s. M. V. a. B. T. Czister; S. Acatincai; E. N. Sossidou	2011	General knowledge of the romanian farmers about the farm animal welfare	Lucrari stiintifice. Seria Zootehnie - Universitatea de Stiinte Agricole si Medicina Veterinara Ion Ionescu de la Brad (Romania)	
404.	T. L. Passafaro; D. Van de Stroet; N. M. Bello; N. H. Williams; G. J. M. Rosa	2019	Generalized additive mixed model on the analysis of total transport losses of market-weight pigs1	J Anim Sci	10.1093/jas/skz087
405.		2015	Genetic association between leg conformation in young pigs and sow reproduction	Livestock science. 2015 Aug., v. 178	
406.	C. Dugué; A. Prunier; M. J. Mercat; M. Monziols; B. Blanchet; C. Larzul	2020	Genetic determinism of boar taint and relationship with growth traits, meat quality and lesions	Animal : an international journal of animal bioscience	
407.	A. Storskrubb; M. L. Sevón-Aimonen; P. Uimari	2010	Genetic parameters for bone strength, osteochondrosis and meat percentage in Finnish Landrace and Yorkshire pigs	Animal : an international journal of animal bioscience	
408.	K. M. Pramod; V. Roos; A. M. Herman; F. K. Egbert	2018	Genetic Selection to Enhance Animal Welfare Using Meat Inspection Data from Slaughter Plants	Animals, Vol 8, Iss 2, p	
409.	V. R. Gregersen; L. N. Conley; K. K. Sørensen; B. Guldbrandtsen; I. H. Velander; C. Bendixen	2012	Genome-wide association scan and phased haplotype construction for quantitative trait loci affecting boar taint in three pig breeds	BMC Genomics	10.1186/1471-2164-13-22
410.	A. Kerdsin; D. Takeuchi; A. Nuangmek; Y. Akeda; M. Gottschalk; K. Oishi	2020	Genotypic Comparison between Streptococcus suis Isolated from Pigs and Humans in Thailand	Pathogens	10.3390/pathogens9010050
411.	P. G. de Abreu; O. A. Dalla Costa; V. Feddern; N. Mores; A. Coldebella; C. M. C. Ramos	2016	Geostatistics applied to swine facilities equipped with evaporative cooling system	Revista Brasileira De Engenharia Agricola E Ambiental	10.1590/1807-1929/agriambi.v20n11p1014-1019
412.	I. J. H. Duncan; M. Park; A. E. Malleau	2012	Global Animal Partnership's 5-Step (TM) Animal Welfare Rating Standards: a welfare-labelling scheme that allows for continuous improvement	Animal Welfare	10.7120/096272812x13345905673926
413.	S. G. Gala; M. L. Crandall	2018	Global Collaboration to Modernize Advanced Trauma Life Support Training	Journal of surgical education	
414.	S. Harley; S. More; L. Boyle; N. O. Connell; A. Hanlon	2012	Good animal welfare makes economic sense: potential of pig abattoir meat inspection as a welfare surveillance tool	Ir Vet J	10.1186/2046-0481-65-11
415.	M. Holinger; B. Fruh; P. Stoll; M. Kreuzer; E. Hillmann	2018	Grass silage for growing-finishing pigs in addition to straw bedding: Effects on behaviour and gastric health	Livestock Science	10.1016/j.livsci.2018.10.012
416.	K. Barington; J. F. G. Agger; S. S. Nielsen; K. Dich-Jørgensen; H. E. Jensen	2016	Gross and histopathological evaluation of human inflicted bruises in Danish slaughter pigs	BMC veterinary research	

417.	M. Holinger; B. Fruh; E. Hillmann	2015	Group composition for fattening entire male pigs under enriched housing conditions-Influences on behaviour, injuries and boar taint compounds	Applied Animal Behaviour Science	10.1016/j.applanim.2015.01.016
418.	G. Martelli; E. Nannoni; M. Grandi; A. Bonaldo; G. Zaghini; M. Vitali; G. Biagi; L. Sardi	2015	Growth parameters, behavior, and meat and ham quality of heavy pigs subjected to photoperiods of different duration	J Anim Sci	10.2527/jas.2014-7906
419.	J. M. Martins; R. Fialho; A. Albuquerque; J. Neves; A. Freitas; J. T. Nunes; R. Charneca	2019	Growth, blood, carcass and meat quality traits from local pig breeds and their crosses	Animal : an international journal of animal bioscience	
420.		2016	Guidance on assessing the fitness of pigs for transport	The Veterinary record	
421.	S. More; D. Bicout; A. Botner; A. Butterworth; P. Calistri; K. Depner; S. Edwards; B. Garin-Bastuji; M. Good; C. G. Schmidt; M. A. Miranda; S. S. Nielsen; A. Velarde; H. H. Thulke; L. Sihvonen; H. Spoolder; J. A. Stegeman; M. Raj; P. Willeberg; C. Winckler; R. Marano; F. Verdonck; D. Candiani; V. Michel; E. P. A. H. Anim; Welf	2018	Guidance on the assessment criteria for applications for new or modified stunning methods regarding animal protection at the time of killing	Efsa Journal	10.2903/j.efsa.2018.5343
422.	D. C. Poole; S. W. Copp; T. D. Colburn; J. C. Craig; D. L. Allen; M. Sturek; D. S. O'Leary; I. H. Zucker; T. I. Musch	2020	Guidelines for animal exercise and training protocols for cardiovascular studies	Am J Physiol Heart Circ Physiol	10.1152/ajpheart.00697.2019
423.	C. Holling; K.-H. Tölle; G. Otto; T. Blaha	2016	Haltung von Schweinen mit nicht kupierten Schwänzen in konventionellen Betrieben	Tierärztliche Praxis G: Großtiere/Nutztiere	
424.	L. M. Rocha; A. Dionne; L. Saucier; E. Nannoni; L. Faucitano	2015	Hand-held lactate analyzer as a tool for the real-time measurement of physical fatigue before slaughter and pork quality prediction	Animal	10.1017/s1751731114002766
425.	M. da Costa; S. M. Huertas; A. C. Strappini; C. Gallo	2014	Handling and Transport of Cattle and Pigs in South America	Livestock Handling and Transport, 4th Edition	
426.	H.-P. Isabel; M. Anne; B. Till Robert; S. Horst; G. Martin; S. Jochen	2019	Haptoglobin and C-Reactive Protein_Non-specific Markers for Nursery Conditions in Swine	Frontiers in Veterinary Science, Vol	
427.	K. D. Vogel; G. Badtram; J. R. Claus; T. Grandin; S. Turpin; R. E. Weyker; E. Voogd	2011	Head-only followed by cardiac arrest electrical stunning is an effective alternative to head-only electrical stunning in pigs	J Anim Sci	10.2527/jas.2010-2920
428.	V. Vladimir; V. Eva; S. Zbynek; P. Annamaria	2020	The Health and Welfare of Pigs from the Perspective of Post Mortem Findings in Slaughterhouses	Animals, Vol 10, Iss 825, p	
429.	F. Nienhaus; D. Meemken; C. Schoneberg; M. Hartmann; T. Kornhoff; T. May; S. Heß; L. Kreienbrock; A. Wendt	2020	Health scores for farmed animals: Screening pig health with register data from public and private databases	PLoS One	10.1371/journal.pone.0228497
430.	M. Oster; E. Murani; S. Ponsuksili; R. B. D'Eath; S. P. Turner; G. Evans; L. Tholking; E. Kurt; R. Klont; A. Foury; P. Mormede; K. Wimmers	2014	Hepatic expression patterns in psychosocially high-stressed pigs suggest mechanisms following allostatic principles	Physiology & Behavior	10.1016/j.physbeh.2014.02.014
431.	G. K. Hybschmann; A. K. Ersbøll; H. Vigre; N. P. Baadsgaard; H. Houe	2011	Herd-level risk factors for antimicrobial demanding gastrointestinal diseases in Danish herds with finisher pigs: A register-based study	Prev Vet Med	10.1016/j.prevetmed.2010.10.005
432.	A. H. Stygar; I. Chantziaras; I. Toppari; D. Maes; J. K. Niemi	2020	High biosecurity and welfare standards in fattening pig farms are associated with reduced antimicrobial use	Animal	10.1017/s1751731120000828

433.	M. von Wenzlawowicz; R. Holmes; I. Schwarzlose; B. Maurer; M. Marahrens; M. Bucher; C. Opitz	2017	Hochvoltelektrobetäubung beim Schlachtschwein Problemstellung und Schlussfolgerungen für die Vollzugspraxis		
434.	X. M. Vilanova; N. De Briyne; B. Beaver; P. V. Turner	2019	Horse Welfare During Equine Chorionic Gonadotropin (eCG) Production	Animals	10.3390/ani9121053
435.	S. Parotat; K. von Holleben; S. Arnold; K. Troeger; E. Luecker	2016	Hot-water spraying is a sensitive test for signs of life before dressing and scalding in pig abattoirs with carbon dioxide (CO2) stunning	Animal	10.1017/s1751731115001573
436.	A. Laura; V. Daniel; C. Ricard; P. Raquel; S. Josefa; V. Antonio; B. Anna	2019	Housing and road transport modify the brain neurotransmitter systems of pigs Do pigs raised in different conditions cope differently with unknown environments?	PLoS ONE, Vol 14, Iss 1, p e	
437.	R. Carreras; E. Mainau; L. Arroyo; X. Moles; J. Gonzalez; A. Bassols; A. Dalmau; L. Faucitano; X. Manteca; A. Velarde	2016	Housing conditions do not alter cognitive bias but affect serum cortisol, qualitative behaviour assessment and wounds on the carcass in pigs	Applied Animal Behaviour Science	10.1016/j.applanim.2016.09.006
438.	M. S. Herskin; K. K. Fogsgaard; D. Erichsen; M. Bonnichsen; C. Gaillard; K. Thodberg	2016	Housing of Cull Sows in the Hours before Transport to the Abattoir-An Initial Description of Sow Behaviour While Waiting in a Transfer Vehicle	Animals (Basel)	10.3390/ani7010001
439.	F. A. Dalla Costa	2019	How do season, on-farm fasting interval and lairage period affect swine welfare, carcass and meat quality traits?	International journal of biometeorology. 2019 Nov., v. 63, no. 11	
440.	M. L. V. Larsen; M. Bertelsen; L. J. Pedersen	2017	How do stocking density and straw provision affect fouling in conventionally housed slaughter pigs?	Livestock Science	10.1016/j.livsci.2017.09.005
441.	G. Temple; S. Chelsey	2015	How Farm Animals React and Perceive Stressful Situations Such As Handling, Restraint, and Transport	Animals, Vol 5, Iss 4, Pp 1233-	
442.	P. H. Hemsworth; M. Rice; M. G. Karlen; L. Calleja; J. L. Barnett; J. Nash; G. J. Coleman	2011	Human-animal interactions at abattoirs: Relationships between handling and animal stress in sheep and cattle	Applied Animal Behaviour Science	10.1016/j.applanim.2011.09.007
443.	G. J. Coleman	2012	Human-animal relationships at sheep and cattle abattoirs	Animal welfare. 2012 June, v. 21, no. 2	
444.	P. M. Thornber; R. J. Rubira; D. K. Styles	2014	Humane killing of animals for disease control purposes	Revue Scientifique Et Technique-Office International Des Epizooties	10.20506/rst.33.1.2279
445.	D. S. Collins; R. J. Huey	2015	Humane slaughter	Gracey's Meat Hygiene, 11th Edition	
446.	S. Stoier; L. Lykke; L. O. Blaabjerg	2018	Humane slaughter techniques for pigs	Achieving Sustainable Production of Pig Meat, Vol 3: Animal Health and Welfare	10.19103/as.2016.0013.27
447.	R. S. Aline; F. Shannon Axiak; J. B. Ngaio; B. Charlotte; B.-W. Regula; P. Rebeca García; D. W. G. Huw; M. Michael; M. Robert; S. Tobias; J. T. Michael; V. T. Patricia; M. W. Daniel; C. G. Thomas	2019	Humanely Ending the Life of Animals Research Priorities to Identify Alternatives to Carbon Dioxide	Animals, Vol 9, Iss 11, p	

448.	R. Mazzette	2015	Hygiene and Welfare Evaluation of Pigs Slaughtered in Agritourisms	Italian journal of food safety. 2015 May 28, v. 4, no. 2	
449.	I. Nastasijevic; I. Tomasevic; N. Smigic; D. Milicevic; Z. Petrovic; I. Djekic	2016	Hygiene assessment of Serbian meat establishments using different scoring systems	Food Control	10.1016/j.foodcont.2015.10.034
450.	O. Oladipo Olufemi; A. Gabriel Olubayo; E. Benjamin Obukowho; A. Oyeduntan Adejoju; U. Emmanuel Chibuike	2015	Identifiation of predisposing and risk factors associated with gastric lesions in pigs	Asian Pacific Journal of Tropical Disease, Vol 5, Iss 10, Pp 825-	
451.	L. Sardi; A. Gastaldo; M. Borciani; A. Bertolini; V. Musi; G. Martelli; D. Cavallini; G. Rubini; E. Nannoni	2020	Identification of Possible Pre-Slaughter Indicators to Predict Stress and Meat Quality: A Study on Heavy Pigs	Animals (Basel)	10.3390/ani10060945
452.	P. Brandt	2013	Identification of post-mortem indicators of welfare of finishing pigs on the day of slaughter	Livestock science. 2013 Nov., v. 157, no. 2-3	
453.	G. A. Carroll; L. A. Boyle; A. Hanlon; M. A. Palmer; L. Collins; K. Griffin; D. Armstrong; N. E. O'Connell	2018	Identifying physiological measures of lifetime welfare status in pigs: exploring the usefulness of haptoglobin, C- reactive protein and hair cortisol sampled at the time of slaughter	Ir Vet J	10.1186/s13620-018-0118-0
454.	N. Cobanovic; S. D. Stankovic; M. Dimitrijevic; B. Suvajdzic; N. Grkovic; D. Vasilev; N. Karabasil	2020	Identifying Physiological Stress Biomarkers for Prediction of Pork Quality Variation	Animals	10.3390/ani10040614
455.	M. v. Wenzlawowicz	2012	Identifying reasons for stun failures in slaughterhouses for cattle and pigs: a field study	Animal welfare. 2012 June, v. 21, no. 2	
456.	R. Gamero-Negron; J. S. del Pulgar; J. Ventanas; C. Garcia	2015	Immune-spaying as an alternative to surgical spaying in Iberian x Duroc females: Effect on carcass traits and meat quality characteristics	Meat Science	10.1016/j.meatsci.2014.08.005
457.	L. Rydhmer	2010	Immunocastration reduces aggressive and sexual behaviour in male pigs	Animal : an international journal of animal bioscience. 2010 June, v. 4, issue 6	
458.	B. Sionek; W. Przybylski	2016	THE IMPACT OF ANTE- AND POST-MORTEM FACTORS ON THE INCIDENCE OF PORK DEFECTIVE MEAT - A REVIEW	Annals of Animal Science	10.1515/aoas-2015-0086
459.	J. d. Jonge; J. C. M. v. Trijp	2013	The impact of broiler production system practices on consumer perceptions of animal welfare	Poultry Science	
460.	C. Wenke; J. Pospiech; T. Reutter; B. Altmann; U. Truyen; S. Speck	2018	Impact of different supply air and recirculating air filtration systems on stable climate, animal health, and performance of fattening pigs in a commercial pig farm	PLoS One	10.1371/journal.pone.0194641
461.	B. Driessen; S. Van Beirendonck; J. Buyse	2020	The Impact of Grouping on Skin Lesions and Meat Quality of Pig Carcasses	Animals (Basel)	10.3390/ani10040544
462.	S. L. Walters; C. J. Torres-Urbano; L. Chichester; R. E. Rose	2012	The impact of huts on physiological stress: a refinement in post-transport housing of male guineapigs (Cavia porcellus)	Laboratory animals	
463.	G. Arlene; S. Mhairi; P. Glenna; P. Guilherme; M. Matthew; B. Brittany; M. John	2016	Impact of Providing Feed and/or Water on Performance, Physiology, and Behavior of Weaned Pigs during a 32-h Transport	Animals, Vol 6, Iss 5, p	
464.	G. Di Martino; A. Scollo; A. Garbo; F. Lega; A. L. Stefani; M. Vascellari; A. Natale; F. Zuliani; C. Zanardello; F. Tonon; L. Bonfanti	2017	Impact of sexual maturity on the welfare of immunocastrated v. entire heavy female pigs	Animal : an international journal of animal bioscience	

465.	M. S. Herskin; H. E. Jensen; A. Jespersen; B. Forkman; M. B. Jensen; N. Canibe; L. J. Pedersen	2016	Impact of the amount of straw provided to pigs kept in intensive production conditions on the occurrence and severity of gastric ulceration at slaughter	Res Vet Sci	10.1016/j.rvsc.2015.12.017
466.	M. Donovan; J. Lennox Anderson; F. Rohan; E. Carla; C. Angella; S. Stephen; C. Tazhmoye	2009	The Impact of the North Coast Highway on Socioeconomic Status and Family Life of Residents in Bogue Village, Jamaica	Asian Social Science, Vol 5, Iss	
467.	M. El Khasmi; Y. Chakir; R. Bargaa; K. Barka; I. Lektib; N. El Abbadi; A. Belhouari; B. Faye	2015	Impact of transport distance on stress biomarkers levels in dromedary camel (Camelus dromedarius)	Emirates Journal of Food and Agriculture	
468.	S. Støier; H. D. Larsen; M. D. Aaslyng; L. Lykke	2016	Improved animal welfare, the right technology and increased business	Meat Sci	10.1016/j.meatsci.2016.04.010
469.	C. Schumacher-Petersen; K. P. Hammelev; J. E. Flescher	2014	An improved method for lifting and transporting anesthetized pigs within an animal facility	Lab Anim (NY)	10.1038/laban.531
470.	N. Hakansson; P. Flisberg; B. Algers; A. Jonsson; M. Ronnqvist; U. Wennergren	2016	Improvement of animal welfare by strategic analysis and logistic optimisation of animal slaughter transportation	Animal Welfare	10.7120/09627286.25.2.255
471.	T. Grandin	2014	Improving Welfare and Reducing Stress on Animals at Slaughter Plants	Livestock Handling and Transport, 4th Edition	
472.		2012	In mehr Tierwohl und weniger Antibiotika liegt die Zukunft der Schweinefleischproduktion	Amtstierärztlicher Dienst und Lebensmittelkontrolle	
473.	P. M. K. Leat; C. Revoredo-Giha	2013	In search of differentiation and the creation of value: the quest of the Scottish pig supply chain	British Food Journal	10.1108/bfj-07-2013-0193
474.	S. Ylva	2019	In vitro fertilisation in domestic mammals_a brief overview	Upsala Journal of Medical Sciences, Vol 0, Iss 0, Pp 1-	
475.	A. Cerisuelo; M. D. Baucells; J. Gasa; J. Coma; D. Carrión; N. Chapinal; R. Sala	2009	Increased sow nutrition during midgestation affects muscle fiber development and meat quality, with no consequences on growth performance	J Anim Sci	10.2527/jas.2007-0677
476.	M. T. W. Verhoeven; M. A. Gerritzen; L. J. Hellebrekers; B. Kemp	2015	Indicators used in livestock to assess unconsciousness after stunning: a review	Animal	10.1017/s1751731114002596
477.	R. Zapf; U. Schultheiß; W. Achilles; L. Schrader; U. Knierim; H.-J. Herrmann; J. Brinkmann; C. Winckler	2015	Indikatoren für die betriebliche Eigenkontrolle auf Tiergerechtheit _ Beispiel Milchkühe ; Indicators for on-farm self-assessment of animal welfare _ Example dairy cows		
478.	S. Combes; G. Postollec; L. Cauquil; T. Gidenne	2010	Influence of cage or pen housing on carcass traits and meat quality of rabbit	Animal	10.1017/s1751731109991030
479.	F. Adalberto; P. Marina; M. Simona; T. Maria Federica	2010	Influence of diet and rearing system on heavy pig performance, carcass and meat quality	Italian Journal of Animal Science, Vol 8, Iss 1, Pp 23-	
480.	P. Miriam; V. Christian; F. Michaela; D. Georg	2020	Influence of Dietary Fiber on the Development of the Gastrointestinal Tract and the Performance of Gilts	Sustainability, Vol 12, Iss 4961, p	
481.	K. Scott	2009	Influence of different types of environmental enrichment on the behaviour of finishing pigs in two different housing systems : 3. Hanging toy versus rootable toy of the same material	Applied animal behaviour science. 2009 Jan. 31, v. 116, no. 2-4	
482.	D. B. de Koning; E. M. van Grevenhof; B. F. A. Laurenssen; P. R. van Weeren; W. Hazeleger; B. Kemp	2014	The influence of floor type before and after 10 weeks of age on osteochondrosis in growing gilts	Journal of animal science	

483.	K. Nakyinsige; A. Q. Sazili; I. Zulkifli; Y. M. Goh; F. Abu Bakar; A. B. Sabow	2014	Influence of gas stunning and halal slaughter (no stunning) on rabbits welfare indicators and meat quality	Meat Science	10.1016/j.meatsci.2014.05.017
484.	A. Prunier	2013	Influence of housing and season on pubertal development, boar taint compounds and skin lesions of male pigs	Animal. 2013 Dec., v. 7, no. 12	
485.	E. V. a. F. U. B. U. V. V. L. a. T. Voslarova; P. V. a. F. U. B. U. V. V. L. a. T. Chloupek; L. V. a. F. U. B. U. H. a. T. M. Steinhauser; J. K. V. S. p. J. K. B. Havlicek; V. V. a. F. U. B. U. V. V. L. a. T. Vecerek	2010	Influence of housing system and number of transported animals on transport-induced mortality in slaughter pigs	Acta Veterinaria (Czech Republic)	
486.	A. K. Albrecht; E. G. Beilage; E. Kanitz; B. Puppe; I. Traulsen; J. Krieter	2012	Influence of immunisation against GnRF on agonistic and mounting behaviour, serum testosterone concentration and body weight in male pigs compared with boars and barrows	Applied Animal Behaviour Science	10.1016/j.applanim.2012.02.019
487.	S. Goumon	2017	Influence of loading handling and facilities on the subsequent response to pre-slaughter stress in pigs	Livestock science. 2017 June, v. 200	
488.	N. Čobanović	2016	The Influence of Pre-Mortem Conditions on Pale, Soft and Exudative (PSE) and Dark, Firm and Dry (DFD) Pork Meat	Acta veterinaria. 2016 June 28, v. 66, no. 2	
489.	D. Alvarez; M. D. Garrido; S. Banon	2009	Influence of Pre-Slaughter Process on Pork Quality: An Overview	Food Reviews International	10.1080/87559120902956216
490.	B. Lebret; P. Ecolan; N. Bonhomme; K. Meteau; A. Prunier	2015	Influence of production system in local and conventional pig breeds on stress indicators at slaughter, muscle and meat traits and pork eating quality	Animal	10.1017/s1751731115000609
491.	A. Rybarczyk; T. Karamucki; R. Drozd; D. Polasik; A. Lupkowska; A. Michalecka	2015	Influence of selected factors upon the blood loss from the carcasses of pigs free of the stress susceptibility gene (RYR1(T))	Animal Science Papers and Reports	
492.	R. Thomsen; S. A. Edwards; T. Rousing; R. Labouriau; J. T. Sørensen	2016	Influence of social mixing and group size on skin lesions and mounting in organic entire male pigs	Animal : an international journal of animal bioscience	
493.	H. Lee; C. Perkins; H. Gray; S. Hajat; M. Friel; R. P. Smith; S. Williamson; P. Edwards; L. M. Collins	2020	Influence of temperature on prevalence of health and welfare conditions in pigs: time-series analysis of pig abattoir inspection data in England and Wales	Epidemiol Infect	10.1017/s0950268819002085
494.	S. Zoels; S. Reiter; M. Ritzmann; C. Weiß; J. Numberger; A. Schütz; P. Lindner; V. Stefanski; U. Weiler	2020	Influences of Immunocastration on Endocrine Parameters, Growth Performance and Carcass Quality, as Well as on Boar Taint and Penile Injuries	Animals (Basel)	10.3390/ani10020346
495.	R. Roberto de Oliveira; B. William; C. Gerusa da Silva Salles; J. João Garcia Caramori; O. Vivian Christina da Costa; S. Gustavo de Sousa e; C. Raquel Aparecida Salles da	2010	Influência da distância no bem estar e qualidade de carne de suínos transportados em Mato Grosso Influence of distance on the welfare and meat quality of pigs transported in Mato Grosso	Revista Brasileira de Saúde e Produção Animal, Vol 11, Iss	
496.	R. B. D'Eath; G. Arnott; S. P. Turner; T. Jensen; H. P. Lahrmann; M. E. Busch; J. K. Niemi; A. B. Lawrence; P. Sandoe	2014	Injurious tail biting in pigs: how can it be controlled in existing systems without tail docking?	Animal	10.1017/s1751731114001359
497.	T. Mariam El; M. Carla Forte Maiolino	2019	Injury and condemnation data of pigs at slaughterhouses with federal inspection in the State of Paraná, Brazil, as indicators of welfare during transportation	Ciência Rural, Vol 49, Iss	

498.	E. Razzuoli; E. Olzi; P. Cala; S. Cafazzo; D. Magnani; A. Vitali; N. Lacetera; L. Archetti; F. Lazzara; A. Ferrari; L. N. Costa; M. Amadori	2016	Innate immune responses of young bulls to a novel environment	Veterinary Immunology and Immunopathology	10.1016/j.vetimm.2016.02.014
499.	S. Ponsuksili; M. Zebunke; E. Murani; N. Trakooljul; J. Krieter; B. Puppe; M. Schwerin; K. Wimmers	2015	Integrated Genome-wide association and hypothalamus eQTL studies indicate a link between the circadian rhythm-related gene PER1 and coping behavior	Sci Rep	10.1038/srep16264
500.	S. T. Machado; R. C. Santos; F. R. Caldara; M. C. Goncalves; I. D. Naas	2014	INTEGRATED MULTIVARIATE ANALYSIS TO EVALUATE EFFECTS OF PRESLAUGHTER HANDLING ON PORK QUALITY	Engenharia Agricola	10.1590/s0100-69162014000300007
501.	E. Wirthgen; M. Kunze; S. Goumon; C. Walz; C. Höflich; M. Spitschak; J. Brenmoehl; E. Kanitz; M. Tuchscherer; W. Otten; U. Gimsa; P. Schön; C. Manteuffel; A. Tuchscherer; R. Pfuhl; C. C. Metges; B. Stabenow; S. Erdmann; K. Schluricke; L. Faucitano; A. Hoeflich	2017	Interference of stress with the somatotropic axis in pigs - lights on new biomarkers	Sci Rep	10.1038/s41598-017-11521-5
502.	T. B. Jensen	2010	The interrelationships between clinical signs and their effect on involuntary culling among pregnant sows in group-housing systems	Animal : an international journal of animal bioscience. 2010 Nov., v. 4, issue 11	
503.		2015	Interview mit Wissenschaftlerin Sabine Dippel über die Entwicklung eines Tierwohl-Kriteriums zum Ringelschwanz		
504.	A. Haigh	2019	An investigation into the effectiveness of compressed straw blocks in reducing abnormal behaviour in growing pigs	Animal. 2019 Nov., v. 13, no. 11	
505.	G. Limon; E. A. Gonzales-Gustavson; T. J. Gibson	2016	Investigation Into the Humaneness of Slaughter Methods for Guinea Pigs (Cavia porcelus) in the Andean Region	Journal of applied animal welfare science : JAAWS	
506.	T. M. Seeiso; C. M. E. McCrindle	2009	An investigation of the quality of meat sold in Lesotho	Journal of the South African Veterinary Association, Vol 80, Iss 4, Pp 237-	
507.	L. Faucitano	2010	Invited review: Effects of lairage and slaughter conditions on animal welfare and pork quality	Canadian journal of animal science. 2010 Dec., v. 90, no. 4	
508.	P. Simons	2016	Ir Douwe Ehlhardt 1936 _ 2016	World's poultry science journal. 2016 May 26, v. 72, no. 2	
509.	A. Haigh; K. O'Driscoll	2019	Irish pig farmer's perceptions and experiences of tail and ear biting	Porcine Health Management	10.1186/s40813-019-0135-8
510.	E. Goyena; R. R. de Ybanez; C. Martinez-Carrasco; A. Saez-Acosta; G. Ramis; A. Torrecillas; F. A. de Vega; R. Casais; J. M. Prieto; E. Berriatua	2015	Is Sarcoptes scabiei infection in pigs a major welfare concern? A quantitative assessment of its effect in the host's nocturnal rubbing and lying behavior	Journal of Veterinary Behavior-Clinical Applications and Research	10.1016/j.jveb.2014.10.003
511.	S. Schrammar	2012	Kampf gegen die Fleischfabriken<br>Tierschützer bringen eigenes Label für Fleisch auf den Markt		

512.	C. Holling; K. H. Tölle; G. Otto; T. Blaha	2016	[Keeping pigs with undocked tails on conventionally producing farms. A feasibility study]	Tierarztl Prax Ausg G Grosstiere Nutztiere	10.15653/tpg-160025
513.	M. J. Hotzel; S. M. Mota; C. B. Ludtke; R. Poletto	2018	Knowledge and attitudes of official inspectors at slaughterhouses in southern Brazil regarding animal welfare	Revista Brasileira De Zootecnia-Brazilian Journal of Animal Science	10.1590/rbz4720170065
514.	I. Erian	2019	Knowledge of Stakeholders in the Livestock Industries of East and Southeast Asia about Welfare during Transport and Slaughter and Its Relation to Their Attitudes to Improving Animal Welfare	Animals. 2019 Mar. 19, v. 9, no. 3	
515.		2015	Kohlendioxid-Betäubung beim Schwein - Gibt es eine tierschutzgerechte Gasbetäubung?	Rundschau für Fleischhygiene und Lebensmittelüberwachung	
516.		2015	Konkretisierung tierbezogener Merkmale (Tierschutzindikatoren) nach § 11 (8) TierSchG für schweinehaltende Betriebe - Teil 2: Ferkelaufzucht und Mast	Rundschau für Fleischhygiene und Lebensmittelüberwachung	
517.		2015	Konkretisierung tierbezogener Merkmale (Tierschutzindikatoren) nach § 11(8) TierSchG für schweinehaltende Betriebe - Teil 1 - Allgemeines und Sauen	Rundschau für Fleischhygiene und Lebensmittelüberwachung	
518.		2017	LABEL-FIT<br>Projekt macht Schweinehaltung fit für das Tierschutz-Label		
519.	S. Weber; G. Das; K.-H. Waldmann; M. Gauly	2014	Labour time required for piglet castration with isoflurane-anaesthesia using shared and stationary inhaler devices	Berliner und Munchener tierarztliche Wochenschrift	
520.		2015	Länderreport Skandinavien. In den skandinavischen Ländern stehen die Themen Tierwohlsein und Tierschutz sowie Umweltschutz längst im Fokus. So wurden zum Beispiel im Rahmen des dänischen Tierschutzgipfels eine Reihe von Zielen zur Verbesserung des Tierwohls in der Schweinehaltung definiert, die in den kommenden Jahren umgesetzt werden sollen	Fleischwirtschaft	
521.	J.-J. Lu; W.-Z. Yuan; Y.-P. Zhu; S.-H. Hou; X.-J. Wang	2020	Latent pseudorabies virus infection in medulla oblongata from quarantined pigs	Transboundary and emerging diseases	
522.	H. Kongsted; J. T. Sørensen	2017	Lesions found at routine meat inspection on finishing pigs are associated with production system	Vet J	10.1016/j.tvjl.2017.04.016
523.	R. L. Langley; W. E. M. Morrow	2010	Livestock handling--minimizing worker injuries	Journal of agromedicine	
524.	F. M. Aarestrup	2015	The livestock reservoir for antimicrobial resistance: a personal view on changing patterns of risks, effects of interventions and the way forward	Philosophical Transactions of the Royal Society B-Biological Sciences	10.1098/rstb.2014.0085
525.	J. L. Hardstaff; B. Hasler; J. R. Rushton	2015	Livestock trade networks for guiding animal health surveillance	Bmc Veterinary Research	10.1186/s12917-015-0354-4
526.	G. C. Miranda-de la Lama; M. Villarroel; G. A. María	2014	Livestock transport from the perspective of the pre-slaughter logistic chain: a review	Meat Sci	10.1016/j.meatsci.2014.04.005

527.	G. C. Miranda-de la Lama; W. S. Sepúlveda; M. Villarroel; G. A. María	2011	Livestock vehicle accidents in Spain: causes, consequences, and effects on animal welfare	J Appl Anim Welf Sci	10.1080/10888705.2011.551622
528.	T. Grandin	2018	Livestock-handling assessments to improve the welfare of cattle, pigs and sheep	Animal Production Science	10.1071/an16800
529.	N. L. Berry; A. K. Johnson; J. Hill; S. Lonergan; L. A. Karriker; K. J. Stalder	2012	Loading gantry versus traditional chute for the finisher pig: effect on welfare at the time of loading and performance measures and transport losses at the harvest facility	J Anim Sci	10.2527/jas.2011-4973
530.	C. Kalbe; B. Puppe	2010	Long-term cognitive enrichment affects opioid receptor expression in the amygdala of domestic pigs	Genes, Brain and Behavior	
531.	A. Bulens; S. Van Beirendonck; J. Van Thielen; N. Buys; B. Driessen	2016	Long-term effects of straw blocks in pens with finishing pigs and the interaction with boar type	Applied Animal Behaviour Science	10.1016/j.applanim.2016.01.008
532.	M. M. J. van Riet; E. J. Bos; B. Ampe; P. Bikker; D. Vanhauteghem; F. Van Bockstaele; P. Cornillie; W. Van Den Broeck; G. Du Laing; D. Maes; F. A. M. Tuyttens; G. P. J. Janssens; S. Millet	2018	Long-term impact of zinc supplementation in sows: Impact on claw quality	Journal of Swine Health and Production	
533.	M. Cevallos-Almeida; C. Fablet; C. Houdayer; V. Dorenlor; F. Eono; M. Denis; A. Kerouanton	2019	Longitudinal study describing time to Salmonella seroconversion in piglets on three farrow-to-finish farms	Vet Rec Open	10.1136/vetreco-2018-000287
534.	P. Statham	2011	A longitudinal study of the effects of providing straw at different stages of life on tail-biting and other behaviour in commercially housed pigs	Applied animal behaviour science. 2011 Nov. 15, v. 134, no. 3-4	
535.	R. Sanne; B. Ilse van; M. Stephanie; J. v. d. S. Franz; E. N. Rebecca	2018	Low Birth Weight Impairs Acquisition of Spatial Memory Task in Pigs	Frontiers in Veterinary Science, Vol	
536.	M. Oster; C. Gerlinger; K. Heide; F. Just; L. Borgelt; P. Wolf; C. Polley; B. Vollmar; E. Murani; S. Ponsuksili; K. Wimmers	2018	Lower dietary phosphorus supply in pigs match both animal welfare aspects and resource efficiency	Ambio	10.1007/s13280-017-0969-8
537.	M. Holinger	2019	Magengeschwüre bei Schweinen verhindern		
538.	J. Gonzalez; J. Jaume; E. Fàbrega; M. Gispert; M. Gil; A. Oliver; P. Llonch; M. D. Guàrdia; C. E. Realini; J. Arnau; J. Tibau	2013	Majorcan Black Pig as a traditional pork production system: improvements in slaughterhouse procedures and elaboration of pork carpaccio as an alternative product	Meat Sci	10.1016/j.meatsci.2013.03.012
539.	T. Grandin	2013	Making slaughterhouses more humane for cattle, pigs, and sheep	Annu Rev Anim Biosci	10.1146/annurev-animal-031412-103713
540.		2016	Management Schwein. Haltung. Was kostet Tierwohl?	DLG-Mitteilungen	
541.		2016	Management Schwein. Interview »Echtes Tierwohl statt Kosmetik!«	DLG-Mitteilungen	
542.		2015	Management Schwein. Interview. Tierwohl als Wettbewerbsfaktor	DLG-Mitteilungen	
543.		2016	Management Schwein. Tierwohl. Emotionslos bewerten	DLG-Mitteilungen	
544.		2015	Management Schwein. Tierwohl. So einfach geht das nicht	DLG-Mitteilungen	
545.	S. L. Douglas; S. A. Edwards; I. Kyriazakis	2014	Management strategies to improve the performance of low birth weight pigs to weaning and their long-term consequences	Journal of Animal Science	10.2527/jas.2013-7388
546.	A. Valros; C. Munsterhjelm; L. Hnninen; T. Kauppinen; M. Heinonen	2016	Managing undocked pigs - on-farm prevention of tail biting and attitudes towards tail biting and docking	Porcine Health Management	10.1186/s40813-016-0020
547.	L. Hinrichsen	2010	Manufacturing technology in the Danish pig slaughter industry	Meat science	

548.		2016	Markenfleisch. Ein Forschungsprojekt zum Tierwohl zeigt die Weiterentwicklung eines Programms am Beispiel Gutfleisch Schwein	Fleischwirtschaft	
549.	M. Schroyen	2015	The MC4R c.893G>A mutation: A marker for growth and leanness associated with boar taint odour in Belgian pig breeds	Meat science. 2015 Mar., v. 101	
550.	L. Sardi; E. Nannoni; M. Grandi; G. Vignola; G. Zaghini; G. Martelli	2012	Meat and ham quality of Italian heavy pigs subjected to different illumination regimes	Berliner Und Munchener Tierarztliche Wochenschrift	10.2376/0005-9366-125-463
551.	M. Pineiro	2009	Meat juice: An alternative matrix for assessing animal health by measuring acute phase proteins. Correlations of pig-MAP and haptoglobin concentrations in pig meat juice and plasma	Research in veterinary science. 2009 Oct., v. 87, issue 2	
552.	L. Rey-Salgueiro; E. Martinez-Carballo; P. Fajardo; M. J. Chapela; M. Espiñeira; J. Simal-Gandara	2018	Meat quality in relation to swine well-being after transport and during lairage at the slaughterhouse	Meat science	
553.	M. D. Aaslyng; M. Hviid	2020	Meat quality in the Danish pig population anno 2018	Meat Science	10.1016/j.meatsci.2019.108034
554.		2018	Mehr Tierwohl für Geflügel, Milchkühe und Schweine - Forschungspreis der internationalen Gesellschaft für Nutztierhaltung vergeben	Tierärztliche Umschau	
555.		2019	Mehr Tierwohl für Schweine. Buchten sinnvoll einrichten	DGS	
556.	K. J. Bosman; M. C. Mourits; A. G. Oude Lansink; H. W. Saatkamp	2013	Minimization of the impact of Aujeszky's disease outbreaks in The Netherlands: a conceptual framework	Transbound Emerg Dis	10.1111/j.1865-1682.2012.01348.x
557.	C. Werner	2010	Mitochondrial respiratory activity in porcine longissimus muscle fibers of different pig genetics in relation to their meat quality	Meat science. 2010 May, v. 85, no. 1	
558.	A. Y. Adenkola; J. O. Ayo; A. K. B. Sackey; A. B. Adelaiye	2011	Modulatory role of ascorbic acid on behavioural responses of pigs transported by road during the harmattan season	Nigerian journal of physiological sciences : official publication of the Physiological Society of Nigeria	
559.		2016	Monatsschau. Einsatz für Tierwohl-Standards. Lagerbeihilfe für Schweinefleisch gefragt. EU erleichtert den Handel. Regelung zur Herkunft gekippt	Fleischwirtschaft	
560.	R. Gronskyte	2016	Monitoring pig movement at the slaughterhouse using optical flow and modified angular histograms	IAgrE Biosystems engineering. 2016 Jan., v. 141	
561.	H. P. Lahrmann; M. E. Busch; R. B. D'Eath; B. Forkman; C. F. Hansen	2017	More tail lesions among undocked than tail docked pigs in a conventional herd	Animal	10.1017/s1751731117000490
562.	Y. Zhao; H. Xin; J. D. Harmon; T. J. Baas	2016	MORTALITY RATE OF WEANED AND FEEDER PIGS AS AFFECTED BY GROUND TRANSPORT CONDITIONS	Transactions of the ASABE	10.13031/trans.59.11671
563.	S. I. Beia; L. Elefterie; R. A. Necula; V. E. Beia	2017	MORTALITY VERSUS PROFITABILITY IN A ROMANIAN SWINE FARM	Scientific Papers-Series Management Economic Engineering in Agriculture and Rural Development	

564.	S. Hintze	2013	Mounting behaviour in finishing pigs: Stable individual differences are not due to dominance or stage of sexual development	Applied animal behaviour science. 2013 July, v. 147, no. 1-2	
565.	N. van Staaveren; B. Doyle; A. Hanlon; L. A. Boyle	2019	Multi-Stakeholder Focus Groups on Potential for Meat Inspection Data to Inform Management of Pig Health and Welfare on Farm	Agriculture-Basel	10.3390/agriculture9020040
566.	M. A. Sutherland; P. J. Bryer; B. L. Davis; J. J. McGlone	2010	A multidisciplinary approach to assess the welfare of weaned pigs during transport at three space allowances	Journal of applied animal welfare science : JAAWS	10.1080/10888705.2010.483879
567.	I. Hennig-Pauka; J. Verspohl; P. Wohlsein; K. H. Waldmann; M. Wendt	2012	Multifactorial respiratory tract disease in fattening pigs in an outside climate house	Praktische Tierarzt	
568.	M. J. Sturos; R. C. Robbins; R. Moreno; B. L. McLamb; S. A. Rossow	2016	Narasin toxicosis in finishing pigs	Journal of Swine Health and Production	
569.		2019	Nationales Tierwohlkennzeichen für Schweine - Ein agrar- und tierethischer Kommentar	Rundschau für Fleischhygiene und Lebensmittelüberwachung	
570.	J. J. McGlone	2017	A natural interomone 2-methyl-2-butenal stimulates feed intake and weight gain in weaned pigs	Animal. 2017 Feb., v. 11, no. 2	
571.	R. Nitzsche	2012	Neugestaltung des Zutriebs zur und in die CO2-Betäubungsanlage bei der Schweineschlachtung<br>Verbesserung des Tierschutzes und der Fleischqualität		
572.	Z. Kallas; J. M. Gil; N. Panella-Riera; M. Blanch; G. M. L. Tacken; P. Chevillon; K. d. Roest; M. A. Oliver	2012	New EU Policies Towards Animal Welfare<br>The Relative Importance of Pig Castration	EuroChoices	
573.	S. Fourour; C. Fablet; V. Tocqueville; V. Dorenlor; F. Eono; E. Eveno; I. Kempf; C. Marois-Créhan	2018	A new multiplex real-time TaqMan(®) PCR for quantification of Mycoplasma hyopneumoniae, M. hyorhinis and M. flocculare: exploratory epidemiological investigations to research mycoplasmal association in enzootic pneumonia-like lesions in slaughtered pigs	J Appl Microbiol	10.1111/jam.13770
574.	J. W. M. Merks; P. K. Mathur; E. F. Knol	2012	New phenotypes for new breeding goals in pigs	Animal : an international journal of animal bioscience	
575.	F. Giametta; P. Catalano; A. Gentile; C. Perone; B. Bianchi	2017	A new supporting tool for pig handling in the breeding-slaughterhouse production chain	Vet Ital	10.12834/VetIt.975.5171.2
576.	M. Machtolf	2012	News from the international Meat Research Fat Substitution of Minced Products with Surimi-Optimization of the Delicacy of ecologically produced Pork Meat - Aspects of Animal Welfare in Slaughter Pigs	Fleischwirtschaft	
577.	A.-K. Rovers; I. B. Christoph-Schulz; D. Saggau; N. Brümmer	2018	Nicht vor meiner Haustür! Wo soll Nutztierhaltung stattfinden?		
578.	M. F. Iulietto; P. Sechi; C. M. Gaudenzi; L. Grispoldi; M. Ceccarelli; S. Barbera; B. T. Cenci-Goga	2018	Noise assessment in slaughterhouses by means of a smartphone app	Italian Journal of Food Safety	10.4081/ijfs.2018.7053

579.	T. E. Wolf; N. Mangwiro; F. O. Fasina; A. Ganswindt	2020	Non-invasive monitoring of adrenocortical function in female domestic pigs using saliva and faeces as sample matrices	Plos One	10.1371/journal.pone.0234971; 10.1371/journal.pone.0234971.r001; 10.1371/journal.pone.0234971.r002; 10.1371/journal.pone.0234971.r003; 10.1371/journal.pone.0234971.r004; 10.1371/journal.pone.0234971.r005; 10.1371/journal.pone.0234971.r006
580.	S. P. Shirazi-Beechey	2011	NONRUMINANT NUTRITION SYMPOSIUM: Intestinal glucose sensing and regulation of glucose absorption: Implications for swine nutrition	Journal of animal science. 2011 June, v. 89, no. 6	
581.	H. Swaby; N. G. Gregory	2012	A note on the frequency of gastric ulcers detected during post-mortem examination at a pig abattoir	Meat Sci	10.1016/j.meatsci.2011.06.015
582.	K. Mizuta; Y. Zhang; F. Mizuta; H. Hoshijima; T. Shiga; E. Masaki; C. W. Emala, Sr.	2015	Novel identification of the free fatty acid receptor FFAR1 that promotes contraction in airway smooth muscle	Am J Physiol Lung Cell Mol Physiol	10.1152/ajplung.00041.2015
583.	M. L. Seshoka; A. T. Kanengoni; F. K. Siebrits; K. H. Erlwanger	2013	The novel use of point of care" devices to evaluate transport duration on selected pork quality parameters"	South African Journal of Animal Science	10.4314/sajas.v43i5.9
584.	K. B. Kephart; M. T. Harper; C. R. Raines	2010	Observations of market pigs following transport to a packing plant	Journal of animal science	
585.	A. Friese; J. Schulz; L. Hoehle; A. Fetsch; B. A. Tenhagen; J. Hartung; U. Roesler	2012	Occurrence of MRSA in air and housing environment of pig barns		
586.	T. Grandin	2017	On-farm conditions that compromise animal welfare that can be monitored at the slaughter plant	Meat Science	10.1016/j.meatsci.2017.05.004
587.	R. T. Wilson	2017	THE ONE-HUMPED CAMEL IN UGANDA	Journal of Camel Practice and Research	10.5958/2277-8934.2017.00001.7
588.	A. Schweiger; J. Fischer; K. Troeger	2013	Optimal positioning of the sticking incision<br>investigation of the manner and extent of vessel lesions along the sticking canal in slaughter pigs		
589.	I. J. East; S. E. Roche; R. M. Wicks; K. de Witte; M. G. Garner	2014	Options for managing animal welfare on intensive pig farms confined by movement restrictions during an outbreak of foot and mouth disease	Prev Vet Med	10.1016/j.prevetmed.2014.10.002
590.	F. Mercati; C. Dall'Aglio; G. Acuti; V. Faeti; F. M. Tardella; C. Pirino; E. De Felice; P. Scocco	2020	Oregano Feed Supplementation Affects Glycoconjugates Production in Swine Gut	Animals	10.3390/ani10010149
591.	F. Leiber; A. Müller; V. Maurer; C. Schader; A. Bieber	2019	Organic dairy farming<br>towards sustainability		
592.	P. E. Etterlin	2015	Osteochondrosis, but not lameness, is more frequent among free-range pigs than confined herd-mates	Acta veterinaria scandinavica. 2015 Dec., v. 57, no. 1	

593.	R. Urban-Chmiel; R. Pyz-Lukasik; A. Dudzic; A. Wernicki	2014	Oxidative parameters and expression of 70KDa heat shock proteins in pig heart tissue after transport and slaughter	Polish Journal of Veterinary Sciences	10.2478/pjvs-2014-0062
594.	R. S. Dzikamunhenga; R. Anthony; J. Coetzee; S. Gould; A. Johnson; L. Karriker; J. McKean; S. T. Millman; S. R. Niekamp; A. M. O'Connor	2014	Pain management in the neonatal piglet during routine management procedures. Part 1: a systematic review of randomized and non-randomized intervention studies	Animal health research reviews	
595.	E. Olofsson; M. Noremark; S. S. Lewerin	2014	Patterns of between-farm contacts via professionals in Sweden	Acta Veterinaria Scandinavica	10.1186/s13028-014-0070-2
596.	R. Amezcua; D. L. Pearl; A. Martinez; R. M. Friendship	2011	Patterns of condemnation rates in swine from a federally inspected abattoir in relation to disease outbreak information in Ontario (2005-2007)	Can Vet J	
597.	V. Vanheukelom; B. Driessen; D. Maenhout; R. Geers	2011	Peat as environmental enrichment for piglets: The effect on behaviour, skin lesions and production results	Applied Animal Behaviour Science	10.1016/j.applanim.2011.06.010
598.	S. Reiter; S. Zöls; M. Ritzmann; V. Stefanski; U. Weiler	2017	Penile Injuries in Immunocastrated and Entire Male Pigs of One Fattening Farm	Animals (Basel)	10.3390/ani7090071
599.	M. I. Monge Garcia; Z. Jian; J. J. Settels; C. Hunley; M. Cecconi; F. Hatib; M. R. Pinsky	2018	Performance comparison of ventricular and arterial dP/dt(max) for assessing left ventricular systolic function during different experimental loading and contractile conditions	Crit Care	10.1186/s13054-018-2260-1
600.	O. A. Dalla Costa; F. D. Tavernari; L. D. Lopes; F. A. Dalla Costa; V. Feddern; G. de Lima	2020	Performance, carcass and meat quality of pigs submitted to immunocastration and different feeding programs	Research in Veterinary Science	10.1016/j.rvsc.2020.04.015
601.	F. Ryszka; B. Dolińska; M. Zieliński; D. Chyra; Z. Dobrzański	2013	Permeation of iodide from iodine-enriched yeast through porcine intestine	Acta Biochim Pol	
602.	N. De Briyne; C. Berg; T. Blaha; A. Palzer; D. Temple	2018	'Phasing out pig tail docking in the EU - present state, challenges and possibilities'	Porcine Health Manag	10.1186/s40813-018-0103-8
603.	D. M. Powell; C. P. Kozlowski; J. Clark; A. Seyfried; E. Baskir; A. D. Franklin	2020	Physical and Physiological Indicators of Welfare in Guinea Pigs (Cavia porcellus) Serving as Ambassador Animals	Animals	10.3390/ani10050815
604.	C. J. C. Phillips; M. K. Pines; M. Latter; T. Muller; J. C. Petherick; S. T. Norman; J. B. Gaughan	2012	Physiological and behavioral responses of sheep to gaseous ammonia	Journal of Animal Science	10.2527/jas.2011-4575
605.	C. J. C. Phillips; M. K. Pines; M. Latter; T. Muller; J. C. Petherick; S. T. Norman; J. B. Gaughan	2010	The physiological and behavioral responses of steers to gaseous ammonia in simulated long-distance transport by ship	Journal of Animal Science	10.2527/jas.2010-3089
606.	M. Liat; I.-S. Beata; K. Lee; S. M. Jerrold; M. Devorah; Y. Ahmad; N. Shiri; W. Nathalie; R. Olja; A. Weissam Abu; K. Eyal; R. Tal	2019	Physiological and economic benefits of abandoning invasive surgical procedures and enhancing animal welfare in swine production	Scientific Reports, Vol 9, Iss 1, Pp 1-	
607.	R. E. Doyle; J. Groat; P. C. Wynn; P. K. Holyoake	2015	Physiological and nonphysiological indicators of body condition score in weaner pigs	Journal of Animal Science	10.2527/jas.2014-8465
608.	K. Borzuta; D. Lisiak; P. Janiszewski; E. Grzeskowiak	2019	THE PHYSIOLOGICAL ASPECTS, TECHNIQUE AND MONITORING OF SLAUGHTER PROCEDURES AND THEIR EFFECTS ON MEAT QUALITY - A REVIEW	Annals of Animal Science	10.2478/aoas-2019-0039
609.	A. Valros; C. Munsterhjelm; E. Puolanne; M. Ruusunen; M. Heinonen; O. A. T. Peltoniemi; A. R. Pösö	2013	Physiological indicators of stress and meat and carcass characteristics in tail bitten slaughter pigs	Acta veterinaria Scandinavica	10.1186/1751-0147-55-75

610.	J. De la Fuente; M. Sanchez; C. Perez; S. Lauzurica; C. Vieira; E. G. de Chavarri; M. T. Diaz	2010	Physiological response and carcass and meat quality of suckling lambs in relation to transport time and stocking density during transport by road	Animal	10.1017/s1751731109991108
611.	R. Martinez-Rodriguez; P. Roldan-Santiago; H. Orozco-Gregorio; M. Trujillo-Ortega; P. Mora-Medina; M. Gonzalez-Lozano; M. Sanchez-Hernandez; H. Bonilla-Jaime; R. Garcia-Herrera; E. Hernandez-Trujillo; D. Mota-Rojas	2015	Physiological responses and blood gas exchange following long-distance transport of piglets weaned at different ages over unpaved or paved roads	Livestock Science	10.1016/j.livsci.2014.12.013
612.	N. S. Minka; J. O. Ayo	2009	Physiological responses of food animals to road transportation stress	African Journal of Biotechnology	
613.	J. S. Johnson; L. H. Baumgard	2018	PHYSIOLOGY SYMPOSIUM: Postnatal consequences of in utero heat stress in pigs	Journal of animal science	
614.	P. Roldan-Santiago; M. Trujillo-Ortega; F. Borderas-Tordesillas; R. Martínez-Rodríguez; P. Mora-Medina; S. Flores-Peinado; M. Sánchez-Hernández; R. García-Herrera; M. González-Lozano; D. Mota-Rojas	2015	Physiometabolic responses to road transport in weaned piglets for a short period and the effects of straw bedding	Anim Sci J	10.1111/asj.12324
615.	C. Correia-Gomes; R. P. Smith; J. I. Eze; M. K. Henry; G. J. Gunn; S. Williamson; S. C. Tongue	2016	Pig Abattoir Inspection Data: Can It Be Used for Surveillance Purposes?	PLoS One	10.1371/journal.pone.0161990
616.	N. van Staaveren; D. L. Teixeira; A. Hanlon; L. A. Boyle	2017	Pig carcass tail lesions: the influence of record keeping through an advisory service and the relationship with farm performance parameters	Animal	10.1017/s1751731116001117
617.	Y. Potes; M. Oliván; A. Rubio-González; B. de Luxán-Delgado; F. Díaz; V. Sierra; L. Arroyo; R. Peña; A. Bassols; J. González; R. Carreras; A. Velarde; M. Muñoz-Torres; A. Coto-Montes	2017	Pig cognitive bias affects the conversion of muscle into meat by antioxidant and autophagy mechanisms	Animal	10.1017/s1751731117000714
618.	R. Gronskyte	2015	Pig herd monitoring and undesirable tripping and stepping prevention	Computers and electronics in agriculture. 2015 Nov., v. 119	
619.	K. Lundstrom	2009	Pig meat quality from entire males	Animal : an international journal of animal bioscience. 2009 Nov., v. 3, no. 11	
620.	R. T. Wilson; E. S. Swai	2014	Pig Production in Tanzania<br>a Critical Review	Tropicultura, Vol 32, Iss 1, Pp 46-	
621.	O. O. Omotosho; B. O. Emikpe; O. T. Lasisi; O. V. Oladunjoye	2016	PIG SLAUGHTERING IN SOUTHWESTERN NIGERIA: PECULIARITIES, ANIMAL WELFARE CONCERNS AND PUBLIC HEALTH IMPLICATIONS	African journal of infectious diseases	
622.	E. Lambooij	2012	Pig welfare and what you can tell from the carcase	Veterinary Record	
623.	K. Zurbrigg; T. van Dreumel; M. Rothschild; D. Alves; R. Friendship; T. O'Sullivan	2017	Pig-level risk factors for in-transit losses in swine: a review	Canadian Journal of Animal Science	10.1139/cjas-2016-0193
624.	R. B. D_Eath	2010	Pigs_ aggressive temperament affects pre-slaughter mixing aggression, stress and meat quality	Animal : an international journal of animal bioscience. 2010 Apr., v. 4, issue 4	

625.	S. Lohmann; M. Eijken; U. Møldrup; B. K. Møller; J. Hunter; C. Moers; R. J. Ploeg; C. C. Baan; B. Jespersen; A. K. Keller	2019	A Pilot Study of Postoperative Animal Welfare as a Guidance Tool in the Development of a Kidney Autotransplantation Model With Extended Warm Ischemia	Transplant Direct	10.1097/txd.0000000000000941
626.	T. Zhang; W. Sun; J. Xue; J. Chen; Q. Jiang; L. Mou; H. Du	2019	Podocytic infolding glomerulopathy: two new cases with connective tissue disease and literature review	Clin Rheumatol	10.1007/s10067-019-04504-6
627.	M. Crina Laura; G. Alexandra; L. O. Valentin; B. Alexandru; T. C. Romeo	2014	Pollutants impact bioassay from waters and soils in Banat region	Medicamentul Veterinar , Vol 8, Iss 2, Pp 66-	
628.	T. H. Pihl; K. E. Illigen; G. Houen	2015	Polyclonal Peptide Antisera	Peptide Antibodies: Methods and Protocols	10.1007/978-1-4939-2999-3_11
629.	H. Benchaoui	2010	Population medicine and control of epidemics	Handb Exp Pharmacol	10.1007/978-3-642-10324-7_5
630.	W. L. Evangelista; I. de Fátima Tinoco; A. P. de Souza; L. J. Minette; F. da Costa Baeta; E. P. da Silva; L. A. de Oliveira	2012	Postural analysis of workers in a typical meat processing company in Brazil	Work	10.3233/wor-2012-0829-5392
631.	S. Lopez-Verge; J. Gasa; M. Farre; J. Coma; J. Bonet; D. Sola-Oriol	2018	Potential risk factors related to pig body weight variability from birth to slaughter in commercial conditions	Translational Animal Science	10.1093/tas/txy082
632.	S. Starosta	2015	Potenziale derzeitiger Befunderhebung - Verwendung der offiziellen Schlachttier- und Fleischuntersuchungsstatistik für einen Monitoring-Bericht der Tiergerechtheit		
633.	D. Bert; P. Ester; T. Jos Van; B. Sanne Van	2013	Practical handling skills during road transport of fattening pigs from farm to slaughterhouse<br>A brief review	Agricultural Sciences, Vol 04, Iss 12, Pp 756-	
634.	B. Fredriksen	2009	Practice on castration of piglets in Europe	Animal : an international journal of animal bioscience. 2009 Nov., v. 3, no. 11	
635.	H. Lehnert	2018	Praxisbetriebe zum Testen von Tierschutzindikatoren gesucht		
636.	J. Brinkmann; K. Cimer; S. March; S. Ivermeyer; A. Pelzer; U. Schultheiß; R. Zapf; C. Winckler	2019	Praxistaugliche Tierschutzindikatoren für die betriebliche Eigenkontrolle - ein Vorschlag für die deutsche Milch- und Mastrinderhaltung		
637.	L. Vermeulen; V. Van de Perre; L. Permentier; S. De Bie; G. Verbeke; R. Geers	2015	Pre-slaughter handling and pork quality	Meat Sci	10.1016/j.meatsci.2014.09.148
638.	L. Vermeulen; V. Van de Perre; L. Permentier; S. De Bie; G. Verbeke; R. Geers	2016	Pre-slaughter sound levels and pre-slaughter handling from loading at the farm till slaughter influence pork quality	Meat Sci	10.1016/j.meatsci.2016.02.007
639.	J. Camp Montoro; E. G. Manzanilla; D. Solà-Oriol; R. Muns; J. Gasa; O. Clear; J. A. Calderón Díaz	2020	Predicting Productive Performance in Grow-Finisher Pigs Using Birth and Weaning Body Weight	Animals (Basel)	10.3390/ani10061017
640.	P. Statham	2009	Predicting tail-biting from behaviour of pigs prior to outbreaks	Applied animal behaviour science. 2009 Dec., v. 121, no. 3-4	
641.	M. Janeczek; J. Świderski; A. Czerski; B. Żywicka; J. Bujok; M. Szymonowicz; E. Bilewicz; M. Dobrzyński; M. Korczyński; A. Chrószcz; Z. Rybak	2018	Preliminary Evaluation of Thulium Doped Fiber Laser in Pig Model of Liver Surgery	Biomed Res Int	10.1155/2018/3275284

642.	T. Blaha; C. Holling	2017	Present knowledge about and experiences with tail biting in pigs: Recommendations for phasing out routine tail docking	Berliner Und Munchener Tierarztliche Wochenschrift	10.2376/0005-9366-16045
643.	L. Faucitano	2018	Preslaughter handling practices and their effects on animal welfare and pork quality	J Anim Sci	10.1093/jas/skx064
644.	A. Kagambèga; T. Lienemann; L. Aulu; A. S. Traoré; N. Barro; A. Siitonen; K. Haukka	2013	Prevalence and characterization of Salmonella enterica from the feces of cattle, poultry, swine and hedgehogs in Burkina Faso and their comparison to human Salmonella isolates	BMC Microbiol	10.1186/1471-2180-13-253
645.	F. Gottardo; A. Scollo; B. Contiero; M. Bottacini; C. Mazzoni; S. A. Edwards	2017	Prevalence and risk factors for gastric ulceration in pigs slaughtered at 170 kg	Animal	10.1017/s1751731117000799
646.	M. Gareis; S. Oberlander; J. Zippliesl; S. Reese; B. Schade; B. Bohm; K. Schwaiger	2016	Prevalence of auxiliary bursae and injuries of claws in fattening pigs at time of slaughter-results of a study at four slaughterhouses	Berliner Und Munchener Tierarztliche Wochenschrift	
647.	A. Kagambèga; O. Martikainen; A. Siitonen; A. S. Traoré; N. Barro; K. Haukka	2012	Prevalence of diarrheagenic Escherichia coli virulence genes in the feces of slaughtered cattle, chickens, and pigs in Burkina Faso	Microbiologyopen	10.1002/mbo3.30
648.	M. Holinger; E. Hillmann; B. Fruh	2014	Prevalence of injuries and the resulting requirements in housing conditions of entire male pigs	Tieraerztliche Umschau	
649.	L. M. Pluym; A. Van Nuffel; S. Van Weyenberg; D. Maes	2013	Prevalence of lameness and claw lesions during different stages in the reproductive cycle of sows and the impact on reproduction results	Animal : an international journal of animal bioscience	
650.	M. Fondrevez; B. Minvielle; A. Labbé; C. Houdayer; N. Rose; E. Esnault; M. Denis	2014	Prevalence of pathogenic Yersinia enterocolitica in slaughter-aged pigs during a one-year survey, 2010-2011, France	Int J Food Microbiol	10.1016/j.ijfoodmicro.2013.12.027
651.	O. O. Omotosho; B. O. Emikpe; O. T. Lasisi; T. A. Jarikre	2016	Prevalence, distribution and pattern of gastric lesions in slaughtered pigs in south-western Nigeria	Onderstepoort J Vet Res	10.4102/ojvr.v83i1.1063
652.	J. Firn; R. Maggini; I. Chadès; S. Nicol; B. Walters; A. Reeson; T. G. Martin; H. P. Possingham; J.-B. Pichancourt; R. Ponce-Reyes; J. Carwardine	2015	Priority threat management of invasive animals to protect biodiversity under climate change	Global change biology	
653.	F. B. Norwood	2012	The private provision of animal-friendly eggs and porks	American journal of agricultural economics Vol. 94, No. 2 , p. 509-514	
654.	M. Bonneau; B. Lebret	2010	Production systems and influence on eating quality of pork	Meat Sci	10.1016/j.meatsci.2009.03.013
655.	K. H. d. Greef; H. M. Vermeer; H. W. J. Houwers; A. P. Bos	2011	Proof of Principle of the Comfort Class concept in pigs Experimenting in the midst of a stakeholder process on pig welfare	Livestock Science	
656.	C. Leeb; G. Rudolph; D. Bochicchio; G. Butler; S. Dippel; J. Y. Dourmad; S. Edwards; B. Früh; G. Illmann; A. Prunier; T. Rousing; C. Winckler	2014	ProPig - Betriebsspezifische Strategien zur Reduktion des Umwelteinflusses durch Verbesserung von Tiergesundheit, Wohlergehen und Ernährung von Bioschweinen		
657.	B. Padalino; R. Barrasso; D. Tullio; M. Zappaterra; L. N. Costa; G. Bozzo	2020	Protection of Animals during Transport: Analysis of the Infringements Reported from 2009 to 2013 during On-Road Inspections in Italy	Animals	10.3390/ani10020356
658.	D. Mouzo; R. Rodriguez-Vazquez; J. M. Lorenzo; D. Franco; C. Zapata; M. Lopez-Pedrouso	2020	Proteomic application in predicting food quality relating to animal welfare. A review	Trends in Food Science & Technology	10.1016/j.tifs.2020.03.029

659.	A. Marco-Ramell; A. M. de Almeida; S. Cristobal; P. Rodrigues; P. Roncada; A. Bassols	2016	Proteomics and the search for welfare and stress biomarkers in animal production in the one-health context	Mol Biosyst	10.1039/c5mb00788g
660.	C. Holling; E. G. Beilage; B. Vidondo; C. Nathues	2017	Provision of straw by a foraging tower effect on tail biting in weaners and fattening pigs	Porcine Health Management	10.1186/s40813-017-0052-7
661.	G. Cima	2011	Pushing for better welfare, pathology	J Am Vet Med Assoc	
662.	S. Hintze; E. Murphy; I. Bachmann; F. Wemelsfelder; H. Wurbel	2017	Qualitative Behaviour Assessment of horses exposed to short-term emotional treatments	Applied Animal Behaviour Science	10.1016/j.applanim.2017.06.012
663.	C. A. Stockman; T. Collins; A. L. Barnes; D. Miller; S. L. Wickham; D. T. Beatty; D. Blache; F. Wemelsfelder; P. A. Fleming	2011	Qualitative behavioural assessment and quantitative physiological measurement of cattle naive and habituated to road transport	Animal Production Science	10.1071/an10122
664.	T. Collins; C. A. Stockman; A. L. Barnes; D. W. Miller; S. L. Wickham; P. A. Fleming	2018	Qualitative Behavioural Assessment as a Method to Identify Potential Stressors during Commercial Sheep Transport	Animals	10.3390/ani8110209
665.	C. A. Stockman; T. Collins; A. L. Barnes; D. Miller; S. L. Wickham; E. Verbeek; L. Matthews; D. Ferguson; F. Wemelsfelder; P. A. Fleming	2014	Qualitative behavioural assessment of the motivation for feed in sheep in response to altered body condition score	Animal Production Science	10.1071/an13020
666.	A. Costa	2009	Quantification of Three-Dimensional Light Distribution in Pig Houses	Transactions of the ASABE. 2009 Sept-Oct, v. 52, no. 5	
667.	K. Zurbrigg; T. van Dreumel; M. F. Rothschild; D. Alves; R. Friendship; T. L. O'Sullivan	2018	Rapid Communication: Postmortem lesions and heart weights of in-transit-loss market pigs in Ontario	Journal of animal science	
668.	Q. Zheng; J. Lin; J. Huang; H. Zhang; R. Zhang; X. Zhang; C. Cao; C. Hambly; G. Qin; J. Yao; R. Song; Q. Jia; X. Wang; Y. Li; N. Zhang; Z. Piao; R. Ye; J. R. Speakman; H. Wang; Q. Zhou; Y. Wang; W. Jin; J. Zhao	2017	Reconstitution of UCP1 using CRISPR/Cas9 in the white adipose tissue of pigs decreases fat deposition and improves thermogenic capacity	Proc Natl Acad Sci U S A	10.1073/pnas.1707853114
669.	D. Bolaños-López; D. Mota-Rojas; I. Guerrero-Legarreta; S. Flores-Peinado; P. Mora-Medina; P. Roldan-Santiago; F. Borderas-Tordesillas; R. García-Herrera; M. Trujillo-Ortega; R. Ramírez-Necoechea	2014	Recovery of consciousness in hogs stunned with CO2: physiological responses	Meat Sci	10.1016/j.meatsci.2014.05.034
670.	M. Garcia-Celdran; G. Ramis; J. J. Quereda; E. Armero	2012	Reduction of transport-induced stress on finishing pigs by increasing lairage time at the slaughter house	Journal of Swine Health and Production	
671.	F. Chen; S. Zhang; Z. Deng; Q. Zhou; L. Cheng; S. W. Kim; J. Chen; W. Guan	2018	Regulation of amino acid transporters in the mammary gland from late pregnancy to peak lactation in the sow	J Anim Sci Biotechnol	10.1186/s40104-018-0250-4
672.	A. Agnese; R. Veronica; L. Fabio; D. O. Stefania; P. Vincenzo; C. Leonardo Nanni	2017	Relationship between Deck Level, Body Surface Temperature and Carcass Damages in Italian Heavy Pigs after Short Journeys at Different Unloading Environmental Conditions	Animals, Vol 7, Iss 2, p	
673.	C. Haley; C. E. Dewey; T. Widowski; R. Friendship	2010	Relationship between estimated finishing-pig space allowance and in-transit loss in a retrospective survey of 3 packing plants in Ontario in 2003	Can J Vet Res	

674.	P. Brandt; M. D. Aaslyng; T. Rousing; S. L. A. Schild; M. S. Herskin	2015	The relationship between selected physiological post-mortem measures and an overall pig welfare assessment from farm to slaughter	Livestock Science	10.1016/j.livsci.2015.07.007
675.	N. van Staaveren; A. P. Vale; E. G. Manzanilla; D. L. Teixeira; F. C. Leonard; A. Hanlon; L. A. Boyle	2016	Relationship between tail lesions and lung health in slaughter pigs	Prev Vet Med	10.1016/j.prevetmed.2016.03.004
676.	H. Hartmann; G. Rindermann; C. Siegling-Vlitakis; G. Arndt; K. Wolf; R. Fries	2010	Relationship between the response to the corneal reflex (depth of narcosis) and specific parameters in the slaughter blood of pigs narcotised with CO2	Animal Welfare	
677.	M. Dokmanovic; Z. M. Baltic; R. Markovic; M. Boskovic; J. Loncina; N. Glamoclija; M. Dordevic	2014	RELATIONSHIPS AMONG PRE-SLAUGHTER STRESS, RIGOR MORTIS, BLOOD LACTATE, AND MEAT AND CARCASS QUALITY IN PIGS	Acta Veterinaria-Beograd	10.2478/acve-2014-0013
678.	L. A. Gonzalez; K. S. Schwartzkopf-Genswein; M. Bryan; R. Silasi; F. Brown	2012	Relationships between transport conditions and welfare outcomes during commercial long haul transport of cattle in North America	Journal of Animal Science	10.2527/jas.2011-4796
679.	M. Becerril-Herrera; D. Mota-Rojas; I. G. Legarreta; A. S. de Aluja; C. Lemus-Flores; M. Gonzalez-Lozano; R. Ramirez-Necoechea; M. Alonso-Spilsbury	2009	Relevant aspects of swine welfare in transit	Veterinaria Mexico	
680.	A. Dalmau; E. Mainau; A. Velarde	2017	Reliability of Fear Assessment in Growing Pigs Exposed to a Novel Object Test in Commercial Conditions	Journal of Applied Animal Welfare Science	10.1080/10888705.2017.1310043
681.	J. A. Brown	2009	Reliability of temperament tests on finishing pigs in group-housing and comparison to social tests	Applied animal behaviour science. 2009 Apr., v. 118, no. 1-2	
682.	R. Iida; C. Piñeiro; Y. Koketsu	2020	Removal of sows in Spanish breeding herds due to lameness: Incidence, related factors and reproductive performance of removed sows	Preventive veterinary medicine	
683.	A. Dalmau; N. A. Geverink; A. v. Nuffel; L. v. Steenbergen; C. G. v. Reenen; V. Hautekiet; K. Vermeulen; A. Velarde; F. A. M. Tuyttens	2010	Repeatability of lameness, fear and slipping scores to assess animal welfare upon arrival in pig slaughterhouses	Animal	
684.	A. W. Duttlinger; K. R. Kpodo; D. C. Lay; B. T. Richert; J. S. Johnson	2019	Replacing dietary antibiotics with 0.20% l-glutamine in swine nursery diets: impact on health and productivity of pigs following weaning and transport1,2,3	J Anim Sci	10.1093/jas/skz098
685.	M. L. Schwarz; G. Reisig; A. Schütte; K. Becker; S. Serba; E. Forsch; S. Thier; S. Fickert; T. Lenz; C. Weiß; S. Hetjens; F. Bludau; F. Bothe; W. Richter; B. Schneider-Wald	2019	Report on a large animal study with Göttingen Minipigs where regenerates and controls for articular cartilage were created in a large number. Focus on the conditions of the operated stifle joints and suggestions for standardized procedures	PloS one	
686.	R. J. Cox; P. Nol; C. K. Ellis; M. V. Palmer	2019	Research with Agricultural Animals and Wildlife	Ilar j	10.1093/ilar/ilz006
687.	A. D. Zotte; Z. Princz; S. Metzger; A. Szabo; I. Radnai; E. Biro-Nemeth; Z. Orova; Z. Szendro	2009	Response of fattening rabbits reared under different housing conditions. 2. Carcass and meat quality	Livestock Science	10.1016/j.livsci.2008.07.021
688.	L. Soler; A. Gutiérrez; D. Escribano; M. Fuentes; J. J. Cerón	2013	Response of salivary haptoglobin and serum amyloid A to social isolation and short road transport stress in pigs	Res Vet Sci	10.1016/j.rvsc.2013.03.007
689.	M. A. Mitchell; H. A. M. Spoolder	2016	Results of a desk study on best practices for animal transport		
690.	T. J. Hook; J. M. Stookey; H. Wagner	2010	Rethinking cull boar transport	Can Vet J	

691.	K. Barington; K. Dich-Jørgensen; H. E. Jensen	2016	A retrospective study of forensic cases of skin ulcerations in Danish pigs from 2000 to 2014	Acta Vet Scand	10.1186/s13028-016-0229-0
692.	R. T. Wilson	2013	A review of pig pathology in Tanzania	Tropical animal health and production. 2013 Aug., v. 45, no. 6	
693.	F. C. Rioja-Lang; J. A. Brown; E. J. Brockhoff; L. Faucitano	2019	A Review of Swine Transportation Research on Priority Welfare Issues: A Canadian Perspective	Front Vet Sci	10.3389/fvets.2019.00036
694.	M. J. Ritter; A. K. Johnson; M. E. Benjamin; S. N. Carr; M. Ellis; L. Faucitano; T. Grandin; J. L. Salak-Johnson; D. U. Thomson; C. Goldhawk; M. S. Calvo-Lorenzo	2017	Review: Effects of Ractopamine Hydrochloride (Paylean) on welfare indicators for market weight pigs	Translational Animal Science	10.2527/tas2017.0060
695.	M. J. Ritter	2009	Review: Transport losses in market weight pigs: 1. A review of definitions, incidence, and economic impact	Professional animal scientist. 2009 Aug., v. 25, no. 4	
696.	A. Hill	2013	A risk and benefit assessment for visual-only meat inspection of indoor and outdoor pigs in the United Kingdom	Food control. 2013 Mar., v. 30, no. 1	
697.	C. Lupo; S. Le Bouquin; V. Allain; L. Balaine; V. Michel; I. Petetin; P. Colin; C. Chauvin	2010	Risk and indicators of condemnation of male turkey broilers in western France, February-July 2006	Prev Vet Med	10.1016/j.prevetmed.2010.01.011
698.	L. N. Costa; M. Sapino; S. Pippione; G. Mattalia; M. Saracco; S. Di Trani; C. Zanasi	2012	Risk assessment in stock calf transportation from France to Italy: the contribution of road inspections	Italian Journal of Animal Science	10.4081/ijas.2012.e6
699.	M. D. Guàrdia	2009	Risk assessment of skin damage due to pre-slaughter conditions and RYR1 gene in pigs	Meat science. 2009 Apr., v. 81, no. 4	
700.	L. Ellerbroek	2011	Risk based meat hygiene - new concepts for new risks	Zuchtungskunde	
701.	O. A. Dalla Costa; F. A. Dalla Costa; V. Feddern; L. D. S. Lopes; A. Coldebella; N. G. Gregory; G. J. M. M. de Lima	2019	Risk factors associated with pig pre-slaughtering losses	Meat science	
702.	K. S. Schwartzkopf-Genswein; L. Faucitano; S. Dadgar; P. Shand; L. A. González; T. G. Crowe	2012	Road transport of cattle, swine and poultry in North America and its impact on animal welfare, carcass and meat quality: a review	Meat Sci	10.1016/j.meatsci.2012.04.010
703.	B. L. Nielsen	2011	Road transport of farm animals: effects of journey duration on animal welfare	Animal. 2011 Mar., v. 5, no. 3	
704.	B. Padalino	2018	Road Transport of Farm Animals: Mortality, Morbidity, Species and Country of Origin at a Southern Italian Control Post	Animals. 2018 Sept. 17, v. 8, no. 9	
705.	J. Selene; J. A. Cheryl	2018	The role of dietary fibre in pig production, with a particular emphasis on reproduction	Journal of Animal Science and Biotechnology, Vol 9, Iss 1, Pp 1-	
706.	K. Yano; S. Takimoto; T. Motegi; T. Tomono; M. Hagiwara; Y. Idota; K. Morimoto; A. Takahara; T. Ogihara	2014	Role of P-glycoprotein in regulating cilnidipine distribution to intact and ischemic brain	Drug Metab Pharmacokinet	10.2133/dmpk.dmpk-13-rg-072
707.	H. S. Hasibuan	2014	The Role of Transit Oriented Development in Constructing Urban Environment Sustainability, the Case of Jabodetabek, Indonesia	Procedia Environmental Sciences. 2014, v. 20	
708.	M. Le Gall; A. Serena; H. Jørgensen; P. K. Theil; K. E. Bach Knudsen	2009	The role of whole-wheat grain and wheat and rye ingredients on the digestion and fermentation processes in the gut--a model experiment with pigs	Br J Nutr	10.1017/s0007114509990924

709.	M. B. M. Bracke	2009	Rope test may indicate efficacy of tail-biting treatments in growing pigs	Animal Welfare	
710.	M. Frisk; A. Jonsson; S. Sellman; P. Flisberg; M. Ronnqvist; U. Wennergren	2018	Route optimization as an instrument to improve animal welfare and economics in pre-slaughter logistics	Plos One	10.1371/journal.pone.0193223
711.	M. Hernández; J. Gómez-Laguna; I. Luque; S. Herrera-León; A. Maldonado; L. Reguillo; R. J. Astorga	2013	Salmonella prevalence and characterization in a free-range pig processing plant: tracking in trucks, lairage, slaughter line and quartering	Int J Food Microbiol	10.1016/j.ijfoodmicro.2012.12.026
712.	A. Valros; M. Heinonen	2015	Save the pig tail	Porcine Health Management	10.1186/2055-5660-1-2
713.		2016	Schlachttechnologie. Tierschutz und Technik gehören zusammen - Die IFFA 2016 zeigt technische Neuerungen bei der Ruhigstellung und Betäubung von Rind und Schwein	Fleischwirtschaft	
714.	B. Walker	2019	Schweine quer durch Europa		
715.		2015	Schweine. Auch bei Bioschweinen: Tierwohl im Fokus	DGS	
716.		2016	Schweine. Haltungsanforderungen heute und morgen. Wohl für das Tier, nicht für das Auge	DGS	
717.	F. W. Busse	2012	Schweinehaltung und Tierschutz in China	Amtstierärztlicher Dienst und Lebensmittelkontrolle	
718.		2015	Schweineproduktion. Tierwohl über das Mindestmaß hinaus verfolgen	Fleischwirtschaft	
719.	B. Algers; H. J. Blokhuis; A. Bøtner; D. M. Broom; P. Costa; M. Domingo; M. Greiner; J. Hartung; F. Koenen; C. Müller-Graf; R. Mohan; D. B. Morton; A. Osterhaus; D. U. Pfeiffer; R. Roberts; M. Sanaa; M. Salman; J. M. Sharp; P. Vannier; M. Wierup	2009	Scientific Opinion of the Panel on Animal Health and Welfare (AHAW) on a request from the Commission on porcine brucellosis (Brucella suis)		
720.	E. P. o. A. Health; Welfare	2014	Scientific Opinion on African swine fever		
721.	E. P. o. A. Health; Welfare	2013	Scientific Opinion on monitoring procedures at slaughterhouses for pigs	EFSA Journal, Vol 11, Iss	
722.	L. J. Keeling; A. Wallenbeck; A. Larsen; N. Holmgren	2012	Scoring tail damage in pigs: an evaluation based on recordings at Swedish slaughterhouses	Acta Vet Scand	10.1186/1751-0147-54-32
723.	R. Sommavilla; L. Faucitano; H. Gonyou; Y. Seddon; R. Bergeron; T. Widowski; T. Crowe; L. Connor; M. B. Scheeren; S. Goumon; J. Brown	2017	Season, Transport Duration and Trailer Compartment Effects on Blood Stress Indicators in Pigs: Relationship to Environmental, Behavioral and Other Physiological Factors, and Pork Quality Traits	Animals (Basel)	10.3390/ani7020008
724.	F. W. Bazer	2015	Select nutrients and their effects on conceptus development in mammals	Chinese Association of Animal Science and Veterinary Medicine Animal nutrition. 2015 Sept., v. 1, no. 3	
725.	N. Søren Saxmose; D. Matthew James; F. Björn; H. Hans	2017	Selection of Meat Inspection Data for an Animal Welfare Index in Cattle and Pigs in Denmark	Animals, Vol 7, Iss 12, p	

726.	P. A. Fleming; S. L. Wickham; C. A. Stockman; E. Verbeek; L. Matthews; F. Wemelsfelder	2015	The sensitivity of QBA assessments of sheep behavioural expression to variations in visual or verbal information provided to observers	Animal	10.1017/s1751731114003164
727.	Q. Wu; H. Zhang; H. Dong; K. Mehmood; Z. Chang; K. Li; S. Liu; M. U. Rehman; F. Nabi; M. T. Javed; H. Zhu; J. Li	2018	Seroprevalence and risk factors associated with Pseudorabies virus infection in Tibetan pigs in Tibet	BMC Vet Res	10.1186/s12917-018-1347-x
728.	C. Trevisan; E. M. Mkupasi; H. A. Ngowi; B. Forkman; M. V. Johansen	2016	Severe seizures in pigs naturally infected with Taenia solium in Tanzania	Vet Parasitol	10.1016/j.vetpar.2016.02.025
729.	R. H. Dwinger; E. Lambooij; I. Arendzen	2011	[A short overview of the European laws concerning animal welfare]	Tijdschr Diergeneeskd	
730.	M. Verdon; R. S. Morrison; M. Rice; K. L. Butler; P. H. Hemsworth	2017	The short-term behavioural response of sows, but not gilts, to a social stimulus is related to sow aggressiveness in groups	Behav Processes	10.1016/j.beproc.2017.04.013
731.	L. N. Costa	2009	Short-term stress: the case of transport and slaughter	Italian Journal of Animal Science	
732.	M. S. Herskin; M. D. Aaslyng; I. Anneberg; P. T. Thomsen; L. M. Gould; K. Thodberg	2020	Significant variation in the management of cull sows before transport for slaughter: results from a survey of Danish pig farmers	Vet Rec	10.1136/vr.105671
733.	E. Vidal; E. Tolosa; S. Espinar; B. P. de Val; M. Nofrarías; A. Alba; A. Allepuz; L. Grau-Roma; S. López-Soria; J. Martínez; M. L. Abarca; J. Castellà; X. Manteca; M. I. Casanova; M. Isidoro-Ayza; I. Galindo-Cardiel; S. Soto; R. Dolz; N. Majó; A. Ramis; J. Segalés; L. Mas; C. Chacón; L. Picart; A. Marco; M. Domingo	2016	Six-Year Follow-up of Slaughterhouse Surveillance (2008-2013): The Catalan Slaughterhouse Support Network (SESC)	Vet Pathol	10.1177/0300985815593125
734.	M. Bottacini; A. Scollo; S. A. Edwards; B. Contiero; M. Veloci; V. Pace; F. Gottardo	2018	Skin lesion monitoring at slaughter on heavy pigs (170 kg): Welfare indicators and ham defects	PLoS One	10.1371/journal.pone.0207115
735.	P. Rolzhauser; S. Wohlfahrt; J. Saffaf; L. Walter; A. Pahl; A. Hamedy; E. Lucker; K. Riehn	2019	The slaughter of pregnant breeding sows in Germany	Fleischwirtschaft	
736.	K. Śmiecińska	2011	Slaughter value, meat quality, creatine kinase activity and cortisol levels in the blood serum of growing-finishing pigs slaughtered immediately after transport and after a rest period	Polish journal of veterinary sciences. 2011 Aug. 2, v. 14, no. 1	
737.	K. Dorfler; M. Machtolf	2013	Slaughtering Technologies Animal Welfare requires new Solutions IFFA 2013: Developments in Cattle and Pig Slaughtering Technologies	Fleischwirtschaft	
738.	A. F. A. Pires; A. Peterson; J. N. Baron; R. Adams; B. Martínez-López; D. Moore	2019	Small-scale and backyard livestock owners needs assessment in the western United States	PloS one	
739.	B. Früh	2019	So sind Schweine auch im Freiland sicher		
740.	H. M. Nielsen; B. Ask; P. Madsen	2018	Social genetic effects for growth in pigs differ between boars and gilts	Genetics Selection Evolution	10.1186/s12711-018-0375-0
741.	I. B. Christoph-Schulz; A.-K. Rovers; N. Brümmer; D. Saggau	2017	SocialLab - Nutztierhaltung im Spiegel der Gesellschaft		
742.	L. Vermeulen; V. Van de Perre; L. Permentier; S. De Bie; G. Verbeke; R. Geers	2015	Sound levels above 85 dB pre-slaughter influence pork quality	Meat Sci	10.1016/j.meatsci.2014.10.025

743.	M. Heinonen; P. Bergman; M. Fredriksson-Ahomaa; A. M. Virtala; C. Munsterhjelm; A. Valros; C. Oliviero; O. Peltoniemi; O. Halli	2018	Sow mortality is associated with meat inspection findings	Livestock Science	10.1016/j.livsci.2017.12.011
744.	M. A. Sutherland; P. J. Bryer; B. L. Davis; J. J. McGlone	2009	Space requirements of weaned pigs during a sixty-minute transport in summer	J Anim Sci	10.2527/jas.2008-1078
745.		2018	Staatliches Tierwohllabel ohne Schweine?	DGS	
746.	B. Plav_ic; A. Rozstalnyy; J. Y. Park; V. Guberti; K. R. Depner; G. Torres	2019	Strategic challenges to global control of African swine fever		
747.	S. Lopez-Verge; J. Gasa; D. Temple; J. Bonet; J. Coma; D. Sola-Oriol	2018	Strategies to improve the growth and homogeneity of growing-finishing pigs: feeder space and feeding management	Porcine Health Management	10.1186/s40813-018-0090-9
748.	M. J. R. Paranhos da Costa	2012	Strategies to promote farm animal welfare in Latin America and their effects on carcass and meat quality traits	Meat science. 2012 Nov., v. 92, no. 3	
749.	K. D. C. Stärk	2014	Strengths and weaknesses of meat inspection as a contribution to animal health and welfare surveillance	Food control. 2014 May, v. 39	
750.	E. M. C. Terlouw; C. Bourguet; V. Deiss	2012	Stress at slaughter in cattle: role of reactivity profile and environmental factors	Animal Welfare	10.7120/096272812x13353700593482
751.	D. M. Rojas; P. R. Santiago; E. P. Pedraza; R. M. Rodriguez; E. Hernandez-Trujillo; M. E. T. Ortega	2014	Stress factors in weaned piglet	Veterinaria Mexico	
752.	P. Chloupek; E. Voslarova; J. Chloupek; I. Bedanova; V. Pistekova; V. Vecerek	2009	Stress in Broiler Chickens Due to Acute Noise Exposure	Acta Veterinaria Brno	10.2754/avb200978010093
753.	T. G. Knowles; P. D. Warriss; K. Vogel	2014	Stress Physiology of Animals During Transport	Livestock Handling and Transport, 4th Edition	
754.	N. B. Athayde; O. A. Dalla Costa; R. O. Roça; A. L. Guidoni; C. B. Ludtke; E. Oba; R. K. Takahira; G. J. M. M. Lima	2013	Stress susceptibility in pigs supplemented with ractopamine	Journal of animal science	
755.	S. Durham	2010	Stress: It's Not Just for You and Me	Agricultural research. 2010 Aug., v. 58, no. 7	
756.		2016	Stroh in der Schweinehaltung. Nachdem in vielen Medien häufig, zum Teil täglich, auf nicht tiergerechte Haltungsbedingungen hingewiesen wird, ist es notwendig, sich sofort und zukünftig mit besseren Haltungsbedingungen unserer Nutztiere, insbesondere bei Geflügel und Schwein, intensiv zu beschäftigen und Besserung zu erreichen. Die Grundsätze wie ein Tier zu halten ist, sind in § 2 Tierschutzgesetz (TSG) dargelegt; leider wird häufig dagegen verstoßen	Rundschau für Fleischhygiene und Lebensmittelüberwachung	
757.	K. M. D. Rutherford; C. S. Thompson; J. R. Thomson; A. B. Lawrence; E. O. Nielsen; M. E. Busch; S. Haugegaard; P. Sandoe	2018	A study of associations between gastric ulcers and the behaviour of finisher pigs	Livestock Science	10.1016/j.livsci.2018.03.013
758.	B. T. Spencer; C. M. Veary	2010	A study of preslaughter pig handling and stunning in selected South African Highveld Region abattoirs	J S Afr Vet Assoc	10.4102/jsava.v81i2.114
759.	D. L. Teixeira; S. Harley; A. Hanlon; N. E. O'Connell; S. J. More; E. G. Manzanilla; L. A. Boyle	2016	Study on the Association between Tail Lesion Score, Cold Carcass Weight, and Viscera Condemnations in Slaughter Pigs	Front Vet Sci	10.3389/fvets.2016.00024

760.	T. M. Stricker; C. Weissenbacher-Lang; M. Ritzmann; A. Ladinig	2013	A study to assess the compatibility of simultaneous use of porcine reproductive and respiratory syndrome virus (EU-type) vaccine and Mycoplasma hyopneumoniae vaccine under field conditions	Berl Munch Tierarztl Wochenschr	
761.	A. Dalmau; P. Rodriguez; P. Llonch; A. Velarde	2010	Stunning pigs with different gas mixtures: aversion in pigs	Animal Welfare	
762.	A. Dalmau; P. Llonch; P. Rodriguez; J. L. Ruiz-de-la-Torre; X. Manteca; A. Velarde	2010	Stunning pigs with different gas mixtures: gas stability	Animal Welfare	
763.	P. Llonch; P. Rodríguez; M. Gispert; A. Dalmau; X. Manteca; A. Velarde	2012	Stunning pigs with nitrogen and carbon dioxide mixtures: effects on animal welfare and meat quality	Animal	10.1017/s1751731111001911
764.	M. Machtolf; M. Moje; K. Troeger; M. Bulte	2013	Stunning slaughter pigs with helium compared to carbon dioxide	Fleischwirtschaft	
765.	D. Mota-Rojas; D. Bolanos-Lopez; M. Concepcion-Mendez; J. Ramirez-Telles; P. Roldan-Santiago; S. Flores-Peinado; P. Mora-Medina	2012	Stunning Swine with CO2 Gas: Controversies Related to Animal Welfare	International Journal of Pharmacology	10.3923/ijp.2012.141.151
766.	K. Troeger	2011	Supervision of an effective sticking and bleeding of slaughter pigs by means of an infrared camera	Fleischwirtschaft	
767.	V. Artuso-Ponte	2015	Supplementation with Quaternary Benzo(c)phenanthridine Alkaloids Decreased Salivary Cortisol and Salmonella Shedding in Pigs After Transportation to the Slaughterhouse	Foodborne pathogens & disease. 2015 Nov. 01, v. 12, no. 11	
768.	G. Cima	2012	Supreme Court hears arguments on downed swine	J Am Vet Med Assoc	10.2460/javma.240.1.8
769.	G. Cima	2012	Supreme Court strikes down Calif. slaughter rules	J Am Vet Med Assoc	
770.	T. Wallgren; R. Westin; S. Gunnarsson	2016	A survey of straw use and tail biting in Swedish pig farms rearing undocked pigs	Acta Vet Scand	10.1186/s13028-016-0266-8
771.	G. Doonan; G. Benard; N. Cormier	2014	Swine veterinarians are a vital resource for minimizing the incidence of stressed pigs during transport	Can Vet J	
772.	N. U. Corrales	2018	Swine welfare at slaughterhouses in Valle de Aburrá (Colombia)	Veterinary and Animal Science. 2018 Dec., v. 6	
773.	J. M. Rommers; I. C. d. Jong; K. H. d. Greef	2015	Symposium on Housing and Diseases of Rabbits, furbearing animals and pet animals		
774.	M. Drag; M. B. Hansen; H. N. Kadarmideen	2018	Systems genomics study reveals expression quantitative trait loci, regulator genes and pathways associated with boar taint in pigs	PLoS One	10.1371/journal.pone.0192673
775.	M. Salines; N. Rose; M. Andraud	2020	Tackling hepatitis E virus spread and persistence on farrow-to-finish pig farms: Insights from a stochastic individual-based multi-pathogen model	Epidemics	10.1016/j.epidem.2019.100369
776.	A. Scollo; G. Di Martino; L. Bonfanti; A. L. Stefani; E. Schiavon; S. Marangon; F. Gottardo	2013	Tail docking and the rearing of heavy pigs: The role played by gender and the presence of straw in the control of tail biting. Blood parameters, behaviour and skin lesions	Research in Veterinary Science	10.1016/j.rvsc.2013.06.019
777.	E. Nannoni; T. Valsami; L. Sardi; G. Martelli	2014	Tail docking in pigs: a review on its short- and long-term consequences and effectiveness in preventing tail biting	Italian Journal of Animal Science	10.4081/ijas.2014.3095
778.	A. L. Vom Brocke; C. Karnholz; D. Madey-Rindermann; M. Gauly; C. Leeb; C. Winckler; L. Schrader; S. Dippel	2019	Tail lesions in fattening pigs: relationships with postmortem meat inspection and influence of a tail biting management tool	Animal	10.1017/s1751731118002070
779.	N. R. Taylor; D. C. J. Main; M. Mendl; S. A. Edwards	2010	Tail-biting: a new perspective	Veterinary journal (London, England : 1997)	

780.	J. Brünger; S. Dippel; R. Koch; C. Veit	2018	'Tailception': using neural networks for assessing tail lesions on pictures of pig carcasses	Animal : an international journal of animal bioscience	
781.	M. L. V. Larsen; A. Gustafsson; J. N. Marchant-Forde; A. Valros	2019	Tear staining in finisher pigs and its relation to age, growth, sex and potential pen level stressors	Animal	10.1017/s1751731118003646
782.	C. Wildraut	2018	Technik als Beitrag für mehr Tierwohl in der Schweinehaltung aus Sicht von Landwirten	Agrar- und Ernährungswirtschaft zwischen Ressourceneffizienz und gesellschaftlichen Erwartungen : 57. Jahrestagung der Gesellschaft für Wirtschafts - und Sozialwissenschaften des Landbaues e.V. vom 13. bis 15. September 2017 , p. 147-158	
783.	T. Hergt; F. Ostner; S. Klein; S. Zöls; M. Erhard; S. Reese; M. Ritzmann; D. Patzkéwitsch	2019	Technopathien der Gliedmaßen bei Mastschweinen: Ursa chen, Entstehung und Tierschutzrelevanz	Tierarztliche Praxis. Ausgabe G, Grosstiere/Nutztiere	
784.	F. Ostner; T. Hergt; S. Klein; D. Patzkéwitsch; S. Reese; A. Brühschwein; A. Meyer-Lindenberg; B. Schade; B. Böhm; R. Eisenreich; A. Rostalski; K.-U. Götz; M. Erhard; M. Ritzmann; S. Zöls	2018	Technopathien der Gliedmaßen bei Mastschweinen: Ursachen, Entstehung, Tierschutzrelevanz	Tierarztliche Praxis. Ausgabe G, Grosstiere/Nutztiere	
785.	J. A. Brown	2011	Temperature and Humidity Conditions in Trucks Transporting Pigs in Two Seasons in Eastern and Western Canada	Transactions of the ASABE. 2011 , v. 54, no. 6	
786.	J. McGlone; A. Johnson; A. Sapkota; R. Kephart	2014	Temperature and Relative Humidity Inside Trailers During Finishing Pig Loading and Transport in Cold and Mild Weather	Animals (Basel)	10.3390/ani4040583
787.	D. B. Jensen	2016	Temperature as a predictor of fouling and diarrhea in slaughter pigs	Livestock science. 2016 Jan., v. 183	
788.	K. Yano; S. Seto; H. Kamioka; K. Mizoi; T. Ogihara	2019	Testosterone and androstenedione are endogenous substrates of P-glycoprotein	Biochem Biophys Res Commun	10.1016/j.bbrc.2019.09.067
789.	M. Erhard	2018	Themenheft zum _Tierschutz beim Schwein_	Tierärztliche Praxis Ausgabe G: Großtiere / Nutztiere	
790.	D. Mota-Rojas; H. Orozco-Gregorio; M. Gonzalez-Lozano; P. Roldan-Santiago; R. Martinez-Rodriguez; M. Sanchez-Hernandez; M. E. Trujillo-Ortega	2011	Therapeutic Approaches in Animals to Reduce the Impact of Stress During Transport to the Slaughterhouse: A Review	International Journal of Pharmacology	10.3923/ijp.2011.568.578
791.	H. Tamada; T. Komuro	2011	Three-dimensional demonstration of the interstitial cells of Cajal associated with the submucosal plexus in guinea-pig caecum	Cell Tissue Res	10.1007/s00441-011-1143-4
792.	D. Olson; F. Wäckers; J.-E. Haugen	2012	Threshold detection of boar taint chemicals using parasitic wasps	Journal of food science	

793.		2016	Tiergerechtheit. Ein Forschungs- und Entwicklungspilotprojekt soll eine standardisierte und automatisierte Erfassung von Tierschutzindikatoren beim Schwein am Schlachthof realisieren. Ziel ist es, das kameragestützte System Ende 2016 zur Marktreife zu bringen	Fleischwirtschaft	
794.		2017	Tiermediziner über das Schlachten _Die meisten Tiere leiden zu viel_: Im Interview : Michael Marahrens		
795.	K. Dörfler; M. Machtolf	2013	Tierschutz erfordert neue Lösungen IFFA 2013: Entwicklungen in der Rinder- und Schweineschlachttechnologie		
796.	L. Schrader	2013	Tierschutz in der Nutztierhaltung - wo liegen Chancen und Grenzen? ; Farm animal welfare - possibilities and restrictions		
797.	F. Hänsch	2012	Tierschutz in der Schweinehaltung - (k)eine Frage der Betriebsgröße?	Amtstierärztlicher Dienst und Lebensmittelkontrolle	
798.		2018	Tierschutz. Kamerasystem standardisiert Auswertung. Automatisierte Erfassung von Tierschutzindikatoren beim Schwein am Schlachthof ist realisierbar	Fleischwirtschaft	
799.	T. Blaha	2015	Tierschutz/Tierhaltung. Schwanzbeißen beim Schwein. Verzicht auf das routinemäßige Schwänzekupieren	Amtstierärztlicher Dienst und Lebensmittelkontrolle	
800.		2017	Tierschutzindikatoren beim Schwein am Schlachthof - Entwicklung einer automatisierten Erfassung. Halter und Halterinnen landwirtschaftlicher Nutztiere in Deutschland müssen dafür Sorge tragen, dass die Gesundheit und das Wohlbefinden der Tiere gewährleistet sind und Schmerzen, Leiden und Schäden vermieden werden. Zur Dokumentation und Evaluation sind nach § 11 Abs. 8 Tierschutzgesetz von Tierhalterinnen und Tierhaltern im Rahmen der Eigenkontrolle geeignete tierbezogene Merkmale, die Tierschutzindikatoren, zu erheben und zu bewerten, um die Anforderungen des § 2 Tierschutzgesetz, also beispielsweise angemessene Ernährung, Pflege, verhaltensgerechte Unterbringung und artgemäße Bewegung, sicherzustellen	Rundschau für Fleischhygiene und Lebensmittelüberwachung	
801.	K. Cimer; S. March; J. Brinkmann; S. Fetscher; D. Gieseke; L. Schrader; A. Schubbert; U. Schultheiß; R. Zapf; U. Knierim	2019	Tierschutzindikatoren für die betriebliche Eigenkontrolle - Impulse für die Ökologische Landwirtschaft		
802.	M. Hungerkamp	2018	Tierwohl Schwanzbeißen vermeiden		

803.	H. Heise	2017	Tierwohl als Differenzierungsstrategie zur Erreichung einer höheren Prozessqualität in der Schweinefleischproduktion?<br>die Sicht der Landwirte	Heutige und zukünftige Herausforderungen an die Qualitätswissenschaft in Forschung und Praxis : Bericht zur GQW-Jahrestagung 2017 in Erlangen , p. 153-183	
804.	A. Schubbert; U. Schultheiß; R. Zapf	2020	Tierwohl im Blick		
805.	R. Zapf; U. Schultheiß; U. Knierim; J. Brinkmann; L. Schrader	2017	Tierwohl messen im Nutztierbestand - Leitfäden für die betriebliche Eigenkontrolle ; Assessing farm animal welfare _ guidelines for on-farm self-assessment		
806.	A. Schubbert; L. Schrader	2018	Tierwohl messen in der landwirtschaftlichen Praxis ; Animal welfare assessment on farms		
807.	C. Gröner; A. Bergschmidt	2019	Tierwohl-Förderprämien der Bundesländer<br>Ausgestaltung, Inanspruchnahme und Reichweite		
808.		2019	Tierwohl-Label für Schweine	Ernährung aktuell	
809.	L. Schrader	2011	Tierwohl-Standards für die Schweinehaltung		
810.	C. Unterweger; M. Wieland; J. Baumgartner	2015	The time and methods for emergency killing of pigs	Wiener Tierarztliche Monatsschrift	
811.	M. Villarroel	2011	Time derivatives in air temperature and enthalpy as non-invasive welfare indicators during long distance animal transport	Biosystems engineering. 2011 Nov., v. 110, issue 3	
812.	M. Sistkova; A. Dolan; J. Broucek; P. Bartos	2015	Time of day and season affect the level of noise made by pigs kept on slatted floors	Archiv Fur Tierzucht-Archives of Animal Breeding	10.5194/aab-58-185-2015
813.	M. Verhoeven; M. Gerritzen; A. Velarde; L. Hellebrekers; B. Kemp	2016	Time to Loss of Consciousness and Its Relation to Behavior in Slaughter Pigs during Stunning with 80 or 95% Carbon Dioxide	Front Vet Sci	10.3389/fvets.2016.00038
814.	E. Voslarova; V. Vecerek; A. Passantino; P. Chloupek; I. Bedanova	2017	Transport losses in finisher pigs: impact of transport distance and season of the year	Asian-Australas J Anim Sci	10.5713/ajas.16.0265
815.	J. J. McGlone; A. K. Johnson; A. Sapkota; R. K. Kephart	2014	Transport of Market Pigs: Improvements in Welfare and Economics	Livestock Handling and Transport, 4th Edition	
816.	E. Lambooij	2014	Transport of Pigs	Livestock Handling and Transport 4th Edition ; ISBN: 9781780643212	
817.	G. Fiore; J. Hofherr; F. Natale; S. Mainetti; E. Ruotolo	2012	Transport temperatures observed during the commercial transportation of animals	Veterinaria italiana	
818.	S. C. Wille; G. Busch; A. Spiller	2017	Transportation in Pig Husbandry: Does an Increase in Consumers' Information and Knowledge Lead to a More Positive Attitude?	German Journal of Agricultural Economics	
819.	K. K. Fogsgaard; M. S. Herskin; K. Thodberg	2018	Transportation of cull sows-a descriptive study of the clinical condition of cull sows before transportation to slaughter	Translational Animal Science	10.1093/tas/txy057
820.	K. Thodberg; K. K. Fogsgaard; M. S. Herskin	2019	Transportation of Cull Sows-Deterioration of Clinical Condition From Departure and Until Arrival at the Slaughter Plant	Front Vet Sci	10.3389/fvets.2019.00028

821.	S. Torrey; R. Bergeron; T. Widowski; N. Lewis; T. Crowe; J. A. Correa; J. Brown; H. W. Gonyou; L. Faucitano	2013	Transportation of market-weight pigs: I. effect of season, truck type, and location within truck on behavior with a two-hour transport	J Anim Sci	10.2527/jas.2012-6005
822.	S. Torrey; R. Bergeron; L. Faucitano; T. Widowski; N. Lewis; T. Crowe; J. A. Correa; J. Brown; S. Hayne; H. W. Gonyou	2013	Transportation of market-weight pigs: II. effect of season and location within truck on behavior with an eight-hour transport	J Anim Sci	10.2527/jas.2012-6006
823.	T. Shu; B. Endong; R. S. Karim; N. Bernhard; H. Jörg	2014	Transportation Stress and Expression of Heat Shock Protein Affecting Pork Quality	Pakistan Veterinary Journal, Vol 34, Iss 1, Pp 112-	
824.	M. Marahrens; E. Stehle; K. v. Deylen; I. Schwarzlose; G. Brümmer	2018	Transportstress bei Schweinen<br>Wie lange darf die Reise sein?: Ein Projekt des Friedrich-Loeffler-Instituts mit Unterstützung der Tönnies Forschung		
825.	A. Bulens; S. Van Beirendonck; J. Van Thielen; N. Buys; B. Driessen	2017	A two-level pen for fattening pigs: Effects on behavior, performance, and postslaughter measurements	J Anim Sci	10.2527/jas.2016.0831
826.	H. Tamada; T. Komuro	2012	Ultrastructural characterization of interstitial cells of Cajal associated with the submucosal plexus in the proximal colon of the guinea pig	Cell Tissue Res	10.1007/s00441-011-1312-5
827.	L. Reidt	2017	Umgang mit Schlachttieren<br>Gestresstes Schwein schmeckt nicht gut		
828.	D. Kümmerlen; S. Hartmann; A. Riklin; R. Figi; X. Sidler	2019	Untersuchung von Tiergesundheit, Tierwohl und Biosicherheit während 101 Transporten von Mastferkeln in der Schweiz	Schweizer Archiv fur Tierheilkunde	
829.	P. Paarlberg	2014	UPDATED ESTIMATED ECONOMIC WELFARE IMPACTS OF PORCINE EPIDEMIC DIARRHEA VIRUS (PEDV)		
830.	C. R. G. Lewis	2010	Use of a putative maternal pheromone during transport and the effect of trailer temperatures on pig losses and welfare	Animal production science. 2010, v. 50, no. 10	
831.	M. Piñeiro; J. Morales; E. Vizcaíno; J. A. Murillo; T. Klauke; B. Petersen; C. Piñeiro	2013	The use of acute phase proteins for monitoring animal health and welfare in the pig production chain: the validation of an immunochromatographic method for the detection of elevated levels of pig-MAP	Meat Sci	10.1016/j.meatsci.2013.03.013
832.	G. Martinic	2011	The use of animals in live-tissue trauma training and military medical research	Lab Anim (NY)	10.1038/laban1011-319
833.	J. Y. Chou; R. B. D'Eath; D. A. Sandercock; N. Waran; A. Haigh; K. O'Driscoll	2018	Use of different wood types as environmental enrichment to manage tail biting in docked pigs in a commercial fully-slatted system	Livestock Science	10.1016/j.livsci.2018.04.004
834.	L. N. Edwards; T. Grandin; T. E. Engle; S. P. Porter; M. J. Ritter; A. A. Sosnicki; D. B. Anderson	2010	Use of exsanguination blood lactate to assess the quality of pre-slaughter pig handling	Meat Sci	10.1016/j.meatsci.2010.05.022
835.	A. Dalmau; J. Pallisera; C. Pedernera; I. Muñoz; R. Carreras; N. Casal; E. Mainau; P. Rodriguez; A. Velarde	2016	Use of high concentrations of carbon dioxide for stunning rabbits reared for meat production	World Rabbit Science, Vol 24, Iss 1, Pp 25-	
836.	A. V. Weschenfelder; L. Saucier; X. Maldague; L. M. Rocha; A. L. Schaefer; L. Faucitano	2013	Use of infrared ocular thermography to assess physiological conditions of pigs prior to slaughter and predict pork quality variation	Meat Sci	10.1016/j.meatsci.2013.06.003

837.	M. Battini; S. Barbieri; A. Vieira; E. Can; G. Stilwell; S. Mattiello	2018	The Use of Qualitative Behaviour Assessment for the On-Farm Welfare Assessment of Dairy Goats	Animals	10.3390/ani8070123
838.	S. Stemkens-Sevens; K. van Berkel; I. de Greeuw; B. Snoeijer; K. Kramer	2009	The use of radiotelemetry to assess the time needed to acclimatize guineapigs following several hours of ground transport	Laboratory animals	
839.	P. Erik; R. Marta; D. H. Amy; E. A. Judy	2017	Use of Temperature, Humidity, and Slaughter Condemnation Data to Predict Increases in Transport Losses in Three Classes of Swine and Resulting Foregone Revenue	Frontiers in Veterinary Science, Vol	
840.	M. Vitali; S. Conte; M. Lessard; K. Deschêne; M. O. Benoit-Biancamano; C. Celeste; G. Martelli; L. Sardi; F. Guay; L. Faucitano	2017	Use of the spectrophotometric color method for the determination of the age of skin lesions on the pig carcass and its relationship with gene expression and histological and histochemical parameters	Journal of animal science	
841.	H. Stein; J. Schulz; R. Morgenstern; T. Voglmayr; G. Freymuller; L. Sinn; T. Rumenapf; I. Hennig-Pauka; A. Ladinig	2018	Use of Three Air Samplers for the Detection of PRRSV-1 under Experimental and Field Conditions	Animals	10.3390/ani8120233
842.	C. Dewey; C. Haley; T. Widowski; R. Friendship; J. Sunstrum; K. Richardson	2009	Using data collected for production or economic purposes to research production animal welfare: an epidemiological approach	J Appl Anim Welf Sci	10.1080/10888700902719781
843.	V. S. M. Jonckheer-Sheehy	2012	Validation of a Polar® human heart rate monitor for measuring heart rate and heart rate variability in adult dogs under stationary conditions	Journal of veterinary behavior. 2012 , v. 7, no. 4	
844.	D. Escribano; M. Fuentes-Rubio; J. J. Ceron	2012	Validation of an automated chemiluminescent immunoassay for salivary cortisol measurements in pigs	Journal of Veterinary Diagnostic Investigation	10.1177/1040638712455171
845.	L. M. Rocha; N. Devillers; X. Maldague; F. Z. Kabemba; J. Fleuret; F. Guay; L. Faucitano	2019	Validation of Anatomical Sites for the Measurement of Infrared Body Surface Temperature Variation in Response to Handling and Transport	Animals	10.3390/ani9070425
846.	N. van Staaveren; B. Doyle; E. G. Manzanilla; J. A. Calderón Díaz; A. Hanlon; L. A. Boyle	2017	Validation of carcass lesions as indicators for on-farm health and welfare of pigs	J Anim Sci	10.2527/jas.2016.1180
847.	J. Niemi; R. Bennett; B. Clark; L. Frewer; P. Jones; T. Rimmler; R. Tranter	2020	A value chain analysis of interventions to control production diseases in the intensive pig production sector	PLoS One	10.1371/journal.pone.0231338
848.	J. Fischer	2015	Verbesserung des Tierschutzes bei der Schweineschlachtung durch ein neu entwickeltes, automatisches Entblutekontrollsystem	Mitteilungsblatt Fleischforschung Kulmbach	
849.	R. Nitzsche; M. Moje; K. Troeger; E. Lücker	2009	Verbesserung des Tierschutzes bei der Schweineschlachtung durch Neugestaltung des Zutriebs zur und in die CO2-Betäubungsanlage		
850.	L. Baldinger; I. Traulsen; F. Weißmann; J. Krieter; R. Bussemas	2017	Vergleich der Injektions- und Inhalationsnarkose zur Kastration von ökologisch aufgezogenen Ferkeln hinsichtlich Verhalten und Wachstum ; Comparison of injection and inhalation anesthesia for castration of organic piglets, with regard to behavior and growth		

851.	B. Bünger; B. Zacharias; H. Schrade	2014	Verhaltensunterschiede bei der Mast von Ebern im Vergleich zu Kastraten und weiblichen Tieren sowie gemischtgeschlechtlichen Gruppen bei unterschiedlichen Haltungs- und Fütterungsbedingungen ; Behavioural differences between entire boars, castrated males, and gilts kept in single or mixed sex groups under different housing and feeding conditions during fattening		
852.	A. Végh; Z. Abonyi-Tóth; P. Rafai	2010	Verification of the technical parameters of head-only electrical stunning of pigs under commercial conditions	Acta Vet Hung	10.1556/AVet.58.2010.2.1
853.	T. Vahlenkamp; C. Baums	2016	Viral-bakterielle Interaktionen bei Erkrankungen im Respirationstrakt unterschiedlicher Haustiere	Pneumologie	
854.		2016	Volles Haus zum Fachgespräch Tierwohl Schwein in Hausstette [Tagungsbericht]		
855.	C. Correia-Gomes; J. I. Eze; J. Borobia-Belsué; A. W. Tucker; D. Sparrow; D. Strachan; G. J. Gunn	2017	Voluntary monitoring systems for pig health and welfare in the UK: Comparative analysis of prevalence and temporal patterns of selected non-respiratory post mortem conditions	Prev Vet Med	10.1016/j.prevetmed.2017.07.007
856.	E. Nannoni	2013	Water requirements of liquid-fed heavy pigs: Effect of water restriction on growth traits, animal welfare and meat and ham quality	Livestock science. 2013 Jan., v. 151, no. 1	
857.		2014	Water sprinkling market pigs in a stationary trailer. 1. Effects on pig behaviour, gastrointestinal tract temperature and trailer micro-climate	Livestock science. 2014 Feb., v. 160	
858.	E. Nannoni; T. Widowski; S. Torrey; J. Fox; L. M. Rocha; H. Gonyou; A. V. Weschenfelder; T. Crowe; G. Martelli; L. Faucitano	2014	Water sprinkling market pigs in a stationary trailer. 2. Effects on selected exsanguination blood parameters and carcass and meat quality variation	Livestock Science	10.1016/j.livsci.2013.11.022
859.	N. V. Bogolyubova; M. G. Chabaev; Y. P. Fomichev; E. Y. Tsis; A. A. Semenova; R. V. Nekrasov	2019	Ways to reduce adverse effects of stress in pigs using nutritional factors	Ukrainian Journal of Ecology	
860.	J. A. Correa	2013	Welfare and carcass and meat quality of pigs being transported for two hours using two vehicle types during two seasons of the year	Canadian journal of plant science. 2013 Mar., v. 93, no. 1	
861.	T. B. Maria Luisa; C. d. A. Juliana; B. William; S. Expedito Tadeu Facco; L. Charlí Beatriz; R. B. Luciano; S. Germano Jorge Domeles	2010	Welfare and meat quality of pigs submitted to differentt pre-slaughter handling techniques Bem-estar e qualidade de carne de suínos submetidos a diferentes técnicas de manejo pré-abate	Revista Brasileira de Saúde e Produção Animal, Vol 11, Iss	
862.		2011	Welfare during transport: EFSA assesses the scientific evidence	Vet Rec	10.1136/vr.d339
863.	M. S. Cockram	2020	Welfare issues associated with the transport of cull sows to slaughter	Vet Rec	10.1136/vr.m547
864.	P. Brandt; M. D. Aaslyng	2015	Welfare measurements of finishing pigs on the day of slaughter: a review	Meat Sci	10.1016/j.meatsci.2014.12.004
865.	P. B. Gade	2011	Welfare of animal production in intensive and organic systems with special reference to Danish organic pig production	Meat science	
866.	L. Rydhmer; M. Hansson; K. Lundström; C. Brunius; K. Andersson	2013	Welfare of entire male pigs is improved by socialising piglets and keeping intact groups until slaughter	Animal : an international journal of animal bioscience	

867.	R. Thomsen	2012	Welfare of entire males and females in organic pig production when reared in single-sex groups	Livestock science. 2012 Nov., v. 149, no. 1-2	
868.	O. Bronwyn; M. Richard; N. Jacqui; W. Mark	2019	The Welfare of Pig-Hunting Dogs in Australia	Animals, Vol 9, Iss 10, p	
869.	A. C. Jorge; G. Harold; T. Stephanie; W. Tina; B. Renée; C. Trever; L. Jean-Paul; F. Luigi	2014	Welfare of Pigs Being Transported over Long Distances Using a Pot-Belly Trailer during Winter and Summer	Animals, Vol 4, Iss 2, Pp 200-	
870.	J. N. Marchant-Forde	2009	Welfare of pigs during transport and slaughter	The welfare of pigs / edited by Jeremy N. Marchant-Forde.	
871.	P. Roldan-Santiago; D. Mota-Rojas; H. Orozco-Gregorio; F. Borderas-Tordesillas; R. Martinez-Rodriguez; P. Mora-Medina; S. Flores-Peinado; M. Sanchez-Hernandez; M. E. Trujillo-Ortega	2015	Welfare of recently weaned piglets transported on unpaved roads: the effect of age and the use of straw bedding	Animal Production Science	10.1071/an13067
872.	T. Grandin	2018	Welfare Problems in Cattle, Pigs, and Sheep that Persist Even Though Scientific Research Clearly Shows How to Prevent Them	Animals (Basel)	10.3390/ani8070124
873.	M. Verhaagh	2019	Wettbewerbsfähigkeit der deutschen Schweineproduktion - Tierwohl / Kastration		
874.	G. A. Carroll	2018	What can carcass-based assessments tell us about the lifetime welfare status of pigs?	Livestock science. 2018 Aug., v. 214	
875.	E. P. Grant; A. Brown; S. L. Wickham; F. Anderson; A. L. Barnes; P. A. Fleming; D. W. Miller	2018	What can the quantitative and qualitative behavioural assessment of videos of sheep moving through an autonomous data capture system tell us about welfare?	Applied Animal Behaviour Science	10.1016/j.applanim.2018.08.010
876.	A. M. S. Huting; K. Almond; I. Wellock; I. Kyriazakis	2017	What is good for small piglets might not be good for big piglets: The consequences of cross-fostering and creep feed provision on performance to slaughter	Journal of Animal Science	10.2527/jas2017.1889
877.	M. L. V. Larsen; H. M. L. Andersen; L. J. Pedersen	2017	Which is the most preventive measure against tail damage in finisher pigs: tail docking, straw provision or lowered stocking density?	Animal : an international journal of animal bioscience	
878.	T. Grandin	2014	A Whole Systems Approach to Assessing Animal Welfare During Handling and Restraint	Livestock Handling and Transport, 4th Edition	

879.	R. Oppermann	2009	Wo steht der Ökologische Landbau heute mit Blick auf zentrale Forderungen der Tierschützer und den tierethischen Diskurs in unserer Gesellschaft? ein Diskussionsbeitrag unter Berücksichtigung von Erfahrungen mit dem Einsatz von Tiergesundheitsplänen in der ökologischen Nutztierhaltung	Praxis trifft Forschung : neues aus der ökologischen Tierhaltung 2009 ; ausgewählte Beiträge der Internationalen Tagungen zur ökologischen Schaf/Ziegen-, Schweine-, Milchkuh- und Geflügelhaltung 2008/2009 ; gemeinsame Veranstaltungen von Bioland e. V. und dem Institut für Ökologischen Landbau des Johann Heinrich von Thünen-Instituts ; [13. Internationale Geflügeltagung, Bio-Geflügel: Tiergesundheit und Lebensmittelsicherheit, 3. - 5. Februar 2009 in Fulda, Deutschland ; 5. Internationale Milchviehtagung, Qualitätsmärkte entwickeln!, 25. - 26. Februar 2009 in Herrsching, Deutschland ; 3. Internationale Schaf- und Ziegentagung, Mit Bioland die Zukunft sichern, 18. - 20. November 2008 in Trenthorst, Deutschland] , p. 7-19	
880.	J. Zili	2012	WSPA and APSRI humane slaughter programme in China: STEPS	Animal welfare. 2012 June, v. 21, no. 2	
881.	C. Bourguet; V. Deiss; A. Boissy; E. M. C. Terlouw	2015	Young Blond d'Aquitaine, Angus and Limousin bulls differ in emotional reactivity: Relationships with animal traits, stress reactions at slaughter and post-mortem muscle metabolism	Applied Animal Behaviour Science	10.1016/j.applanim.2014.12.009

882.	M. Gajęcka; P. Sławuta; J. Nicpoń; R. Kołacz; Z. Kiełbowicz; Ł. Zielonka; M. Dąbrowski; W. Szweda; M. Gajęcki; J. Nicpoń	2016	Zearalenone and its metabolites in the tissues of female wild boars exposed per os to mycotoxins	Toxicon	10.1016/j.toxicon.2016.02.012
883.	G. Franke; V. Bräutigam	2018	Zukunftsfähige Haltung von Mastschweinen. Eine Expertengruppe aus ganz Deutschland hat 20 Stallmodelle entwickelt, die zu mehr Tierwohl und Umweltschutz in der Mastschweinehaltung beitragen sollen. Das Verhalten von Mastschweinen war die Grundlage für die innovativen Konzepte	B & B Agrar	
884.	S. De Luca; E. Zanardi; G. L. Alborali; A. Ianieri; S. Ghidini	2021	Abattoir-Based Measures to Assess Swine Welfare: Analysis of the Methods Adopted in European Slaughterhouses	Animals (Basel)	10.3390/ani11010226
885.	F. Dengu; F. Neri; E. Ogbemudia; G. Ebeling; L. Knijff; K. Rozenberg; R. Dumbill; J. Branchereau; P. Friend; R. Ploeg; J. Hunter		Abdominal multiorgan procurement from slaughterhouse pigs: a bespoke model in organ donation after circulatory death for ex-vivo organ perfusion compliant with the 3 Rs (Reduction, Replacement & Refinement)	ANNALS OF TRANSLATIONAL MEDICINE	10.21037/atm-21-2494
886.	S. S. Nielsen; J. Alvarez; D. J. Bicout; P. Calistri; E. Canali; J. A. Drewe; B. Garin-Bastuji; J. L. Gonzales Rojas; C. Gortázar Schmidt; M. Herskin; M. Miranda Chueca; V. Michel; B. Padalino; P. Pasquali; L. H. Sihvonen; H. Spoolder; K. Stahl; A. Velarde; A. Viltrop; C. Winckler; A. Boklund; A. Botner; A. Gervelmeyer; O. Mosbach-Schulz; H. C. Roberts	2021	Ability of different matrices to transmit African swine fever virus	Efsa j	10.2903/j.efsa.2021.6558
887.	M. Marsot; J. Q. Mei; X. C. Shan; L. Y. Ye; P. Feng; X. J. Yan; C. F. Li; Y. F. Zhao	2020	An adaptive pig face recognition approach using Convolutional Neural Networks	COMPUTERS AND ELECTRONICS IN AGRICULTURE	10.1016/j.compag.2020.105386
888.	J. Pessoa; C. McAloon; M. Rodrigues da Costa; E. García Manzanilla; T. Norton; L. Boyle	2021	Adding value to food chain information: using data on pig welfare and antimicrobial use on-farm to predict meat inspection outcomes	Porcine Health Manag	10.1186/s40813-021-00234-x
889.	Y. Qianying; J. David; T. Lars; Z. Guoqiang; A. Barbara; H. Sabrina; N. t_pán; H. Eberhard; A. Thomas	2020	Airflow Characteristics Downwind a Naturally Ventilated Pig Building with a Roofed Outdoor Exercise Yard and Implications on Pollutant Distribution	Applied Sciences, Vol 10, Iss 4931, p	
890.	R. Stäbler; D. Patzkewitsch; S. Reese; M. Erhard; S. Hartmannsgruber	2020	Alternatives Haltungssystem von Mastschweinen im Wald _ Beurteilung von Tiergesundheit und Schlachtkörperqualität	Berliner und Münchener Tierärztliche Wochenschrift, Vol	
891.	E. Gertzell; U. Magnusson; K. Ikwap; M. Dione; L. Lindström; L. Eliasson-Selling; M. Jacobson	2021	Animal health beyond the single disease approach - A role for veterinary herd health management in low-income countries?	Research in veterinary science	
892.	K. Ekstrand; A. J. Flanagan; I. E. Lin; B. Vejseli; A. Cole; A. P. Lally; R. L. Morris; K. N. Morgan	2021	Animal Transmission of SARS-CoV-2 and the Welfare of Animals during the COVID-19 Pandemic	Animals : an open access journal from MDPI	
893.	S. Atkinson; B. Algers; J. Pallisera; A. Velarde; P. Llonch	2020	Animal Welfare and Meat Quality Assessment in Gas Stunning during Commercial Slaughter of Pigs Using Hypercapnic-Hypoxia (20% CO2 2% O-2) Compared to Acute Hypercapnia (90% CO2 in Air)	ANIMALS	10.3390/ani10122440
894.	J. C. Lange; A. Lange; U. Knierim	2021	Animal Welfare Consequences of Organic Boar Fattening and Occurrence of Boar Taint on Five Commercial Farms	ANIMALS	10.3390/ani11102929

895.	V. J. C. Carrascal; A. D. P. Camacho; V. L. Ayala; A. V. M. Velasquez; P. M. Cajiao; P. J. Cordoba	2021	Animal welfare evaluation at slaughterhouses for pigs at the Eje Cafetero" region in Colombia"	MEAT SCIENCE	10.1016/j.meatsci.2020.108337
896.	M. S. Herskin; K. Overstreet; I. Anneberg	2020	Are veterinary inspections the best way to improve animal welfare during transport?	Vet Rec	10.1136/vr.m3647
897.	M. Verhaagh	2020	Arme Schweine<br>Wirtschaftlichkeit der Alternativen betäubungsloser Ferkelkastration		
898.	D. L. Teixeira; L. C. Salazar; D. Enriquez-Hidalgo; L. A. Boyle	2020	Assessment of Animal-Based Pig Welfare Outcomes on Farm and at the Abattoir: A Case Study	Front Vet Sci	10.3389/fvets.2020.576942
899.	N. Jerez-Timaure; J. Trompiz; E. Mendoza; L. A. de Moreno	2020	Assessment of the stunning method and short lairage time on the carcass and pork quality traits	REVISTA DE INVESTIGACIONES VETERINARIAS DEL PERU	10.15381/rivep.v31i3.18722
900.	D. S. Yang; L. Van Gompel; R. E. C. Luiken; P. Sanders; P. Joosten; E. van Heijnsbergen; I. M. Wouters; P. Scherpenisse; C. Chauvin; K. Wadepohl; G. D. Greve; B. G. M. Jongerius-Gortemaker; M. H. G. Tersteeg-Zijderveld; C. Soumet; M. Skarzynska; K. Juraschek; J. Fischer; D. Wasyl; J. A. Wagenaar; J. Dewulf; H. Schmitt; D. J. Mevius; D. J. J. Heederik; L. A. M. Smit; E. Consortium	2020	Association of antimicrobial usage with faecal abundance of aph(3 ')-III, ermB, sul2 and tetW resistance genes in veal calves in three European countries	INTERNATIONAL JOURNAL OF ANTIMICROBIAL AGENTS	10.1016/j.ijantimicag.2020.106131
901.	D. B. Jensen; L. J. Pedersen	2021	Automatic counting and positioning of slaughter pigs within the pen using a convolutional neural network and video images	COMPUTERS AND ELECTRONICS IN AGRICULTURE	10.1016/j.compag.2021.106296
902.	B. Mathieu; P. Nausicaa; B. David; D. Laurent; C. Laurianne; G. Jean-Luc	2021	Behavior Comparison During Chronic Heat Stress in Large White and Creole Pigs Using Image-Analysis	Frontiers in Animal Science, Vol	
903.	V. M. Urrea; A. M. Bridi; M. C. Ceballos; M. J. R. Paranhos da Costa; L. Faucitano	2021	Behavior, blood stress indicators, skin lesions, and meat quality in pigs transported to slaughter at different loading densities	J Anim Sci	10.1093/jas/skab119
904.	M. Vitali; A. Luppi; P. Bonilauri; E. Spinelli; E. Santacroce; P. Trevisi	2021	Benchmarking of anatomopathological lesions assessed at slaughter and their association with tail lesions and carcass traits in heavy pigs	ITALIAN JOURNAL OF ANIMAL SCIENCE	10.1080/1828051X.2021.1944339
905.		2021	BETRIEBSZWEIG SCHWEIN. Tierwohl. Borchert" ist machbar!"	DLG-Mitteilungen	
906.	J. A. C. Diaz; M. R. da Costa; L. Shalloo; J. K. Niemi; F. C. Leonard; D. Crespo-Piazuelo; J. Gasa; E. G. Manzanilla	2020	A bio-economic simulation study on the association between key performance indicators and pluck lesions in Irish farrow-to-finish pig farms	PORCINE HEALTH MANAGEMENT	10.1186/s40813-020-00176-w
907.	A. M. Swinbourne; K. L. Kind; T. Flinn; D. O. Kleemann; W. H. E. J. van Wettere	2021	Caffeine: A potential strategy to improve survival of neonatal pigs and sheep	Animal reproduction science	
908.	I. Bahelka; O. Bucko; P. Fl'ak	2021	Can Hydrolysable Tannins in Diet of Entire Male Pigs Affect Carcass, Pork Quality Traits, Amino and Fatty Acid Profiles, and Boar Taint, Skatole and Androstenone Levels?	ANIMALS	10.3390/ani11030896
909.	J.-Y. Chou; K. O'Driscoll; D. A. Sandercock; R. B. D'Eath	2020	Can increased dietary fibre level and a single enrichment device reduce the risk of tail biting in undocked growing-finishing pigs in fully slatted systems?	PloS one	

910.	K. Kress; J. Hartung; J. Jasny; V. Stefanski; U. Weiler	2020	Carcass Characteristics and Primal Pork Cuts of Gilts, Boars, Immunocastrates and Barrows Using AutoFOM III Data of a Commercial Abattoir	ANIMALS	10.3390/ani10101912
911.	I. Windschnurer; L. Fischer; T. Yanagida; C. Eibl; S. Franz; S. Waiblinger	2021	Caretaker attitudes and animal training are associated with alpaca behaviour towards humans?An online survey	APPLIED ANIMAL BEHAVIOUR SCIENCE	10.1016/j.applanim.2021.105224
912.	M. López-Arjona; D. Escribano; S. V. Mateo; M. D. Contreras-Aguilar; C. P. Rubio; F. Tecles; J. J. Cerón; S. Martínez-Subiela	2020	Changes in oxytocin concentrations in saliva of pigs after a transport and during lairage at slaughterhouse	Res Vet Sci	10.1016/j.rvsc.2020.08.015
913.	P. K. Roy; A. Y. Qamar; X. Fang; G. Kim; S. Bang; M. De Zoysa; S. T. Shin; J. Cho	2021	Chitosan nanoparticles enhance developmental competence of in vitro-matured porcine oocytes	Reprod Domest Anim	10.1111/rda.13871
914.	C. S. Tsai; Y. P. Hung; J. C. Lee; L. S. Syue; P. R. Hsueh; W. C. Ko	2021	Clostridioides difficile infection: an emerging zoonosis?	Expert Rev Anti Infect Ther	10.1080/14787210.2021.1967746
915.	P.-A. Deutsche	2020	CO2-Betäubung von Schlachtschweinen<br>Suche nach Alternativen		
916.	J. K. Niemi; S. A. Edwards; D. K. Papanastasiou; D. Piette; A. H. Stygar; A. Wallenbeck; A. Valros	2021	Cost-Effectiveness Analysis of Seven Measures to Reduce Tail Biting Lesions in Fattening Pigs	Front Vet Sci	10.3389/fvets.2021.682330
917.	J. N. Marchant-Forde; L. A. Boyle	2020	COVID-19 Effects on Livestock Production: A One Welfare Issue	Front Vet Sci	10.3389/fvets.2020.585787
918.	L. Van Gompel; R. E. C. Luiken; R. B. Hansen; P. Munk; M. Bouwknegt; L. Heres; G. D. Greve; P. Scherpenisse; B. G. M. Jongerius-Gortemaker; M. H. G. Tersteeg-Zijderveld; S. Garcia-Cobos; W. Dohmen; A. Dorado-Garcia; J. A. Wagenaar; B. A. P. Urlings; F. M. Aarestrup; D. J. Mevius; D. J. J. Heederik; H. Schmitt; A. Bossers; L. A. M. Smit	2020	Description and determinants of the faecal resistome and microbiome of farmers and slaughterhouse workers: A metagenome-wide cross-sectional study	ENVIRONMENT INTERNATIONAL	10.1016/j.envint.2020.105939
919.	M. Bernau; S. Schwanitz; L. S. Kreuzer; A. M. Scholz	2021	Detection of Local Tissue Reactions after Anti-GnRF Injection in Male Pigs Assessed Using Magnetic Resonance Imaging	Animals : an open access journal from MDPI	
920.	R. P. Smith; C. Gavin; D. Gilson; R. R. L. Simons; S. Williamson	2020	Determining pig holding type from British movement data using analytical and machine learning approaches	Preventive veterinary medicine	
921.	E. Heyrman; S. Janssens; N. Buys; L. Vanhaecke; S. Millet; F. A. M. Tuyttens; J. Wauters; M. Aluwe	2020	Developing and Understanding Olfactory Evaluation of Boar Taint	ANIMALS	10.3390/ani10091684
922.	R. Watkins; R. Perrott; S. Bate; P. Auton; S. Watts; A. Stoll; S. Rutter; B. Jugg	2021	Development of chlorine-induced lung injury in the anesthetized, spontaneously breathing pig	Toxicol Mech Methods	10.1080/15376516.2021.1906808
923.	E. grosse Beilage	2021	Diagnostik, Ursachen und tierschutzfachliche Bewertung von Kachexien bei Schweinen	Amtstierärztlicher Dienst und Lebensmittelkontrolle	
924.	P. Cybulski; M. Larska; A. Wozniak; A. Jablonski; T. Stadejek	2021	The Dietary Risk Factors of Gastric Ulcers in Finishing Pigs from 16 Polish Farms	AGRICULTURE-BASEL	10.3390/agriculture11080719
925.	L. Alban; M. Vieira-Pinto; D. Meemken; P. Maurer; S. Ghidini; S. Santos; J. G. Laguna; R. Laukkanen-Ninios; O. Alvseike; N. Langkabel	2022	Differences in code terminology and frequency of findings in meat inspection of finishing pigs in seven European countries	FOOD CONTROL	10.1016/j.foodcont.2021.108394

926.	I. Lechner; A. Léger; A. Zimmermann; S. Atkinson; M. Schuppers	2021	Discomfort period of fattening pigs and sows stunned with CO(2): Duration and potential influencing factors in a commercial setting	Meat Sci	10.1016/j.meatsci.2021.108535
927.	R. Uehleke; S. Seifert; S. Huttel	2021	Do Animal Welfare Schemes Promote Better Animal Health? An Empirical Investigation of German Pork Production	LIVESTOCK SCIENCE	10.1016/j.livsci.2021.104481
928.	A. Huneau-Salaün; S. Bougeard; L. Balaine; F. Eono; É. Eveno; M. Guillermic; R. Thomas; N. Rose; F. Pol	2021	Do Rubber Floor Mats Prevent Lameness in Gestating Sows Housed in Large Groups? A Field Experiment on Three Commercial Farms in France	Animals : an open access journal from MDPI	
929.	A. Valros; V. Sali; O. Halli; S. Saari; M. Heinonen	2021	Does weight matter? Exploring links between birth weight, growth and pig-directed manipulative behaviour in growing-finishing pigs	APPLIED ANIMAL BEHAVIOUR SCIENCE	10.1016/j.applanim.2021.105506
930.	D. Werner; L. Baldinger; R. Bussemas; S. Buttner; F. Weissmann; M. Ciulu; J. Morlein; D. Morlein	2021	Early Immunocastration of Pigs: From Farming to Meat Quality	ANIMALS	10.3390/ani11020298
931.	T. Hovmand-Hansen; T. B. Jensen; K. Vestergaard; M. B. F. Nielsen; P. S. Leifsson; H. E. Jensen	2021	Early risk factors, development, disappearance and contents of umbilical outpouching in Danish pigs	LIVESTOCK SCIENCE	10.1016/j.livsci.2021.104654
932.	G. Bonazzi; P. Camanzi; G. Ferri; E. Manghi; M. Iotti	2021	Economic Sustainability of Pig Slaughtering Firms in the Production Chain of Denomination of Origin Hams in Italy	SUSTAINABILITY	10.3390/su13147639
933.	S. H. Mette; D. Todd	2020	Editorial<br>Animal Transport and Related Management	Frontiers in Veterinary Science, Vol	
934.	P. Hartnett; L. A. Boyle; K. O'Driscoll	2020	The effect of group composition and mineral supplementation during rearing on the behavior and welfare of replacement gilts	TRANSLATIONAL ANIMAL SCIENCE	10.1093/tas/txaa002
935.	P. Ciborowska; M. Michalczuk; D. Bie	2021	The Effect of Music on Livestock: Cattle, Poultry and Pigs	Animals : an open access journal from MDPI	
936.	M. Gertz; J. Krieter	2021	Effect of sample size and length of observation period on the reliability of apparent pig organ lesion prevalence	Prev Vet Med	10.1016/j.prevetmed.2021.105258
937.	T. Stempa; G. Bradley	2020	Effect of Sex and Breed on HSPA1A, Blood Stress Indicators and Meat Quality of Lambs	ANIMALS	10.3390/ani10091514
938.	L. Juul; T. Kristensen; P. K. Theil; M. Therkildsen; A. G. Kongsted	2021	Effect of two different feeding strategies on energy intake from pasture, feed efficiency and growth performance of growing-finishing pigs in a mobile pasture system	LIVESTOCK SCIENCE	10.1016/j.livsci.2021.104690
939.	Y. Sasaki; H. Sakurada; M. Yamanaka; K. Nara; S. Tanaka; M. Uema; Y. Ishii; Y. Tamura; T. Asai	2021	Effectiveness of ear skin swabs for monitoring methicillin-resistant Staphylococcus aureus ST398 in pigs at abattoirs	JOURNAL OF VETERINARY MEDICAL SCIENCE	10.1292/jvms.20-0592
940.	J. Kuehling; K. Eisenhofer; M. Lechner; S. Becker; H. Willems; G. Reiner	2021	The effects of boar on susceptibility to swine inflammation and necrosis syndrome in piglets	Porcine Health Manag	10.1186/s40813-021-00194-2
941.	C. Gerlinger; M. Oster; H. Reyer; C. Polley; B. Vollmar; E. Murani; K. Wimmers; P. Wolf	2021	Effects of excessive or restricted phosphorus and calcium intake during early life on markers of bone architecture and composition in pigs	JOURNAL OF ANIMAL PHYSIOLOGY AND ANIMAL NUTRITION	10.1111/jpn.13286
942.	A. W. Duttlinger; K. R. Kpodo; A. P. Schinckel; B. T. Richert; J. S. Johnson	2020	Effects of increasing dietary L-glutamine to replace antibiotics on pig health and performance following weaning and transport	Transl Anim Sci	10.1093/tas/txaa157
943.	N. Čobanović; S. Stajković; B. Blagojević; N. Betić; M. Dimitrijević; D. Vasilev; N. Karabasil	2020	The effects of season on health, welfare, and carcass and meat quality of slaughter pigs	Int J Biometeorol	10.1007/s00484-020-01977-y
944.	K. Tippaya; R. Ngasaman; K. Chukiatsiri	2021	The efficiency of herbal on blood clotting and wound healing of ear notched pigs	THAI JOURNAL OF VETERINARY MEDICINE	10.14456/tjvm.2021.59

945.	J. Husheer; M. Luepke; P. Dziallas; K. H. Waldmann; A. von Altrock	2020	Electrocution as an alternative euthanasia method to blunt force trauma to the head followed by exsanguination for non-viable piglets	ACTA VETERINARIA SCANDINAVICA	10.1186/s13028-020-00565-9
946.	R. Derstappen; I. B. Christoph-Schulz; M. Banse	2021	An empirical analysis of the export potential of pork produced under higher animal welfare standards		
947.	C. Rory	2021	The End of Factory Farming	Voices in Bioethics, Vol	
948.	I. K. Yoo; K. Kim; G. Song; M. Y. Koh; M. S. Lee; A. O. Yeniova; H. Lee; J. Y. Cho	2021	Endoscopic application of mussel-inspired phenolic chitosan as a hemostatic agent for gastrointestinal bleeding: A preclinical study in a heparinized pig model	PLOS ONE	10.1371/journal.pone.0251145
949.	I. Chantziaras; D. De Meyer; L. Vrielinck; T. Van Limbergen; C. Pineiro; J. Dewulf; I. Kyriazakis; D. Maes	2020	Environment-, health-, performance- and welfare-related parameters in pig barns with natural and mechanical ventilation	Prev Vet Med	10.1016/j.prevetmed.2020.105150
950.	C. Crone; F. R. Caldara; R. Martins; G. F. de Oliveira; A. V. Marcon; R. G. Garcia; L. S. Dos Santos; I. Paz; I. C. D. Lippi; M. F. D. Burbarelli		Environmental Enrichment for Pig welfare during Transport	JOURNAL OF APPLIED ANIMAL WELFARE SCIENCE	10.1080/10888705.2021.1983725
951.	A. Kosenko; H. P. Pudollek; D. Brandt; K. W. Paschertz; D. Meemken	2021	Erfassung und Auswertung von Tierwohlindikatoren im Rahmen der amtlichen Schlachttieruntersuchung bei Schlachtschweinen zur Optimierung der Tiergesundheit im Herkunftsbetrieb und zur Anwendung als _Frühwarnsystem_ bei der Fleischuntersuchung	Berliner und Münchener Tierärztliche Wochenschrift, Vol	
952.	J. C. Beasley; L. M. Clontz; A. Rakowski; N. P. Snow; K. C. VerCauteren	2021	Evaluation of a warfarin bait for controlling invasive wild pigs (Sus scrofa)	Pest Manag Sci	10.1002/ps.6351
953.	D. C. Schubert; B. Chuppava; F. Witte; N. Terjung; C. Visscher	2021	Evaluation of Coated Biochar as an Intestinal Binding Agent for Skatole and Indole in Male Intact Finishing Pigs	ANIMALS	10.3390/ani11030760
954.	L. Marsh; M. R. Hutchinson; C. McLaughlan; S. T. Musolino; M. L. Hebart; R. Terry; P. J. Verma; S. Hiendleder; A. L. Whittaker	2021	Evaluation of miRNA as Biomarkers of Emotional Valence in Pigs	Animals : an open access journal from MDPI	
955.	M. Hércules José; L. Clara Mariana Gonçalves; P. Jorge Pamplona; C. Renan Fernandes da; S. Douglas Roberto Guimarães; R. Alcinéia Lemos de Souza; R. Eduardo Mendes	2020	Evaluation of pH in swine carcasses regarding on the trasport distance of the animals<br>a case study	Research, Society and Development, Vol 9, Iss	
956.	F. A. Dalla Costa; T. J. Gibson; S. E. O. Oliveira; N. G. Gregory; A. Coldebella; L. Faucitano; C. B. Ludtke; L. P. Buss; O. A. Dalla Costa	2020	Evaluation of physical euthanasia for neonatal piglets on-farm	J Anim Sci	10.1093/jas/skaa204
957.	M. E. Schoder; M. Tignon; A. Linden; M. Vervaeke; A. B. Cay	2020	Evaluation of seven commercial African swine fever virus detection kits and three Taq polymerases on 300 well-characterized field samples	JOURNAL OF VIROLOGICAL METHODS	10.1016/j.jviromet.2020.113874
958.	M. Heinonen; E. Välimäki; A. M. Laakkonen; I. Toppari; J. Vugts; E. Fàbrega; A. Valros	2021	Evaluation of Tail Lesions of Finishing Pigs at the Slaughterhouse: Associations With Herd-Level Observations	Front Vet Sci	10.3389/fvets.2021.650590
959.	L. Foppa; C. R. Pierozan; S. M. Simonelli; C. P. Dias; C. A. Silva	2021	Evolution of welfare indicators of pigs housed in deep bedding systems during growing and finishing phases: assessment of good health principles and housing conditions	TROPICAL ANIMAL HEALTH AND PRODUCTION	10.1007/s11250-021-02912-y

960.	K. Thodberg; L. M. Gould; S. Støier; I. Anneberg; P. T. Thomsen; M. S. Herskin	2020	Experiences and opinions of Danish livestock drivers transporting sows regarding fitness for transport and management choices relevant for animal welfare	Transl Anim Sci	10.1093/tas/txaa015
961.	M. Aluwe; E. Heyrman; J. M. Almeida; J. Babol; G. Battacone; J. Citek; M. F. I. Furnols; A. Getya; D. Karolyi; E. Kostyra; K. Kress; G. Kusec; D. Morlein; A. Semenova; M. Skrlep; T. Stoyanchev; I. Tomasevic; L. Tudoreanu; M. Van Son; S. Zakowska-Biemans; G. Zamaratskaia; A. Van den Broeke; M. Egea	2020	Exploratory Survey on European Consumer and Stakeholder Attitudes towards Alternatives for Surgical Castration of Piglets	ANIMALS	10.3390/ani10101758
962.	B. Driessen; L. Freson; J. Buyse	2020	Fasting Finisher Pigs before Slaughter Influences Pork Safety, Pork Quality and Animal Welfare	Animals : an open access journal from MDPI	
963.	H. J. Jeon; H. S. Choi; B. Keum; E. J. Bang; K. W. Lee; S. H. Kim; S. Y. Yim; J. M. Lee; E. S. Kim; Y. S. Seo; Y. T. Jeen; H. S. Lee; H. J. Chun; H. B. Kim; J. H. Kim	2021	Feasibility and effectiveness of endoscopic irreversible electroporation for the upper gastrointestinal tract: an experimental animal study	SCIENTIFIC REPORTS	10.1038/s41598-021-94583-w
964.	L. Verband der	2021	Forum angewandte Forschung 2021<br>Neueste Versuchsergebnisse zur Rinder- und Schweinefütterung digital präsentiert: [Ein Tagungsbericht]		
965.	O. Kinane; F. Butler; K. O'Driscoll	2021	Freedom to Grow: Improving Sow Welfare also Benefits Piglets	Animals (Basel)	10.3390/ani11041181
966.	M. Nair-Collins	2021	From the Slaughterhouse to the Laboratory Bench: On the Ethics of Using Slaughtered Animals for Biomedical Research	Perspect Biol Med	10.1353/pbm.2021.0014
967.	P. Cybulski; A. Wozniak; J. Urban; T. Stadejek	2021	Gastric Lesions in Culled Sows: An Underestimated Welfare Issue in Modern Swine Production	AGRICULTURE-BASEL	10.3390/agriculture11100927
968.	H. Morishita; K. Okawa; M. Ishii; K. Mizoi; M. A. Ito; H. Arakawa; K. Yano; T. Ogihara	2020	Gastrointestinal absorption of pimozide is enhanced by inhibition of P-glycoprotein	PLoS One	10.1371/journal.pone.0232438
969.	E. E. Wigham; A. Grist; S. Mullan; S. Wotton; A. Butterworth	2020	Gender and job characteristics of slaughter industry personnel influence their attitudes to animal welfare	ANIMAL WELFARE	10.7120/09627286.29.3.313
970.	S. Larson; A. Arrazola; R. Parra; K. Morrissey; T. Faulkner; M. Jafarikia; I. Mandell; R. Bergeron; R. Lu	2021	Genetic variation in LUMAN/CREB3 and association with stress and meat quality traits in Yorkshire pigs	CANADIAN JOURNAL OF ANIMAL SCIENCE	10.1139/cjas-2020-0156
971.	M. S. Herskin; S. W. Christensen; T. Rousing	2020	Handling and moving cull sows upon arrival at the slaughterhouse-Effects of small versus larger groups of sows	APPLIED ANIMAL BEHAVIOUR SCIENCE	10.1016/j.applanim.2020.105113
972.	J. Grosse-Kleimann; B. Wegner; I. Spiekermeier; E. Grosse Beilage; N. Kemper; H. Nienhoff; H. Plate; H. Meyer; H. Gerhardy; L. Kreienbrock	2021	Health Monitoring of Fattening Pigs - Use of Production Data, Farm Characteristics and On-Farm Examination	Porcine Health Manag	10.1186/s40813-021-00225-y
973.	A. Mrzljak; I. Balen; L. Barbic; M. Ilic; T. Vilibic-Cavlek	2021	Hepatitis E virus in professionally exposed: A reason for concern?	World journal of hepatology	
974.	E. König; V. Sali; P. Heponiemi; S. Salminen; A. Valros; S. Junnikkala; M. Heinonen	2021	Herd-Level and Individual Differences in Fecal Lactobacilli Dynamics of Growing Pigs	Animals (Basel)	10.3390/ani11010113
975.	A. N. da Silva; M. S. Araujo; F. Pertille; A. J. Zanella	2022	How Epigenetics Can Enhance Pig Welfare?	ANIMALS	10.3390/ani12010032

976.	A. Ludwiczak; E. Skrzypczak; J. Składanowska-Baryza; M. Stanisz; P. Ślósarz; P. Racewicz	2021	How Housing Conditions Determine the Welfare of Pigs	Animals (Basel)	10.3390/ani11123484
977.	C. Shari; K. Joel; H. Joel	2020	Humane Euthanasia of Guinea Pigs (Cavia porcellus) with a Penetrating Spring-Loaded Captive Bolt	Animals, Vol 10, Iss 1356, p	
978.	N. A. F. Machado; J. E. Martin; J. A. D. Barbosa-Filho; C. T. S. Dias; D. G. Pinheiro; K. P. L. de Oliveira; J. B. F. Souza-Junior	2021	Identification of trailer heat zones and associated heat stress in weaner pigs transported by road in tropical climates	J Therm Biol	10.1016/j.jtherbio.2021.102882
979.	B. Wagner; K. Royal; R. Park; M. Pairis-Garcia	2020	Identifying Barriers to Implementing Pain Management for Piglet Castration: A Focus Group of Swine Veterinarians	ANIMALS	10.3390/ani10071202
980.	M. Rodrigues da Costa; E. García Manzanilla; A. Diana; N. van Staaveren; A. Torres-Pitarch; L. A. Boyle; J. A. Calderón Díaz	2021	Identifying challenges to manage body weight variation in pig farms implementing all-in-all-out management practices and their possible implications for animal health: a case study	Porcine Health Manag	10.1186/s40813-021-00190-6
981.	O. Mitjana; C. Bonastre; M. T. Tejedor; L. Garza; M. A. Latorre; B. Moreno; M. V. Falceto	2020	Immuno-castration of female and male pigs with anti-gonadotrophin releasing hormone vaccine: Morphometric, histopathological and functional studies of the reproductive system	ANIMAL REPRODUCTION SCIENCE	10.1016/j.anireprosci.2020.106599
982.	C. Weiße; D. Dittmar; B. Jakóbczak; V. Florian; N. Schütze; G. Alber; K. Klose; S. Michalik; P. Valentin-Weigand; U. Völker; C. G. Baums	2021	Immunogenicity and protective efficacy of a Streptococcus suis vaccine composed of six conserved immunogens	Vet Res	10.1186/s13567-021-00981-3
983.	R. M. Park; K. M. Schubach; R. F. Cooke; A. D. Herring; J. S. Jennings; C. L. Daigle	2020	Impact of a cattle brush on feedlot steer behavior, productivity and stress physiology	APPLIED ANIMAL BEHAVIOUR SCIENCE	10.1016/j.applanim.2020.104995
984.	L. Steybe; K. Kress; S. Schmucker; V. Stefanski	2021	Impact of Housing Condition on Welfare and Behavior of Immunocastrated Fattening Pigs (Sus scrofa domestica)	ANIMALS	10.3390/ani11030618
985.	M. Schaeperkoetter; Z. Weller; D. Kness; C. Okkema; T. Grandin; L. Edwards-Callaway	2021	Impacts of group stunning on the behavioral and physiological parameters of pigs and sheep in a small abattoir	Meat Sci	10.1016/j.meatsci.2021.108538
986.	L. Trevisan; J. S. Brum	2020	Incidence of pate, soft and exudative (PSE) pork meat in reason of extrinsic stress factors	ANAIS DA ACADEMIA BRASILEIRA DE CIENCIAS	10.1590/0001-3765202020190086
987.	P. Aymerich; C. Soldevila; J. Bonet; J. Gasa; J. Coma; D. Sola-Oriol	2020	Increasing Dietary Lysine Impacts Differently Growth Performance of Growing Pigs Sorted by Body Weight	ANIMALS	10.3390/ani10061032
988.	I. D. Kusec; E. Cimerman; M. Skrlep; D. Karolyi; K. Gvozdanovic; M. Komlenic; Z. Radisic; G. Kusec	2021	Influence of Immunocastration on Slaughter Traits and Boar Taint Compounds in Pigs Originating from Three Different Terminal Sire Lines	ANIMALS	10.3390/ani11010228
989.	A. Valros; E. Välimäki; H. Nordgren; J. Vugts; E. Fàbrega; M. Heinonen	2020	Intact Tails as a Welfare Indicator in Finishing Pigs? Scoring of Tail Lesions and Defining Intact Tails in Undocked Pigs at the Abattoir	Front Vet Sci	10.3389/fvets.2020.00405
990.	S. Caroline de Aquino; F. Rosemeire da Silva; F. Glaucia Amorin; C. Sanderley Simões da; L. Antonio Carlos de; S. Maria José Dornelas dos	2020	Interference of the transport time between the production farm and the slaughterhouse on the incidence on the bodily injuries and skin damage score in swine carcasses	Research, Society and Development, Vol 9, Iss 7, Pp e977974897-e	
991.	Y. Sasaki; M. Yamanaka; K. Nara; S. Tanaka; M. Uema; T. Asai; Y. Tamura	2020	Isolation of ST398 methicillin-resistant Staphylococcus aureus from pigs at abattoirs in Tohoku region, Japan	JOURNAL OF VETERINARY MEDICAL SCIENCE	10.1292/jvms.20-0184
992.	M. Vitali; E. Nannoni; L. Sardi; G. Martelli	2021	Knowledge and Perspectives on the Welfare of Italian Heavy Pigs on Farms	Animals (Basel)	10.3390/ani11061690

993.	L. Kirner; B. Stürmer	2021	Kosten und Nutzen von mehr Tierwohl. Mehr Tierwohl bedeutet höhere Kosten und niedrigere Gewinne, so die einhellige Meinung. Aber stimmt das immer? Ein Projekt der Wiener Hochschule für Agrar- und Umweltpädagogik hat Kosten in der Schweinehaltung nachgerechnet und persönliche Erfahrungen der Nutztierhalterinnen und -halter mit höheren Tierwohlstandards erfragt	B & B Agrar	
994.	C. Valente; H. Moller; F. M. Johnsen; S. Saxegard; E. R. Brunsdon; O. A. Alvseike	2020	Life cycle sustainability assessment of a novel slaughter concept	JOURNAL OF CLEANER PRODUCTION	10.1016/j.jclepro.2020.122651
995.	S. Zira; L. Rydhmer; E. Ivarsson; R. Hoffmann; E. Roos	2021	A life cycle sustainability assessment of organic and conventional pork supply chains in Sweden	SUSTAINABLE PRODUCTION AND CONSUMPTION	10.1016/j.spc.2021.03.028
996.	M. Vitali; L. Sardi; G. Martelli; E. Nannoni	2021	Literature Review on the Pre-Slaughter Welfare of Italian Heavy Pigs	Animals (Basel)	10.3390/ani11123352
997.	N. Abendschön; S. Senf; P. Deffner; R. Miller; A. Grott; J. Werner; A. M. Saller; J. Reiser; C. Weiß; Y. Zablotski; J. Fischer; S. Bergmann; M. H. Erhard; C. Baumgartner; M. Ritzmann; S. Zöls	2020	Local Anesthesia in Piglets Undergoing Castration-A Comparative Study to Investigate the Analgesic Effects of Four Local Anesthetics Based on Defensive Behavior and Side Effects	Animals (Basel)	10.3390/ani10101752
998.	H. Heise; S. Schwarze	2020	Lohnt sich die Teilnahme an der Initiative Tierwohl? Ergebnisse einer Befragung unter Schweinehaltern		
999.	G. C. Miranda-de la Lama; R. Bermejo-Poza; N. Formoso-Rafferty; M. Mitchell; P. Barreiro; M. Villarroel	2021	Long-Distance Transport of Finisher Pigs in the Iberian Peninsula: Effects of Season on Thermal and Enthalpy Conditions, Welfare Indicators and Meat pH	Animals (Basel)	10.3390/ani11082410
1000.	T. Watanabe; Y. Matsumoto; K. Nishimiya; T. Shindo; H. Amamizu; J. Sugisawa; S. Tsuchiya; K. Sato; S. Morosawa; K. Ohyama; T. Watanabe-Asaka; M. Hayashi; Y. Kawai; J. Takahashi; S. Yasuda; H. Shimokawa	2021	Low-intensity pulsed ultrasound therapy suppresses coronary adventitial inflammatory changes and hyperconstricting responses after coronary stent implantation in pigs in vivo	PLoS One	10.1371/journal.pone.0257175
1001.	G. Go; A. Yoo; S. Kim; J. K. Seon; C. S. Kim; J. O. Park; E. Choi	2021	Magnetization-Switchable Implant System to Target Delivery of Stem Cell-Loaded Bioactive Polymeric Microcarriers	ADVANCED HEALTHCARE MATERIALS	10.1002/adhm.202100068
1002.	E. Pettersson; M. Sjölund; T. Wallgren; E. O. Lind; J. Höglund; P. Wallgren	2021	Management practices related to the control of gastrointestinal parasites on Swedish pig farms	Porcine Health Manag	10.1186/s40813-021-00193-3
1003.	Z. Tobias; R. Martin; K. Achim; H. Christa	2021	Maschinelle Lernverfahren zur Prognose von Tierwohlrisiken in der Schweinehaltung	Landtechnik, Vol 76, Iss	
1004.	F. Dai; E. Dalla Costa; S. Cannas; E. U. L. Heinzl; M. Minero; S. M. Mazzola	2020	May Salivary Chromogranin A Act as a Physiological Index of Stress in Transported Donkeys? A Pilot Study	ANIMALS	10.3390/ani10060972
1005.	B. K. Morris; R. B. Davis; E. Brokesh; D. K. Flippo; T. A. Houser; F. Najar-Villarreal; K. K. Turner; J. G. Williams; A. M. Stelzleni; J. M. Gonzalez	2021	Measurement of the three-axis vibration, temperature, and relative humidity profiles of commercial transport trailers for pigs	JOURNAL OF ANIMAL SCIENCE	10.1093/jas/skab027
1006.	P. T. Muvhali; M. Bonato; A. Engelbrecht; I. A. Malecki; C. Mapiye; S. W. P. Cloete	2020	Meat quality, skin damage and reproductive performance of ostriches exposed to extensive human presence and interactions at an early age	TROPICAL ANIMAL HEALTH AND PRODUCTION	10.1007/s11250-020-02377-5

1007.	G. Temple	2021	Methods to Prevent Future Severe Animal Welfare Problems Caused by COVID-19 in the Pork Industry	Animals, Vol 11, Iss 830, p	
1008.	U. Ämter	2021	Mit Tierwohl Geld verdienen. Höhere Kosten für Tierwohl und eine umweltgerechte Produktion können erfolgreich an Verbraucherinnen und Verbraucher weitergegeben werden. Ob in der Schweinemast oder der Legehennenhaltung - Transparenz fördert Akzeptanz	B & B Agrar	
1009.	M. E. Lagoda; L. A. Boyle; J. Marchewka; J. A. Calderón Díaz	2021	Mixing aggression intensity is associated with age at first service and floor type during gestation, with implications for sow reproductive performance	Animal : an international journal of animal bioscience	
1010.	K. VanderWaal; L. Black; J. Hodge; A. Bedada; S. Dee	2021	Modeling transmission dynamics and effectiveness of worker screening programs for SARS-CoV-2 in pork processing plants	PLoS One	10.1371/journal.pone.0249143
1011.	H. Gray; M. Friel; C. Goold; R. P. Smith; S. M. Williamson; L. M. Collins	2021	Modelling the links between farm characteristics, respiratory health and pig production traits	SCIENTIFIC REPORTS	10.1038/s41598-021-93027-9
1012.	P. K. Roy; A. Y. Qamar; B. M. Tanga; S. Bang; G. Seong; X. Fang; G. Kim; S. L. Edirisinghe; M. De Zoysa; D. H. Kang; I. M. Saadeldin; J. Cho	2021	Modified Spirulina maxima Pectin Nanoparticles Improve the Developmental Competence of In Vitro Matured Porcine Oocytes	ANIMALS	10.3390/ani11092483
1013.	S. E. Starosta; J. C. Schmid; A. Bergschmidt	2021	Monitoring animal welfare in abattoirs Identification and narrowing down of relevant slaughterhouse findings on the basis of an expert survey	BERICHTE UBER LANDWIRTSCHAFT	
1014.	M. Vitali; P. Bosi; E. Santacroce; P. Trevisi	2021	The multivariate approach identifies relationships between pre-slaughter factors, body lesions, ham defects and carcass traits in pigs	PLoS One	10.1371/journal.pone.0251855
1015.	L. Arroyo; D. Valent; R. Carreras; R. Pato; J. Sabrià; A. Velarde; A. Bassols	2020	Neurobiology of environmental enrichment in pigs: hanges in monoaminergic neurotransmitters in several brain areas and in the hippocampal proteome	J Proteomics	10.1016/j.jprot.2020.103943
1016.	A. Kosowska; J. A. Barasona; S. Barroso-Arévalo; B. Rivera; L. Domínguez; J. M. Sánchez-Vizcaíno	2021	A new method for sampling African swine fever virus genome and its inactivation in environmental samples	Sci Rep	10.1038/s41598-021-00552-8
1017.	I. F. M. Font; J. García-Gudiño; M. Izquierdo; A. Brun; M. Gispert; I. Blanco-Penedo; F. I. Hernández-García	2021	Non-destructive evaluation of carcass and ham traits and meat quality assessment applied to early and late immunocastrated Iberian pigs	Animal	10.1016/j.animal.2021.100189
1018.	G. Alexander; B. Ramona; N. Heiko	2021	Observation of dose dependent intravaginal Prostaglandin E2 application in free farrowing sows during parturition _ a pilot study	Porcine Health Management, Vol 7, Iss 1, Pp 1-	
1019.	P. Dolezelova; P. Macakova; P. Chloupek; L. Valkova; Z. Semerad; D. Takacova	2021	The occurrence of technological damage in slaughtered cattle, pigs, sheep and goats in the Czech Republic	ACTA VETERINARIA BRNO	10.2754/avb202190040439
1020.	K. Barington; K. Skovgaard; N. L. Henriksen; H. E. Jensen	2020	Optimising the sampling procedure for forensic investigation of bruises on pigs	Vet Rec	10.1136/vr.105625
1021.	C. Burgeon; M. Debliquy; D. Lahem; J. Rodriguez; A. Ly; M. L. Fauconnier	2021	Past, present, and future trends in boar taint detection	TRENDS IN FOOD SCIENCE & TECHNOLOGY	10.1016/j.tifs.2021.04.007
1022.	V. Vecerek; E. Voslarova; Z. Semerad	2020	Patho-anatomic findings in finisher pigs, sows, and piglets detected during veterinary slaughterhouse inspection	ACTA VETERINARIA BRNO	10.2754/avb202089040341

1023.	M. Forner; R. Cañas-Arranz; S. Defaus; P. de León; M. Rodríguez-Pulido; L. Ganges; E. Blanco; F. Sobrino; D. Andreu	2021	Peptide-Based Vaccines: Foot-and-Mouth Disease Virus, a Paradigm in Animal Health	Vaccines (Basel)	10.3390/vaccines9050477
1024.	D. Werner; K. Hoinghaus; H. Brandt; F. Weissmann; L. Baldinger; R. Bussemas	2020	Performance of organic entire male pigs from two sire lines under two feeding strategies Part 1: Growth performance, carcass quality, and injury prevalence	LANDBAUFORSCHUNG-JOURNAL OF SUSTAINABLE AND ORGANIC AGRICULTURAL SYSTEMS	10.3220/LBF1604659430000
1025.	D. Maes; F. Boyen; B. Devriendt; P. Kuhnert; A. Summerfield; F. Haesebrouck	2021	Perspectives for improvement of Mycoplasma hyopneumoniae vaccines in pigs	Vet Res	10.1186/s13567-021-00941-x
1026.	J. M. Martins; D. Silva; A. Albuquerque; J. Neves; R. Charneca; A. Freitas	2021	Physical Activity Effects on Blood Parameters, Growth, Carcass, and Meat and Fat Composition of Portuguese Alentejano Pigs	ANIMALS	10.3390/ani11010156
1027.	S. Wilhelmsson; M. Andersson; I. Arvidsson; C. Dahlqvist; P. H. Hemsworth; J. Yngvesson; J. Hultgren	2021	Physical workload and psychosocial working conditions in Swedish pig transport drivers	INTERNATIONAL JOURNAL OF INDUSTRIAL ERGONOMICS	10.1016/j.ergon.2021.103124
1028.	A. Rydén; M. Jensen-Waern; G. Nyman; L. Olsén	2021	Physiological and Clinical Responses in Pigs in Relation to Plasma Concentrations during Anesthesia with Dexmedetomidine, Tiletamine, Zolazepam, and Butorphanol	Animals (Basel)	10.3390/ani11061482
1029.	A. K. De; S. Sawhney; P. Ponraj; J. Sunder; S. Banik; D. Bhattacharya	2021	Physiological and immune responses to long road transportation in Andaman local pigs	Trop Anim Health Prod	10.1007/s11250-021-02692-5
1030.	H. R. Golightly; J. Brown; R. Bergeron; Z. Poljak; R. C. Roy; Y. M. Seddon; T. L. O'Sullivan	2021	Physiological response of weaned piglets to two transport durations observed in a Canadian commercial setting	J Anim Sci	10.1093/jas/skab311
1031.	S. Flores-Peinado; D. Mota-Rojas; I. Guerrero-Legarreta; P. Mora-Medina; R. Cruz-Monterrosa; J. Gómez-Prado; M. Guadalupe Hernández; J. Cruz-Playas; J. Martínez-Burnes	2020	Physiological responses of pigs to preslaughter handling: infrared and thermal imaging applications	Int J Vet Sci Med	10.1080/23144599.2020.1821574
1032.	A. Horst; M. Gertz; M. Hasler; J. Krieter	2020	Pig Organ Lesions Recorded in Different Abattoirs: A Statistical Approach to Assess the Comparability of Prevalence	AGRICULTURE-BASEL	10.3390/agriculture10080319
1033.	A. P. Canovas; M. G. Casalduero; A. J. R. Melgarejo	2021	Pigs, accumulation and cheap nature production	RELACIONES INTERNACIONALES-MADRID	10.15366/relacionesinternacionales2021.47.007
1034.	G. Bozzo; B. Padalino; E. Bonerba; R. Barrasso; V. Tufarelli; M. Zappaterra; E. Ceci	2020	Pilot Study of the Relationship between Deck Level and Journey Duration on Plasma Cortisol, Epinephrine and Norepinephrine Levels in Italian Heavy Pigs	ANIMALS	10.3390/ani10091578
1035.	H. Arndt; B. Spindler; S. Hohmeier; J. Hartung; N. Kemper	2021	Planimetric Determination of the Static Space of Cull Sows as the First Step towards a Recommendation of Loading Densities for Cull Sows during Road Transportation in the European Union	AGRICULTURE-BASEL	10.3390/agriculture11010020
1036.	F. Gonzalez-Sole; L. Criado-Mesas; C. Villodre; W. C. Garcia; M. Farre; E. Borda; F. J. Perez-Cano; J. M. Folch; D. Sola-Oriol; J. F. Perez	2020	Porcine Digestible Peptides (PDP) in Weanling Diets Regulates the Expression of Genes Involved in Gut Barrier Function, Immune Response and Nutrient Transport in Nursery Pigs	ANIMALS	10.3390/ani10122368
1037.	E. C. Jongman; R. Woodhouse; M. Rice; J. L. Rault	2021	Pre-slaughter factors linked to variation in responses to carbon dioxide gas stunning in pig abattoirs	Animal	10.1016/j.animal.2020.100134
1038.	L. Sardi; A. Gastaldo; M. Borciani; A. Bertolini; V. Musi; A. Garavaldi; G. Martelli; D. Cavallini; E. Nannoni	2020	Pre-Slaughter Sources of Fresh Meat Quality Variation: The Case of Heavy Pigs Intended for Protected Designation of Origin Products	ANIMALS	10.3390/ani10122386

1039.	H. L. Ko; Q. A. Chong; D. Escribano; I. Camerlink; X. Manteca; P. Llonch	2020	Pre-weaning socialization and environmental enrichment affect life-long response to regrouping in commercially-reared pigs	APPLIED ANIMAL BEHAVIOUR SCIENCE	10.1016/j.applanim.2020.105044
1040.	S. Ghidini; G. L. Alborali; S. De Luca; A. M. Maisano; F. Guadagno; M. Conter; A. Ianieri; E. Zanardi	2021	Predictivity of Antemortem Findings on Postmortem Inspection in Italian Heavy Pigs Slaughterhouses	Animals (Basel)	10.3390/ani11082470
1041.	J. Ma; X. Pan; X. Zhong; Q. Bai; G. Liu; H. Yao	2020	Preferential use of carbon central metabolism and anaerobic respiratory chains in porcine extraintestinal pathogenic Escherichia coli during bloodstream infection	Vet Microbiol	10.1016/j.vetmic.2020.108830
1042.	F. Witte; A. Pajic; F. Menger; I. Tomasevic; D. C. Schubert; C. Visscher; N. Terjung	2021	Preliminary Test of the Reduction Capacity for the Intestinal Adsorption of Skatole and Indole in Weaning Piglets by Pure and Coated Charcoal	ANIMALS	10.3390/ani11092720
1043.	M. Bottacini; A. Scollo; B. Contiero; C. Mazzoni; V. Pace; F. Gottardo	2021	Prevalence of fibrinous pericarditis in heavy pigs (170 kg) and its association with other pluck lesions at slaughter inspection	VETERINARY JOURNAL	10.1016/j.tvjl.2021.105680
1044.	L. Kelbert; R. Stephan; C. Furtwaengler; J. A. Pinillo; M. Morach; M. Nüesch-Inderbinen	2021	Prevalence of Toxoplasma gondii, Hepatitis E Virus, and Salmonella Antibodies in Meat Juice Samples from Pigs at Slaughter in Switzerland	J Food Prot	10.4315/jfp-21-183
1045.	J. Maurin	2020	Quälerei in Schweinebetrieb<br>Bauer knallt Tiere auf Metallkante		
1046.	K. N. Anderson; S. E. Albers; K. J. Allen; K. D. Bishop; B. J. Greco; C. M. Huber; A. A. Kirk; H. Olsen; K. D. Vogel	2021	Quantification of cooling effects on basic tissue measurements and exposed cross-sectional brain area of cadaver heads from market pigs	Transl Anim Sci	10.1093/tas/txab001
1047.	B. L. Novak; J. M. Young; D. J. Newman; A. K. Johnson; S. A. Wagner	2020	A ramp in nursery housing affects nursery pig behavior and speeds loading of market hogs	APPLIED ANIMAL SCIENCE	10.15232/aas.2019-01974
1048.	K. N. Anderson; K. J. Allen; A. Baysinger; M. Benjamin; J. Berger; J. R. Claus; B. J. Greco; E. A. Massie; B. O'Brien; A. Ramirez; A. K. Rendahl; A. A. Reyes; P. E. Zhitnitskiy; K. D. Vogel	2021	Relationship of tissue dimensions and three captive bolt placements on cadaver heads from mature swine (Sus scrofa domesticus) > 200 kg body weight	J Anim Sci	10.1093/jas/skab327
1049.	B. R. McConn; A. W. Duttlinger; K. R. Kpodo; S. D. Eicher; B. T. Richert; J. S. Johnson	2020	Replacing dietary antibiotics with 0.20% l-glutamine and synbiotics following weaning and transport in pigs	J Anim Sci	10.1093/jas/skaa272
1050.	G. D. Brellou; P. D. Tassis; E. P. Apostolopoulou; P. D. Fortomaris; L. S. Leontides; G. A. Papadopoulos; E. D. Tzika	2021	Report on the First African Swine Fever Case in Greece	Vet Sci	10.3390/vetsci8080163
1051.	E. R. Santos; A. M. Bridi; C. A. Silva; B. L. Giangareli; G. A. Ferreira; J. G. Vero; J. A. Fregonesi; S. C. Costa	2021	Reproductive status effects of pair-housed male pigs on natural, agonistic and sexual behaviours	ANIMAL	10.1016/j.animal.2020.100072
1052.	P. Wolf; M. G. Cappai	2020	Response of Fattening Rabbits with Acorns (Quercus pubescensWilld.) Combined in the Diet: First Acquaintances on Growth Performance, Carcass Traits and Perirenal Fatty Acid Profile	ANIMALS	10.3390/ani10081394
1053.	C. Lindahl; E. Sindhoj; R. B. Hellgren; C. Berg; A. Wallenbeck	2020	Responses of Pigs to Stunning with Nitrogen Filled High-Expansion Foam	ANIMALS	10.3390/ani10122210

1054.	J. J. Glaser; L. E. Neidert; C. G. Morgan; M. Brenner; K. S. Stigall; S. Cardin	2020	Resuscitative endovascular balloon occlusion of the aorta for thoracic trauma in the setting of platelet dysfunction: A translational swine study	JOURNAL OF TRAUMA AND ACUTE CARE SURGERY	10.1097/TA.0000000000002882
1055.	L. Guardone; A. Vitali; F. Fratini; S. Pardini; B. T. Cenci Goga; D. Nucera; A. Armani	2020	A Retrospective Study after 10 Years (2010-2019) of Meat Inspection Activity in a Domestic Swine Abattoir in Tuscany: The Slaughterhouse as an Epidemiological Observatory	Animals (Basel)	10.3390/ani10101907
1056.	B. Lebret; M. Čandek-Potokar	2021	Review: Pork quality attributes from farm to fork. Part II. Processed pork products	Animal	10.1016/j.animal.2021.100383
1057.	E. Sindhøj; C. Lindahl; L. Bark	2021	Review: Potential alternatives to high-concentration carbon dioxide stunning of pigs at slaughter	Animal	10.1016/j.animal.2020.100164
1058.	C. Aquilani; A. Confessore; R. Bozzi; F. Sirtori; C. Pugliese	2021	Review: Precision Livestock Farming technologies in pasture-based livestock systems	Animal : an international journal of animal bioscience	
1059.	M. Glatzle	2021	Risikofaktoren für Schwanzverletzungen bei nicht kupierten Mastschweinen	Tierärztliche Praxis Ausgabe G: Großtiere / Nutztiere	
1060.		2020	Schweinehaltung. Tierwohl objektiv messen	DGS	
1061.	Y. H. Zhang; H. Y. Gong; R. S. Mi; Y. Huang; X. G. Han; L. M. Xia; S. F. Li; H. Y. Jia; X. L. Zhang; T. Sun; X. Wang; Z. G. Chen	2020	Seroprevalence of Toxoplasma gondii infection in slaughter pigs in Shanghai, China	PARASITOLOGY INTERNATIONAL	10.1016/j.parint.2020.102094
1062.	N. van Staaveren; L. A. Boyle; E. G. Manzanilla; K. O'Driscoll; L. Shalloo; J. A. C. Díaz	2021	Severe tail lesions in finisher pigs are associated with reduction in annual profit in farrow-to-finish pig farms	Vet Rec	10.1002/vetr.13
1063.	H. L. Ko; S. López-Vergé; Q. Chong; J. Gasa; X. Manteca; P. Llonch	2021	Short communication: Preweaning socialization and environmental enrichment affect short-term performance after regrouping in commercially reared pigs	Animal	10.1016/j.animal.2020.100115
1064.	L. Morgan; J. Meyer; S. Novak; A. Younis; W. A. Ahmad; T. Raz	2021	Shortening sow restraint period during lactation improves production and decreases hair cortisol concentrations in sows and their piglets	Animal	10.1016/j.animal.2020.100082
1065.	C. V. Bradshaw; A. S. Trujillo; S. M. Luecke; L. D. Logan; R. Mohallem; U. K. Aryal; K. R. Stewart; T. M. Casey; R. C. Minor	2021	Shotgun proteomics of homogenate milk reveals dynamic changes in protein abundances between colostrum, transitional, and mature milk of swine	JOURNAL OF ANIMAL SCIENCE	10.1093/jas/skab240
1066.	S. Zira; E. Roos; E. Ivarsson; R. Hoffmann; L. Rydhmer	2020	Social life cycle assessment of Swedish organic and conventional pork production	INTERNATIONAL JOURNAL OF LIFE CYCLE ASSESSMENT	10.1007/s11367-020-01811-y
1067.	M. Andraud; S. Bougeard; T. Chesnoiu; N. Rose	2021	Spatiotemporal clustering and Random Forest models to identify risk factors of African swine fever outbreak in Romania in 2018-2019	Sci Rep	10.1038/s41598-021-81329-x
1068.	T. Hitesh; N. Vipin Venugopal; Y. Sunanda; V. Raghavendra; R. Nilanjan; R. Pankaj	2021	Spontaneous Hematomas in COVID-19 Patients on Low-Molecular-Weight Heparin	Dubai Medical Journal, Vol 4, Iss 3, Pp 285-	
1069.	E. M. C. Terlouw; V. Deiss; T. Astruc	2021	Stunning of pigs with different gas mixtures: Behavioural and physiological reactions	Meat Sci	10.1016/j.meatsci.2021.108452
1070.	S. M. Gulliksen; B. Baustad; T. Framstad; A. Jorgensen; A. Skomsoy; O. Kjelvik; M. Gjestvang; C. A. Grontvedt; B. Lium	2021	Successful eradication of Mycoplasma hyopneumoniae from the Norwegian pig population-10 years later	PORCINE HEALTH MANAGEMENT	10.1186/s40813-021-00216-z

1071.	T. Hovmand-Hansen; S. S. Nielsen; T. B. Jensen; K. Vestergaard; M. B. F. Nielsen; H. E. Jensen	2021	Survival of pigs with different characteristics of umbilical outpouching in a prospective cohort study of Danish pigs	Prev Vet Med	10.1016/j.prevetmed.2021.105343
1072.	A. G. Arruda; T. J. Beyene; J. Kieffer; J. N. Lorbach; S. Moeller; A. S. Bowman	2020	A Systematic Literature Review on Depopulation Methods for Swine	Animals (Basel)	10.3390/ani10112161
1073.	R. Franco; S. Goncalves; M. F. Cardoso; E. Gomes-Neves	2021	Tail-docking and tail biting in pigs: Findings at the slaughterhouse in Portugal	LIVESTOCK SCIENCE	10.1016/j.livsci.2021.104756
1074.	S. Joller; I. M. Häfliger; C. Drögemüller; O. K. Richard; A. Grahofer	2020	Thrombocytopenic purpura on an organic farm with pen mating: a case report on the re-emergence of an old disease	Porcine Health Manag	10.1186/s40813-020-00157-z
1075.	A. Schubbert; U. Schultheiß; R. Zapf	2020	Tierwohl im Blick		
1076.		2020	TIERWOHL UND TIERGESUNDHEIT. Schweinewohl richtig managen. Mit Beobachten, Dokumentieren und Geduld lässt sich einiges für das tierische Wohlergehen tun	Ökologie & Landbau	
1077.	N. Heil; I. Kernberger-Fischer; M. Marahrens; L. Schrader; M. Koch	2021	Tierwohlindikatoren Rind, Schwein und Geflügel _ Transport und Schlachtung / Quelle _Destatis_		
1078.	N. A. F. Machado; J. A. D. Barbosa-Filho; G. L. B. Ramalho; H. Pandorfi; I. J. O. Da Silva	2021	TRAILER HEAT ZONES AND THEIR RELATION TO HEAT STRESS IN PIG TRANSPORT	ENGENHARIA AGRICOLA	10.1590/1809-4430-Eng.Agric.v41n4p427-437/2021
1079.	M. J. Ritter; C. L. Yoder; C. L. Jones; S. N. Carr; M. S. Calvo-Lorenzo	2020	Transport losses in market weight pigs: II. U.S. incidence and economic impact	TRANSLATIONAL ANIMAL SCIENCE	10.1093/tas/txaa041
1080.	K. Dahl-Pedersen; M. S. Herskin	2021	Transportation of Cattle and Pigs between EU Member States 2014-2018 - Can Data from TRACES be used to Create Overview and Inform about Potential Welfare Consequences?	J Appl Anim Welf Sci	10.1080/10888705.2021.1923491
1081.	G. T. Bramblett; J. N. Harris; L. L. Scott; A. W. Holt	2021	Traumatic Optic Nerve Injury Elevates Plasma Biomarkers of Traumatic Brain Injury in a Porcine Model	J Neurotrauma	10.1089/neu.2020.7039
1082.	K. Kauselmann; L. Schrader; B. Glitz; E. Gallmann; H. Schrade; E. T. Krause	2020	Turning the gaze to maize<br>The effects of maize kernels in straw as enrichment on exploration in pigs ; Mais im Fokus: Auswirkungen von Maiskörnern in Stroh als Beschäftigungsmaterial auf die Exploration bei Schweinen		
1083.	R. Espersen; F. C. Falco; P. Hagglund; K. V. Gernaey; A. E. Lantz; B. Svensson	2020	Two novel S1 peptidases from Amycolatopsis keratinophila subsp. keratinophila D2(T) degrading keratinous slaughterhouse by-products	APPLIED MICROBIOLOGY AND BIOTECHNOLOGY	10.1007/s00253-020-10380-x
1084.	T. W. Murphy; R. Cueto; J. P. Zhu; B. Spiess; L. B. Eurell; T. K. Becker	2021	Ultrasound-guided external jugular and femoral arterial cannulation for juvenile swine	LABORATORY ANIMALS	10.1177/00236772211013630
1085.	G. Rubini; E. Nannoni; J. Di Pasquale; G. Martelli; L. Sardi	2021	Update on animal welfare perception by Italian consumers: A descriptive survey	Italian journal of food safety	
1086.	M. Cernat; V. Skampardonis; G. A. Papadopoulos; F. Kroustallas; S. Chalvatzi; E. Petridou; V. Psychas; C. Marouda; P. Fortomaris; L. Leontides	2021	Urinary tract infections in culled sows from Greek herds: prevalence and associations between findings of histopathology, bacteriology and urinalysis	PORCINE HEALTH MANAGEMENT	10.1186/s40813-021-00212-3
1087.	M. krlep; I. Toma_evi; D. Mörlein; S. a. Novakovi; M. Egea; M. D. Garrido; M. B. Linares; I. Peñaranda; M. Aluwé; M. Font-I-Furnols	2020	The Use of Pork from Entire Male and Immunocastrated Pigs for Meat Products-An Overview with Recommendations	Animals : an open access journal from MDPI	

1088.	N. A. F. Machado; J. A. D. Barbosa-Filho; J. B. F. Souza; G. L. B. Ramalho; M. D. M. Parente	2021	USE OF PUPILLOMETRY IN THE DIAGNOSIS OF STRESS IN PIGLETS TRANSPORTED IN A TROPICAL CLIMATE	ENGENHARIA AGRICOLA	10.1590/1809-4430-Eng.Agric.v41n4p402-408/2021
1089.	H. Cuthbertson; G. Tarr; K. Loudon; S. Lomax; P. White; P. McGreevy; R. Polkinghorne; L. A. Gonzalez	2020	Using infrared thermography on farm of origin to predict meat quality and physiological response in cattle (Bos Taurus) exposed to transport and marketing	MEAT SCIENCE	10.1016/j.meatsci.2020.108173
1090.	M. I. Crescio; G. Mastrantonio; S. Bertolini; C. Maurella; A. Adkin; F. Ingravalle; R. R. L. Simons; M. DeNardi; K. Stark; A. Estrada-Peña; G. Ru	2021	Using network analysis to identify seasonal patterns and key nodes for risk-based surveillance of pig diseases in Italy	Transbound Emerg Dis	10.1111/tbed.13960
1091.	X. Averos; B. Balderas; E. Cameno; I. Estevez	2020	The value of a retrospective analysis of slaughter records for the welfare of broiler chickens	POULTRY SCIENCE	10.1016/j.psj.2020.08.026
1092.	G. Temple	2021	The Visual, Auditory, and Physical Environment of Livestock Handling Facilities and Its Effect on Ease of Movement of Cattle, Pigs, and Sheep	Frontiers in Animal Science, Vol	
1093.		2021	Vom Nutz- zum Kuscheltier? Tierwohl in der Schweinehaltung	DGS	
1094.	L. Herrewijn; B. De Groeve; V. Cauberghe; L. Hudders	2021	VR outreach and meat reduction advocacy: The role of presence, empathic concern and speciesism in predicting meat reduction intentions	Appetite	10.1016/j.appet.2021.105455
1095.	E. von Borell; M. Bonneau; M. Holinger; A. Prunier; V. Stefanski; S. Zöls; U. Weiler	2020	Welfare Aspects of Raising Entire Male Pigs and Immunocastrates		
1096.	L. Valkova; V. Vecerek; E. Voslarova; M. Kaluza; D. Takacova	2021	The Welfare of Cattle, Sheep, Goats and Pigs from the Perspective of Traumatic Injuries Detected at Slaughterhouse Postmortem Inspection	Animals (Basel)	10.3390/ani11051406
1097.	S. S. Nielsen; J. Alvarez; D. J. Bicout; P. Calistri; K. Depner; J. A. Drewe; B. Garin-Bastuji; J. L. G. Rojas; C. G. Schmidt; V. Michel; M. A. M. Chueca; H. C. Roberts; L. H. Sihvonen; H. Spoolder; K. Stahl; A. Viltrop; C. Winckler; D. Candiani; C. Fabris; Y. Van der Stede; A. Velarde; E. P. A. H. W. AHAW	2020	Welfare of pigs at slaughter	EFSA JOURNAL	10.2903/j.efsa.2020.6148
1098.	S. S. Nielsen; J. Alvarez; D. J. Bicout; P. Calistri; K. Depner; J. A. Drewe; B. Garin-Bastuji; J. L. G. Rojas; C. G. Schmidt; V. Michel; M. A. M. Chueca; H. C. Roberts; L. H. Sihvonen; H. Spoolder; K. Stahl; A. Viltrop; C. Winckler; D. Candiani; C. Fabris; Y. Van der Stede; A. Velarde; E. P. A. H. W. AHAW	2020	Welfare of pigs during killing for purposes other than slaughter	EFSA JOURNAL	10.2903/j.efsa.2020.6195
1099.	M. Knoll	2020	Wühlareal für mehr Tierwohl bei den Schweinen		
