# Supplementary material for: Impact of Procedures and Human-Animal Interactions during Transport and Slaughter on Animal Welfare of Pigs: A Systematic Literature Review
Source: Animals (Basel). 2022 Dec 2;12(23):3391. doi: 10.3390/ani12233391 (PMC9740978; doi:10.3390/ani12233391)
Supplement: Supplementary file 1 [file animals-12-03391-s001.zip › S4 list of included publications.pdf]

Impact of Procedures and Human-Animal Interactions during Transport and Slaughter on Animal Welfare of Pigs: A Systematic Literature Review

S4: List of included publications

1. Vitali, M.; Bosi, P.; Santacroce, E.; Trevisi, P. The multivariate approach identifies relationships between pre-slaughter factors, body lesions, ham defects and carcass traits in pigs. *PLoS One* **2021**, *16*, 1-14, doi:10.1371/journal.pone.0251855.
2. Rey-Salgueiro, L.; Martinez-Carballo, E.; Fajardo, P.; Chapela, M.J.; Espiñeira, M.; Simal-Gandara, J. Meat quality in relation to swine well-being after transport and during lairage at the slaughterhouse. *Meat Sci.* **2018**, *142*, 38-43.
3. Śmiecińska, K. Slaughter value, meat quality, creatine kinase activity and cortisol levels in the blood serum of growing-finishing pigs slaughtered immediately after transport and after a rest period. *Pol. J. Vet. Sci.* **2011**, *14*, 47-54, doi:10.2478/v10181-011-0007-x.
4. Dokmanovic, M.; Velarde, A.; Tomovic, V.; Glamoclija, N.; Markovic, R.; Janjic, J.; Baltic, M.Z. The effects of lairage time and handling procedure prior to slaughter on stress and meat quality parameters in pigs. *Meat Sci.* **2014**, *98*, 220-226, doi:10.1016/j.meatsci.2014.06.003.
5. Vermeulen, L.; Van de Perre, V.; Permentier, L.; De Bie, S.; Verbeke, G.; Geers, R. Pre-slaughter handling and pork quality. *Meat Sci.* **2014**, *100*, 118-123, doi:10.1016/j.meatsci.2014.09.148.
6. Van de Perre, V.; Permentier, L.; Bie, S.; Verbeke, G.; Geers, R. Effect of unloading, lairage, pig handling, stunning and season on pH of pork. *Meat Sci.* **2010**, *86*, 931-937, doi:10.1016/j.meatsci.2010.07.019.
7. Brandt, P.; Rousing, T.; Herskin, M.S.; Aaslyng, M.D. Identification of post-mortem indicators of welfare of finishing pigs on the day of slaughter. *Livest. Sci.* **2013**, *157*, 1-10.
8. Brandt, P.; Rousing, T.; Herskin, M.S.; Olsen, E.V.; Aaslyng, M.D. Development of an index for the assessment of welfare of finishing pigs from farm to slaughter based on expert opinion. *Livest. Sci.* **2017**, *198*, 65-71, doi:10.1016/j.livsci.2017.02.008.
9. Gerritzen, M.A.; Hindle, V.A.; Steinkamp, K.; Reimert, H.G.M.; van der Werf, J.T.N.; Marahrens, M. The effect of reduced loading density on pig welfare during long distance transport. *Animal* **2013**, *7*, 1849-1857.
10. Dalmau, A.; Tample, D.; Rodriguez, P.; Llonch, P.; Velarde, A. Application of the Welfare Quality® protocol at pig slaughterhouses. *Anim. Welf.* **2009**, *18*, 497-505.
11. von Wenzlawowicz, M.; von Holleben, K.; Eser, E. Identifying reasons for stun failures in slaughterhouses for cattle and pigs: a field study. *Anim. Welf.* **2012**, *21*, 51-60.
12. Stocchi, R.; Mandolini, N.A.; Marinsalti, M.; Cammertoni, N.; Loschi, A.R.; Rea, S. Animal Welfare Evaluation at a Slaughterhouse for Heavy Pigs Intended for Processing. *Ital. J. Food Saf.* **2014**, *3*, 54-56, doi:10.4081/ijfs.2014.1712.
13. Mantis, F.; Bizelis, I.; Symeon, G.K.; Rogdakis, E. Effects of pre-slaughter short-term factors on pork quality. *Anim. Prod. Sci.* **2019**, *59*, 2273-2279, doi:10.1071/an17665.
14. Valkova, L.; Vecerek, V.; Voslarova, E.; Kaluza, M.; Takacova, D. The Welfare of Cattle, Sheep, Goats and Pigs from the Perspective of Traumatic Injuries Detected at Slaughterhouse Postmortem Inspection. *Animals* **2021**, *11*, 2-14.
15. Vermeulen, L.; Van de Perre, V.; Permentier, L.; De Bie, S.; Verbeke, G.; Geers, R. Pre-slaughter sound levels and pre-slaughter handling from loading at the farm till slaughter influence pork quality. *Meat. Sci.* **2016**, *116*, 86-90, doi:10.1016/j.meatsci.2016.02.007.
16. Driessen, B.; Beirendonck, S.V.; Buyse, J. Effects of Housing, Short Distance Transport and Lairage on Meat Quality of Finisher Pigs. *Animals (Basel)* **2020**, *10*, 1-19, doi:10.3390/ani10050788.
17. Brandt, P.; Aaslyng, M.D.; Rousing, T.; Schild, S.L.A.; Herskin, M.S. The relationship between selected physiological post-mortem measures and an overall pig welfare assessment from farm to slaughter. *Livest. Sci.* **2015**, *180*, 194-202, doi:10.1016/j.livsci.2015.07.007.

18. Vitali, A.; Lana, E.; Amadori, M.; Bernabucci, U.; Nardone, A.; Lacetera, N. Analysis of factors associated with mortality of heavy slaughter pigs during transport and lairage. *J. Anim. Sci.* **2014**, *92*, 5134-5141, doi:10.2527/jas.2014-7670.
19. Sardi, L.; Gastaldo, A.; Borciani, M.; Bertolini, A.; Musi, V.; Martelli, G.; Cavallini, D.; Rubini, G.; Nannoni, E. Identification of Possible Pre-Slaughter Indicators to Predict Stress and Meat Quality: A Study on Heavy Pigs. *Animals (Basel)* **2020**, *10*, 1-14, doi:10.3390/ani10060945.
20. Driessen, B.; Beirendonck, S.V.; Buyse, J. Effects of Transport and Lairage on the Skin Damage of Pig Carcasses. *Animals (Basel)* **2020**, *10*, 1-15.
21. Garcia-Diez, J.; Coelho, A.C. Causes and factors related to pig carcass condemnation. *Vet. Med.* **2014**, *59*, 194-201, doi:10.17221/7480-vetmed.
22. Nannoni, E.; Liuzzo, G.; Serraino, A.; Giacometti, F.; Martelli, G.; Sardi, L.; Vitali, M.; Romagnoli, L.; Moscardini, E.; Ostanello, F. Evaluation of pre-slaughter losses of Italian heavy pigs. *Anim. Prod. Sci.* **2017**, *57*, 2072-2081, doi:10.1071/an15893.
23. Čobanović, N.; Karabasil, N.; Stajković, S.; Ilić, N.; Suvajdžić, B.; Petrović, M.; Teodorović, V. The Influence of Pre-Mortem Conditions on Pale, Soft and Exudative (PSE) and Dark, Firm and Dry (DFD) Pork Meat. *Acta Vet. (Beogr)* **2016**, *66*, 1-15.
24. Vecerek, V.; Voslarova, E.; Semerad, Z.; Passantino, A. The Health and Welfare of Pigs from the Perspective of Post Mortem Findings in Slaughterhouses. *Animals* **2020**, *10*, 1-10.
25. López-Arjona, M.; Escribano, D.; Mateo, S.V.; Contreras-Aguilar, M.D.; Rubio, C.P.; Tecles, F.; Cerón, J.J.; Martínez-Subiela, S. Changes in oxytocin concentrations in saliva of pigs after a transport and during lairage at slaughterhouse. *Res. Vet. Sci.* **2020**, *133*, 26-30, doi:10.1016/j.rvsc.2020.08.015.
26. Dalmau, A.; Geverink, N.A.; Nuffel, A.v.; Steenbergen, L.v.; Reenen, C.G.v.; Hautekiet, V.; Vermeulen, K.; Velarde, A.; Tuyttens, F.A.M. Repeatability of lameness, fear and slipping scores to assess animal welfare upon arrival in pig slaughterhouses. *Animal* **2010**, *4*, 804-809.
27. Dalmau, A.; Nande, A.; Vieira-Pinto, M.; Zamprogna, S.; Di Martino, G.; Ribas, J.C.R.; da Costa, M.P.; Halinen-Elemo, K.; Velarde, A. Application of the Welfare Quality protocol in pig slaughterhouses of five countries. *Livest. Sci.* **2016**, *193*, 78-87, doi:10.1016/j.livsci.2016.10.001.
28. Dokmanovic, M.; Ivanovic, J.; Janjic, J.; Boskovic, M.; Laudanovic, M.; Pantic, S.; Baltic, M.Z. Effect of lairage time, behaviour and gender on stress and meat quality parameters in pigs. *Anim. Sci. J.* **2017**, *88*, 500-506, doi:10.1111/asj.12649.
29. Garcia-Celdran, M.; Ramis, G.; Quereda, J.J.; Armero, E. Reduction of transport-induced stress on finishing pigs by increasing lairage time at the slaughter house. *J. Swine Health Prod.* **2012**, *20*, 118-122.
30. Bottacini, M.; Scollo, A.; Edwards, S.A.; Contiero, B.; Veloci, M.; Pace, V.; Gottardo, F. Skin lesion monitoring at slaughter on heavy pigs (170 kg): Welfare indicators and ham defects. *PLoS One* **2018**, *13*, 1-16, doi:10.1371/journal.pone.0207115.
31. Panella-Riera, N.; Gispert, M.; Gil, M.; Soler, J.; Tibau, J.; Oliver, M.A.; Velarde, A.; Fabrega, E. Effect of feed deprivation and lairage time on carcass and meat quality traits on pigs under minimal stressful conditions. *Livest. Sci.* **2012**, *146*, 29-37, doi:10.1016/j.livsci.2012.02.017.
32. Vermeulen, L.; Van de Perre, V.; Permentier, L.; De Bie, S.; Verbeke, G.; Geers, R. Sound levels above 85 dB pre-slaughter influence pork quality. *Meat Sci.* **2015**, *100*, 269-274, doi:10.1016/j.meatsci.2014.10.025.
33. van Staaveren, N.; Teixeira, D.L.; Hanlon, A.; Boyle, L.A. The effect of mixing entire male pigs prior to transport to slaughter on behaviour, welfare and carcass lesions. *PLoS ONE* **2015**, *10*, 1-15.
34. Driessen, B.; Van Beirendonck, S.; Buyse, J. The Impact of Grouping on Skin Lesions and Meat Quality of Pig Carcasses. *Animals (Basel)* **2020**, *10*, 1-11, doi:10.3390/ani10040544.
35. Schild, S.L.A.; Brandt, P.; Rousing, T.; Herskin, M.S. Does the presence of umbilical outpouchings affect the behaviour of pigs during the day of slaughter? *Livest. Sci.* **2015**, *176*, 146-151, doi:10.1016/j.livsci.2015.03.023.

36. Végh, A.; Abonyi-Tóth, Z.; Rafai, P. Verification of the technical parameters of head-only electrical stunning of pigs under commercial conditions. *Acta Vet. Hung.* **2010**, *58*, 147-156, doi:10.1556/AVet.58.2010.2.1.
37. Nodari, S.R.; Polloni, A.; Giacomelli, S.; Vezzoli, F.; Galletti, G. Assessing pig welfare at stunning in Northern Italy commercial abattoirs using electrical method. *Large Anim. Rev.* **2014**, *20*, 87-91.
38. Végh, Á.; Abonyi-Tóth, Z.; Rafai, P. Effect of current intensity and duration on the effectiveness of head-only electrical stunning in pigs under commercial conditions. *Acta Vet. Hung.* **2017**, *65*, 13-28, doi:10.1556/004.2017.002.
39. Atkinson, S.; Velarde, A.; Llonch, P.; Algers, B. Assessing pig welfare at stunning in Swedish commercial abattoirs using CO2 group-stun methods. *Anim. Welf.* **2012**, *21*, 487-495, doi:10.7120/09627286.21.4.487.
